# ‘Baltic catacombs.’ Translating *corpisanti* catacomb relic-sculptures between Rome, Polish Livonia, and the Lithuanian Grand Duchy circa 1750-1800

**DOI:** 10.12688/openreseurope.13259.1

**Published:** 2021-03-24

**Authors:** Radosław Budzyński, Dzianis Filipchyk, Melchior Jakubowski, Dzmitry Marozau, Ruth Sargent Noyes, Vika Veličkaitė

**Affiliations:** 1 Independent Researcher, Lubanie, Poland; 2 Department of Museology, Ethnology and History of Arts, Belarusian State University, Minsk, Belarus; 3 Independent Researcher, Warsaw, Poland; 4 Department of Philosophy and History, Belarusian State University of Physical Culture, Minsk, Belarus; 5 Middle Ages, Renaissance and Numismatics, National Museum of Denmark, Copenhagen, Denmark; 6 Independent Researcher, Vilnius, Lithuania

**Keywords:** history of art, relics, history of religion, Baltic history, Counter-Reformation, Rome

## Abstract

This article offers a first study of the traffic of *corpisanti* catacomb relic-sculptures between Rome and sites in the Grand Duchy of Lithuania and Polish Livonia in the decades just before and during the Age of Partition (c. 1750-1800). The article firstly frames an overview of current knowledge on *corpisanti* more broadly against cases in Livonia and the Grand Duchy. It secondly provides a clearinghouse of secondary and primary source evidence on this topic, with particular attention to providing previously largely unpublished or under-studied texts pertaining to *corpisanti* cults in the north in translation, included as appendices. This article also presents a study in methods of collaborative scholarship in the pandemic era, investigating across distinct genres of source materials and material and artistic cultural heritage objects accessed via scholarly networks both in the field and online, representing historic sites and institutions in present-day Italy, Ukraine, Belarus, Poland, Latvia, and Lithuania.

## Introduction: “the promise of the relics of the Holy Body of some soldier” ^1^

“In the most gracious audience afforded me by His Holiness Pope Pius IV, I received with immense gratitude from His Holiness the promise of the relics of the Holy Body [ *Corpo Santo*] of some soldier. Finding myself close to my departure from Rome, since this is a perfect opportunity to send the body to my own homeland, I have the honor to beseech you to have the generosity to remind His Holiness the Pope of His promise, together with that of some other Relic, particularly that of the Holy Cross, with their authenticating documentation. I ask this begging that your good graces might excuse my own ardor in this matter. I pray for some opportunity to be able to serve His Holiness the Pope, and that He will ask anything from me in the future. With due obsequies I remain your most Devout and Humble servant, Count de Borch.” ^2^

**Figure 1.**
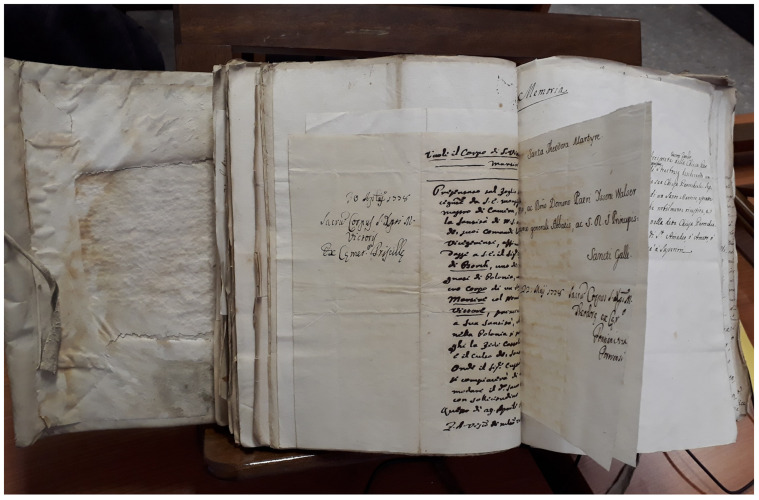
Rome, Archivio Storico della Diocesi di Roma, Archivio Storico del Vicariato di Roma, Archivio del Custode delle SS. Reliquie, Volume 77 (1737–83), “Custodia delle S.S.Reliquie dell’Imo Sigr Card. Vicario di N.S.|Corpi, e Reliquie de’ SS. Martiri Donati|Tomo I. Dall’anno 1737-al 1783|Giacinto Ponzetti Custode.” Image: Ruth Sargent Noyes.

In April 1778 Count Michał Jan Borch (or von der Borch-Lubeschitz und Borchhoff, 1753–1810) submitted this supplication to a representative of the Cardinal Vicar of Rome ^3^ (See also extended data, Appendix 1). His petition for “the Holy Body [ *Corpo Santo*] of some soldier” is today preserved in the Archive of the Custodian of Sacred Relics and Cemeteries (on whom more below) in Roman Diocesan Archives, together with hundreds of other such petitions from Catholic supplicants from all corners of the globe, made between 1737 and 1783 ( Figure 1). The 25 year-old Borch was preparing to conclude a European grand tour begun in 1772 and return to his family’s estates in the historical territory of the Inflanty Voivodeship (Polish: Województwo inflanckie), also known as Polish Livonia, Livonia, or Inflanty ^4^. In fact, Borch would prolong his travels for another three years before departing Italy on the death of his father, perhaps in part due to unstable circumstances back home, which made his situation in the north uncertain and his diplomatic skills at sympathetic Italian courts especially valuable. Today, the geographic area that Borch referred to as his *Patria* (“Homeland”) corresponds to Latgale, the eastern region of Latvia along the Daugava river, marking Baltic Europe’s frontier with Russia ^5^. The same year that Borch departed on his grand tour, however, Inflanty had been translated from an administrative division within the historical territory of the Polish-Lithuanian Commonwealth of the Polish-Lithuanian Commonwealth (consisting of the Grand Duchy and the Crown Kingdom of Poland), to a newly annexed territory of Czarist Russia ^6^. Even as the young nobleman wrote in hopes of bringing back sacred souvenirs, his homeland in a sense existed only in collective memory as a relic of what had been one of early modern Europe’s largest and most diverse states, fragmented and forcibly integrated into a new empire in the first of three so-called Partitions of Poland-Lithuania (1772, 1793, 1795), territorial divisions perpetrated by Russia, Prussia, and Austria that progressively fractured the Commonwealth until the conglomerate state ceased to exist altogether ^7^.

**Figure 2.**
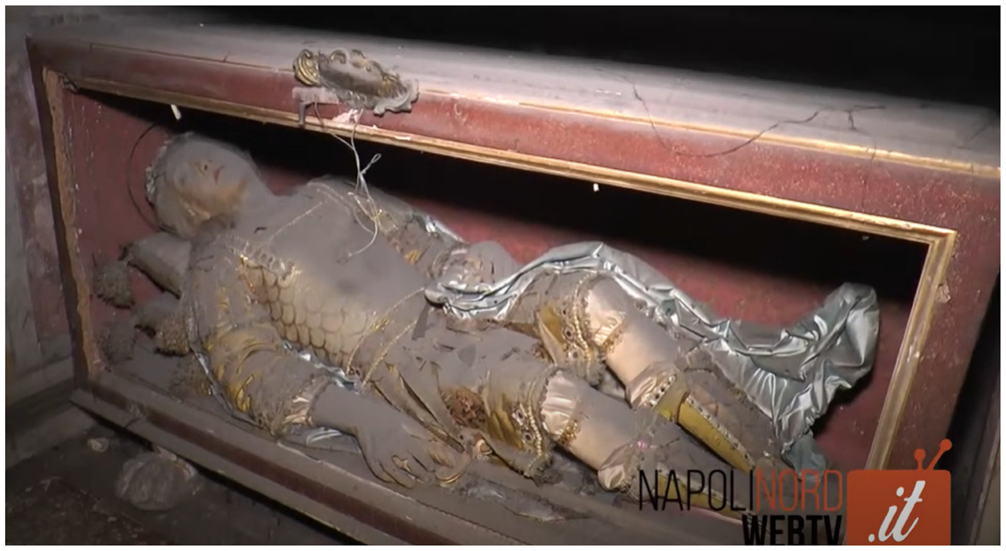
*Corposanto* relic-sculpture of St. Feliciano. Human remains (skeletal fragments) and mixed materials. Circa 1795. Palazzo Pinelli, Giugliano in Campania (Naples), Italy. Image: https://www.napolinordwebtv.it/2017/02/28/viaggio-nella-cappella-santa-maria-addolorata-giugliano-ci-le-spoglie-san-feliciano-martire-video/ (accessed 15 February 2021).

Against the background of the dissection of his *Patria* 2500 kilometers to the north, Borch ostensibly asked for relics in the form of complete skeletal remains of one of the many ancient imperial soldiers supposedly martyred after converting to Christianity. Catholic scholars maintained that legions of them populated subterranean cemeteries beneath the *urbe* ^8^. What he actually would receive might be more accurately characterized as a relic-sculpture or so-called *corposanto* (Italian for “holy body,” plural *corpisanti*) ^9^ ( Figure 2). *Corpisanti* represent a hybrid species of multimedia artwork that integrated relics and reliquary within a single sculptural form and were peculiar to late eighteenth- and early nineteenth-century Rome, where they were serially manufactured under the purview of the papacy and Church agents ^10^. These luxurious fabrications incorporated scant human remains mined from the catacombs within a man-made, life-size anthropomorphic illusion of integral corporality, lavishly attired in courtly regalia and typically fashioned in the guise of a *sponsa Christi* (“bride of Christ”) or *miles Christi* (“soldier of Christ”) ^11^.

Taking Borch’s relic request as a point of departure, this article offers a first study of the traffic of *corpisanti* between Rome and sites in the Grand Duchy and Polish Livonia in the decade just before and during the Age of Partition (c. 1750–1800), a watershed period when the territories of not only the Papal States, but also Livonia and the Grand Duchy were subject to dissolution by imperial powers ^12^. Given the state of scholarship on this under-studied phenomenon, what follows firstly frames an overview of current knowledge on *corpisanti* more broadly against cases in Inflanty and the Grand Duchy. Regarding the use of the terms Grand Duchy of Lithuania and Polish Livonia (or Inflanty), it should be noted that these political-administrative entities represented separate and distinct units within the Polish-Lithuanian Commonwealth, although they constituted a single cultural region and its elites embodied one social group. Given that this article focuses predominantly on cases that were located in Polish Livonia, while cases from the Grand Duchy appear predominantly as contextual background, Polish Livonia will be therefore emphasized as a particular entity. What follows secondly provides a clearinghouse of secondary and primary source evidence on this topic, with particular attention to providing previously largely unpublished or under-studied texts pertaining to *corpisanti* cults in the north in translation, included as appendices ^13^. While a detailed analysis of each of these texts is beyond the purview of the present study, it should be noted that a striking commonality across these writings is a shared mode of address regarding the *corpisanti*, which are often referred to not as things or relics *per se*, but rather as persons, bodies, or protagonists. This phenomenon evinces not only general conventions regarding the cults of saints in the period under investigation, but also the more specific success of these relic-sculptures in projecting the illusion of embodied corporality and indeed personhood. In proceeding, the article also presents a study in methods of collaborative scholarship in the pandemic era, investigating across distinct genres of source materials and material and artistic cultural heritage objects accessed via scholarly networks both in the field and online, representing historic sites and institutions in present-day Italy, Ukraine, Belarus, Poland, Latvia, and Lithuania.

## “treasuries of precious jewels”

When Michał Jan Borch composed his supplication in Rome, his father Lithuanian Grand Chancellor Jan Jędrzej (or Andrzej) Józef Borch (1713–80) ranked among the most powerful Livonian magnates in the shrinking Commonwealth ^14^. Historically, aristocratic clans like the Borch owned and actually governed much of Poland-Lithuania, a conglomerate polity subdivided into a patchwork of patrimonial latifundia—large autonomous estates with private towns, private armies, trading privileges, and proprietary currencies, linked by familial alliances ^15^. Borch’s territorial holdings were concentrated in Inflanty, an area that drew cultural, diplomatic and economic potential from its geographic location along a major waterway and important trade thoroughfare linking Czarist Russia with the Grand Duchy and Baltic sea ports. This strategic position situated its resident magnates as powerful and wealthy patrons who took an active interest in cultivating their cultural and political horizons, building up their estates as centers and satellite courts ^16^.

Like neighboring Polish Livonian clans such as the Plater (on whom more below), Borch descended from venerable Westphalian houses who immigrated north centuries earlier in the medieval Baltic crusades, converted to Lutheranism in the early modern period, and back to Catholicism by the early eighteenth century ^17^. They held numerous honors and posts in government and the Church, and their members enjoyed the polymathic and international educations, were fluent in multiple languages, authored works of literature, science and history, maintained vast correspondence networks, and undertook extensive European grand tours ^18^. The Borch ranked among the only in Polish Livonia to hold the titles of Counts of the Holy Roman Empire, and vigorously cultivated both their crusader origins and ties to the Roman Church, overseeing after the Northern Wars (c. 1650–1720) a web of monuments largely built or re-built that articulated Inflanty’s sacred topography on Europe’s north-eastern frontier, where the long Counter-Reformation negotiated a complex web of intersecting yet potentially opposed political and religious interests in the decades preceding and during the Age of Partition ^19^. In the midst of this instability interrelated magnate families staked for themselves a strategic position as a north-easternmost Roman Catholic stronghold, even as they negotiated the process of transition from the Commonwealth’s political system of nobles to Czarist Russian administrative frameworks and fought to acquire or reconfirm their privileged position within the new state’s distinct linguistic, social, political, cultural and religious structures ^20^. Their cultural campaigns of self-fashioning staged a renovation of *Romanitas* that cultivated literature and historiography, and art and architecture that inflected late baroque and rococo Italianate forms.

Within the complex and changing landscape of Catholic elites in the eighteenth-century Grand Duchy, importing and engaging with sacred relics played a crucial constitutive role in reifying their shifting borderlands zone as *Antemurale Christianitatis* (Bulwark of Christendom) guarding against martial, spiritual and ideological threats on behalf of Catholicism ^21^. Relics could embody power and instantiate identity on both a local and national level in this period ^22^. At the turn of the seventeenth century the Spanish monarchy famously harnessed the prestige of unrivaled collections at El Escorial to bolster royal authority ^23^, in the early seventeenth-century Rome the Oratorian congregation defied papal censure to stage the lavish translation of their founder’s body in the manner of an ancient martyr ^24^, in mid-seventeenth-century Florence the Medici reified the longevity and scope of their dynasty with an encyclopedic collection of first-class relics and reliquaries grouped according to material in special display cabinets ^25^, and at the end of the century in Vilnius (Pol. Wilno) the cathedral chapter vied with scions of the powerful Pac (Lithuanian: Pacas) for control of the precious relics of Polish-Lithuanian saint Kazimierz Jagiellończyk (1458–84, canonized 1602) ^26^. As manifestations of socio-political prestige and power, relics also proved vital agents in period diplomacy between diverse prerogatives: Pope Alexander VII (1599–1667) gifted the remains of St. Zénon to King Louis XIV of France to seal their reconciliation in 1664 ^27^, while the Medici and Pac negotiated a political rapprochement through a mutual exchange of relics of their respective grand ducal patron saints in the 1670–80s ^28^.

According to Counter-Reformation theology reaffirming the cult of relics and saints, thanks to the proximity and multiplicity of their heavenly avatars, the saints, to the Divine, relics enabled the galvanic diffusion of supernatural aura through material traces, such that they were considered not only (or even mainly) as symbols of power, prestige, and piety, but actual *conductors* of divine grace, much like metals conduct electricity ^29^. Within the Roman Catholic cult of relics, more desirable ‘primary’ or ‘first-class’ relics (meaning physical corporeal remains) were especially precious for their proximity to the divine, distinguished according to the Church from ‘secondary’ (objects a holy person used or touched) and ‘tertiary’ (objects in physical contact with one of these secondary objects) ^30^. Canon Law further distinguished between significant ( *insignes*) relics—typically a saint’s entire body or a major body part such as the skull—and non-significant ( *non insignes*) relics ^31^. The Counter-Reformation papacy accorded special attention to primary insigne whole-body relics that demonstrated postmortem bodily incorruption, a miraculous manifestation suggesting the subject was divinely exempt from the physical process of decomposition. Whereas such corporal integrity was historically recognized by the Church as an *indicium sanctitatis* and proof of resurrection, during the seventeenth century the phenomenon became a particularly valued and commonplace sign of holiness amongst new “modern” candidates for canonization, and increasingly confirmed by medical autopsy performed by professional experts under the Vatican’s remit ^32^.

Rather than quelling widespread engagement with relics amongst European elites, the wars and upheaval and advent of the Enlightenment in the eighteenth century instead catalyzed a renewed fervor for the cult of relics amongst elite and well-educated Catholic constituencies, including in the Grand Duchy, where relic translations were fueled by bi-directional local and exterior factors, ranging from the rise of recently Polonised and re-Catholicized magnates like the Borch amidst a religiously diverse polity and manifold loss of or perceived threats to sacral heritage, to neocolonial interests from the perspective of Rome and the Apostolic Chamber attending the long Counter-Reformation ^33^. In this context, catacomb relics held to be the remains of the earliest Paleochristian converts and champions—many thought to be Roman soldiers—who valiantly died for their faith played a particularly charged discursive role ^34^. The rediscovery of the Roman catacombs in 1578 had transformed the burial chambers beneath the papal city into what seemed inexhaustible “treasuries of precious jewels” and “mines of sanctity.” ^35^ Their seemingly countless ranks in the form of bones of the first martyrs constituted a numinous army ready to be mobilized through *translatio* (ritual relocation of relics) in global battles against heresies and for souls, especially in transalpine confessional borderlands and frontier zones ^36^.

**Figure 3.**
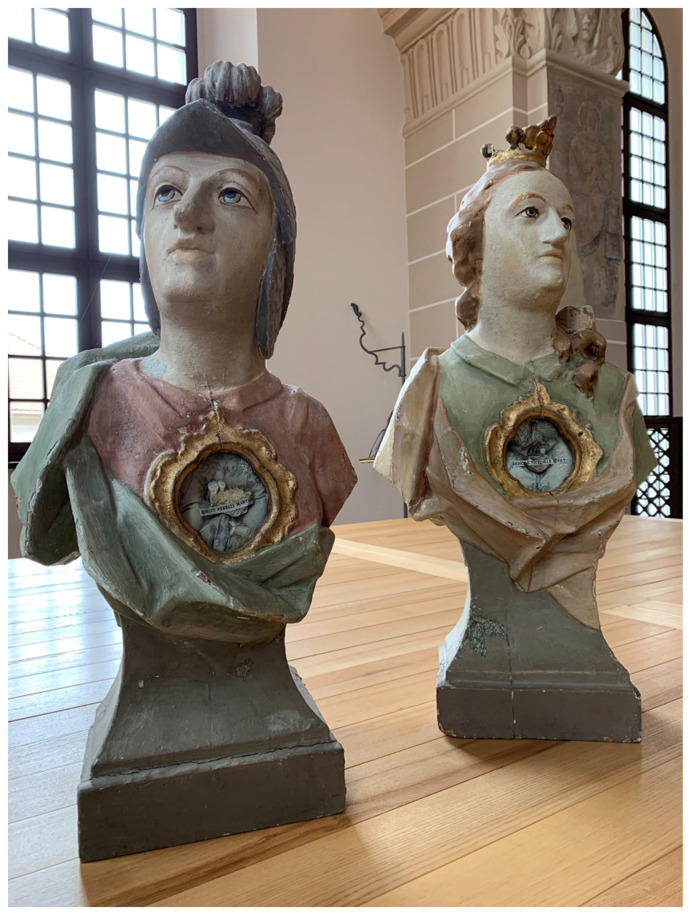
Catacomb reliquary busts of St. Aurelius (left) and St. Victoria (right), frontal view. Human remains (skeletal fragments), polychromed wood, textile, and mixed materials. Second half of the eighteenth century. Formerly Franciscan convent in Valkininkai. Church Heritage Museum, Vilnius, Lithuania. Image: Ruth Sargent Noyes.

While large insigne relics were the most sought-after, the widespread demand for and seemingly interminable supply of these paleochristian saints and martyrs were so great that “catacomb relics” actually ran a wide morphological gamut: partial remains might be installed in wooden polychrome sculptures or relic busts, such as those made around the mid-eighteenth century for the Franciscan convent in Valkininkai (Pol. Olkieniki), about 55 km southwest from Vilnius ^37^ ( Figure 3– Figure 5). Fragments could also be displayed in so-called “relic galleries” on the altar *mensa*, such as those surviving in the parish church of Viļāni (Pol. Wielony), formerly a Bernardine monastery church, today in eastern Latvia ^38^ ( Figure 6). This morphology emerged by the mid-eighteenth century to accommodate a portable solution to a dual “demand-and-supply” situation: the widespread spoliation of relics from altars, and the burgeoning quantity of miniscule relics issuing from the catacombs to satisfy burgeoning demand ^39^. Yet another solution to the problems of friable relics that proliferated in the eighteenth century were so-called *pasta di reliquie* (“relic paste”) objects, manufactured by mixing pulverized bones into a paste and molding this substance into miniature sculptures, as in two examples installed in two reliquary busts, also originally from Valkininkai, which combined these molded objects with small catacomb relic fragments ^40^ ( Figure 7– Figure 8).

**Figure 4.**
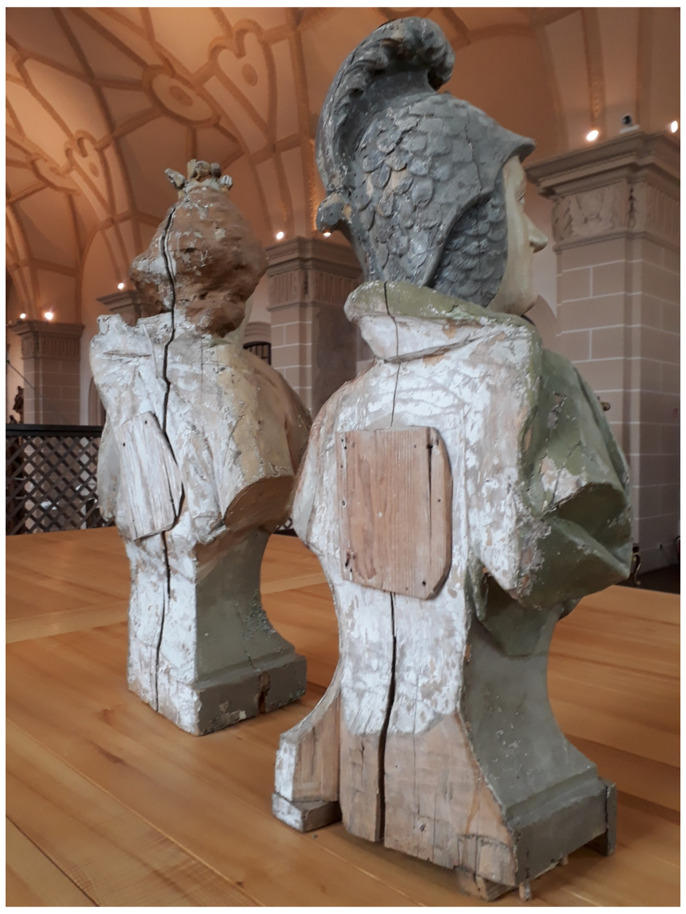
Catacomb reliquary busts of St. Aurelius (right) and St. Victoria (left), dorsal view. Human remains (skeletal fragments), polychromed wood, textile, and mixed materials. Second half of the eighteenth century. Formerly Franciscan convent in Valkininkai. Church Heritage Museum, Vilnius, Lithuania. Image: Ruth Sargent Noyes.

**Figure 5.**
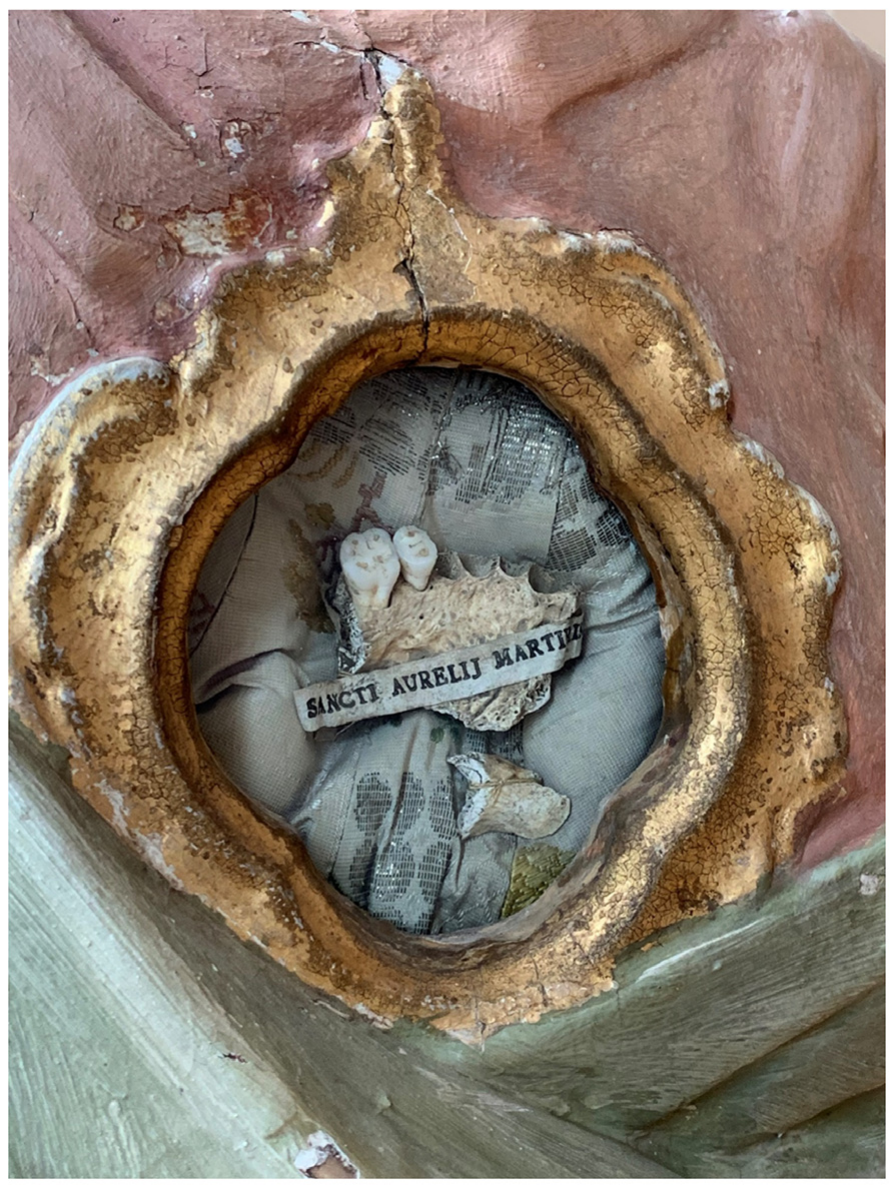
Catacomb reliquary bust of St. Aurelius, detail. Human remains (skeletal fragments), polychromed wood, textile, and mixed materials. Second half of the eighteenth century. Formerly Franciscan convent in Valkininkai. Church Heritage Museum, Vilnius, Lithuania. Image: Ruth Sargent Noyes.

**Figure 6.**
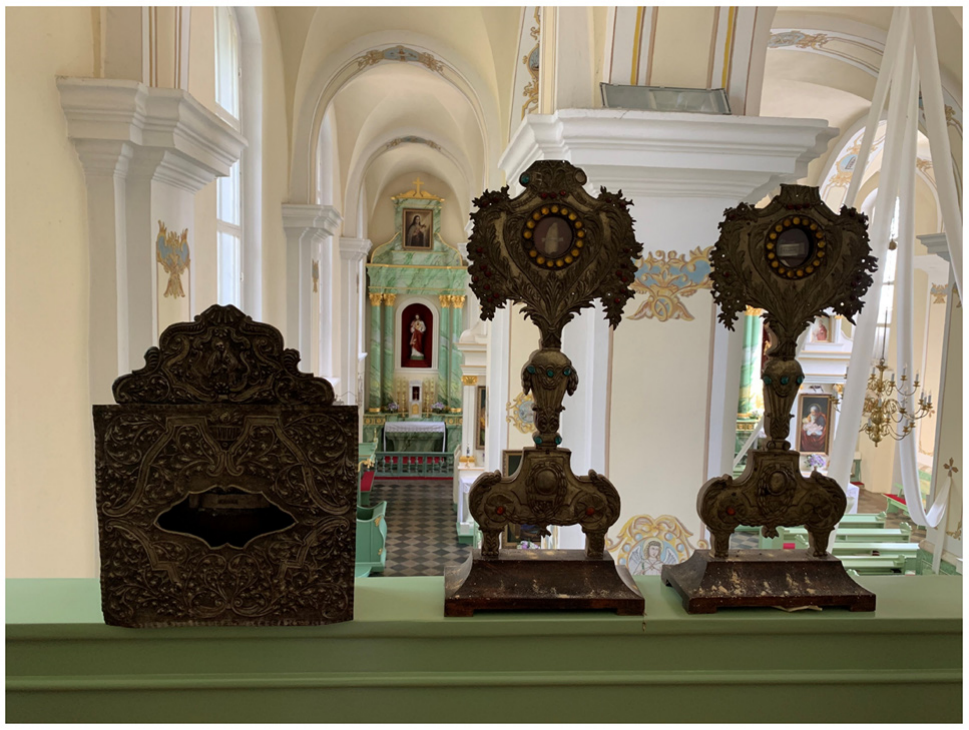
Catacomb relic galleries. Mid- to late-eighteenth century. Human remains (skeletal fragments), wood, metal, and mixed materials. Mid-eighteenth century. Parish church, Viļāni, Latvia. Image: Ruth Sargent Noyes.

**Figure 7.**
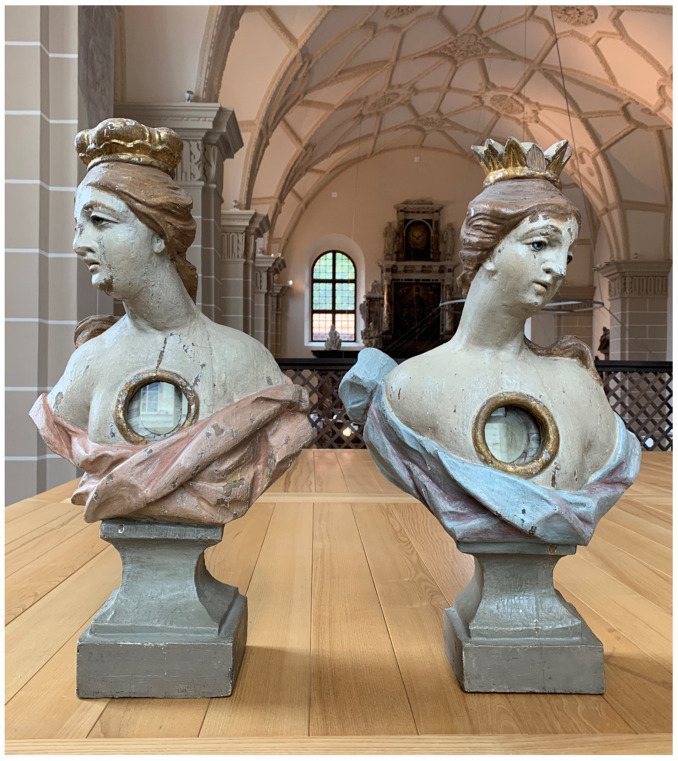
Reliquary busts containing *pasta di reliquie* and catacomb relics, frontal view. Human remains (skeletal fragments), polychromed wood, wax, textile, and mixed materials. Second half of the eighteenth century ( *post* 1765). Formerly Franciscan convent in Valkininkai. Church Heritage Museum, Vilnius, Lithuania. Image: Ruth Sargent Noyes.

**Figure 8.**
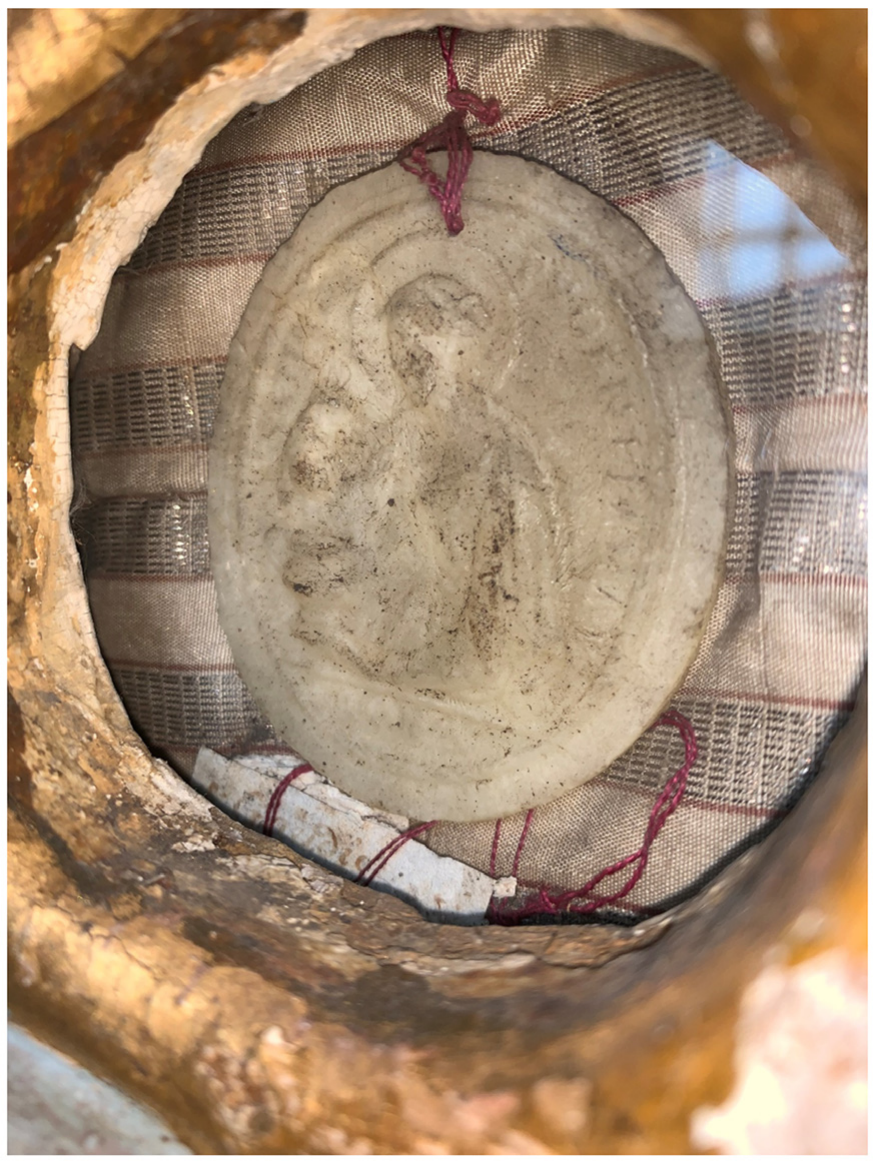
Reliquary busts containing *pasta di reliquie* and catacomb relics, detail. Human remains (skeletal fragments), polychromed wood, wax, textile, and mixed materials. Second half of the eighteenth century ( *post* 1765). Formerly Franciscan convent in Valkininkai. Church Heritage Museum, Vilnius, Lithuania. Image: Ruth Sargent Noyes.

By the time Borch found himself in the *urbe*, there was already a history of traffic in numinous catacomb imports more generally between Italian lands, the Crown, Livonia, and the Grand Duchy that stretched back well over a century ^41^. As early as the first decades of the 1600s, Jesuits divided between the Provinces of Poland and Lithuania around 80 catacomb bodies imported from Rome; after 1631 some of these relics were translated into three Jesuit churches, a private chapel, and the cathedral in Vilnius. Other catacomb relics were installed in the chapel (and subsequently to the parish church and Jesuit church) of the new Jesuit college of Kražiai (Pol. Kroże) in the region of Samogitia (Pol. Żmudź, Lith. Žemaitija); those of saints Crescentius, Clemens, Martinianus, Martiniana, Emiliana, and Faustina were brought to Samogitia *ante* 1639. In the north-eastern Lithuanian diocese of Smolensk (today in Russia) the body of catacomb saint Callistratus was donated to Smolensk cathedral church circa 1645, under the aegis of the powerful magnate and Great Standard-Bearer of Lithuania, Nicolas (Mikołaj) Sapieha (1581–1644), who also brought four such bodies to his residence in Kodeń (in contemporary eastern Poland) in the 1630s after a trip to Rome. Around 1751 the body of the catacomb saint Fortunatus was brought to Samogitia cathedral in Varniai (Pol. Wornie) ^42^. Relics of St. Valens extracted from the catacombs of Trasone and Saturnino in 1763 were translated on the occasion of the installation in the Trinitarian Church in Vilnius in 1765 under Vilnius Bishop Ignacy Jakub Massalski ^43^.

## “an unexhaustable mine of relics”

Highly desirable to Catholic religious communities, affluent grand tourists, and pious elites, catacomb relics were initially excavated by and dispersed through the networks of newly-founded religious orders like the Jesuits and Oratorians ^44^. After initial piece-meal attempts at regulation, by the second half of the seventeenth century the Apostolic Chamber had established an administrative system to centralize, legalize, and standardize the booming market for distribution of this precious resource according to requests from the faithful, particularly for the most valuable *insigne* relics comprising more or less intact skeletons ^45^. These could sell on the open market for up to 100 *scudi*, approximately 10% of Borch’s annual Grand Tour allowance. Excavating the catacombs as a numinous quarry, the Vatican oversaw exploitation of a resource it claimed on the one hand limitless, based on estimates for the total number of Roman martyrs around 64 million, and on the other under heretical threat ^46^. To put the phenomenon of catacomb relic regulation in statistical perspective, for the period 1657–1791, documented cases of their legal authentication (on which more below) totaled over thirty-five thousand; for the period 1814–1850 such authentications reached two thousand ^47^. While the phenomenon of the *translatio* of catacomb relics from subterranean Rome to Baltic Europe is admittedly little-known today, what might be described as a sort of invasion of the north by the remains of ancient Italo-Roman bodies and body parts, what might be termed the Baltic’s ‘Romanization’ or even “catacombization,” unfolded via constituencies, initiatives and dynamics both internal and external. Starting after 1600 and climaxing by c. 1800, this mass migration operated according to standards and regulations under the aegis of the Apostolic Chamber, but at the behest or demand of requesting constituencies like the Count Michał Jan Borch. Thus each exported relic represented a *response* to a particular *call*, by means of a bureaucratic system divided between two Church offices charged by 1700 with distribution: the first, the Papal Sacristan, reported directly to the pontiff; the second, the Cardinal-Vicar of Rome, superintended his Vicegerent, and in turn a designated Custodian of Sacred Relics and Cemeteries ^48^.

Borch’s autograph appeal in Italian (he was fluent in Italian, Polish, and French, conversant in German, with a basic knowledge of English) to the Custodian of the Cardinal Vicar survives bound in a volume with hundreds of other mostly successful petitions—giving some indication of the scope of the system—and shows added notations by the Cardinal Vicar’s staffage (on which more shortly) according to bureaucratic convention ^49^ ( Figure 1). Borch’s case demonstrates that such petitions characteristically represented a bureaucratic endpoint of sorts rather than point of departure, as he reminded the Custodian of a previous audience with Pope Pius VI (Giovanni Angelo Braschi, 1717–99; r. 1775–99), where the pontiff promised the holy body of a warrior-martyr ^50^. Consumer competition was keen, and the process to obtain such rare relics could entail years and multiple papal audiences, which in addition to particular linguistic and formal economies also subsumed a specific documentary and monetary system, typically comprising papal rescripts, taxes, fines, and fees for secretaries, translators, notaries, and other third-party agents ^51^. Both Sacristan and Vicar oversaw squads of so-called *cavatori* (“quarry-men”) who descended with a designated ecclesiastical expert to identify and remove authentic remains once a given supplication was approved ^52^. In Borch’s case, annotations in a second hand on the verso of his request specify that the day prior to his officially soliciting the Vicegerent, orders had been conveyed on 29 April 1778 on behalf of Pius VI that “the body of St. Victor Martyr” be given “to His Excellency the Count de Borch, one of the magistrates of Poland…such that the Catholic Faith and the cult of Saints might be honored in Poland” ^53^ (see extended data, Appendix 1). An inscription on his petition in a third hand dated 30 April specified that “the Holy Body of St. Victor [extracted] from the Cemetery of St. Priscilla,” indicating a site on the Via Salaria used for Christian burials from the late second through the fourth century ^54^.

From these inscriptions can be inferred firstly that his relics had been “baptized” with a name and identity chosen by Borch himself, most likely according to mentions in the *Roman Martyrology*, of which there were about three dozen ^55^. This was according to period custom issuing from the fact that cases of so-called *nomine proprio* catacomb relics, where traces of a name were found in situ in the burial *loculus*, were exceedingly rare, and in the majority of cases of unidentified remains, the requesting party could christen the bones themselves ^56^. Second, it suggests that Borch had either misrepresented his office (he was not yet a magistrate) or it was misconstrued by Vatican officials. Third, it demonstrates that Borch’s successful navigation of the Roman courts and the support of elites within this milieu was likely key to his success in being granted relics at all (and apparently on short notice). Lastly, it indicates that despite the recent partition of Poland-Lithuania and Russian annexation of his family’s latifundia in Polish Livonia, Borch was still perceived—and presented himself—in the papal city as a relic of the Polish aristocracy and defender of Catholicism, and that these perceptions may have helped his case in procuring relics ^57^. In fact, the young nobleman’s personal correspondence from this period shows he was sometimes given to exaggerating his official titles at the Italian courts, and was particularly sensitive regarding what he viewed as disrespectful treatment at the papal court, where the rescheduling of his audience with Pius VI was a particular sore point ^58^.

Borch had been in close contact for two years with high-ranking members of the Curia who both had connections to Catholic interests in Poland-Lithuania and to the cult of relics: Cardinal Giovanni Francesco Albani (1720–1803), who was relator of the Sacred Congregation of Indulgences and Sacred Relics and Cardinal Protector of the Kingdom of Poland in the Roman Curia, and Cardinal Antonio Eugenio Visconti (1713–88), who performed various duties on the Congregation for Indulgences and Sacred Relics (Prelate, 1742-, Prefect, 1782-) and had been nuncio to Poland (1760–66) ^59^. A mounting existential crisis for the Holy See against the expanding Russian imperial remit crystalized during the years that Borch spent in the orbit of the Roman courts ^60^. Following the first partition of Poland in 1772, Russian Empress Catherine unilaterally founded the Latin Catholic Diocese of Mohilev (Pol. Mohylew) in defiance of the laws of the Catholic Church and in direct challenge to the pope, splitting its territory from the Dioceses of Inflanty, Vilnius and Smolensk, and appointing Stanisław Bohusz Siestrzeńcewicz (1731–1826) as its first bishop in 1773. From Rome’s perspective Siestrzeńcewicz was a controversial candidate, given that he was a Catholic convert only recently ordained. In 1782 the tsarina elevated Siestrzeńcewicz to metropolitan archbishop of Mohilev ^61^. Pius VI initially refused to recognize this new see and Catherine’s appointee, then initiated negotiations represented by nuncio Giovanni Andrea Archetti ^62^. Eventually, facing the prospect of the founding of a new Russian papacy, Pius canonically sanctioned the new Archdiocese of Mohilev with the Bull "Onerosa pastoralis officii" of 15 April, 1783, which reserved to his office the foundation of other dioceses in the territory of the archdiocese, the largest in the world at the time ^63^. The result, however, left a Russian Catholic archdiocese typical in structure but exceptional in that it reported not to the Holy See but to tsarist government ministry; Archetti was not recognized as an official nuncio and a Russian “envoy” was only established in Rome in 1817 ^64^.

**Figure 9.**
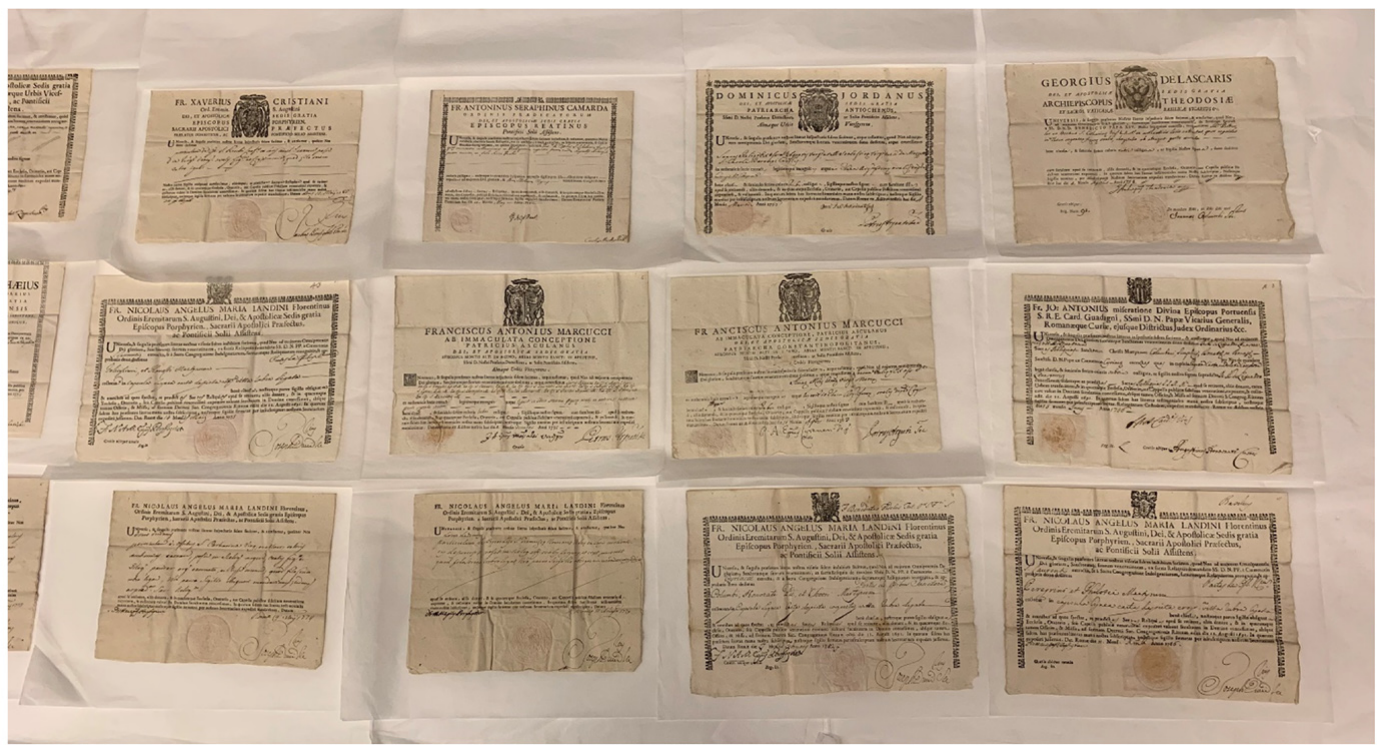
Authentic certificates. Seventeenth-nineteenth centuries. Ink on laid rag paper. Church Heritage Museum, Vilnius, Lithuania. Image: Ruth Sargent Noyes.

**Figure 10.**
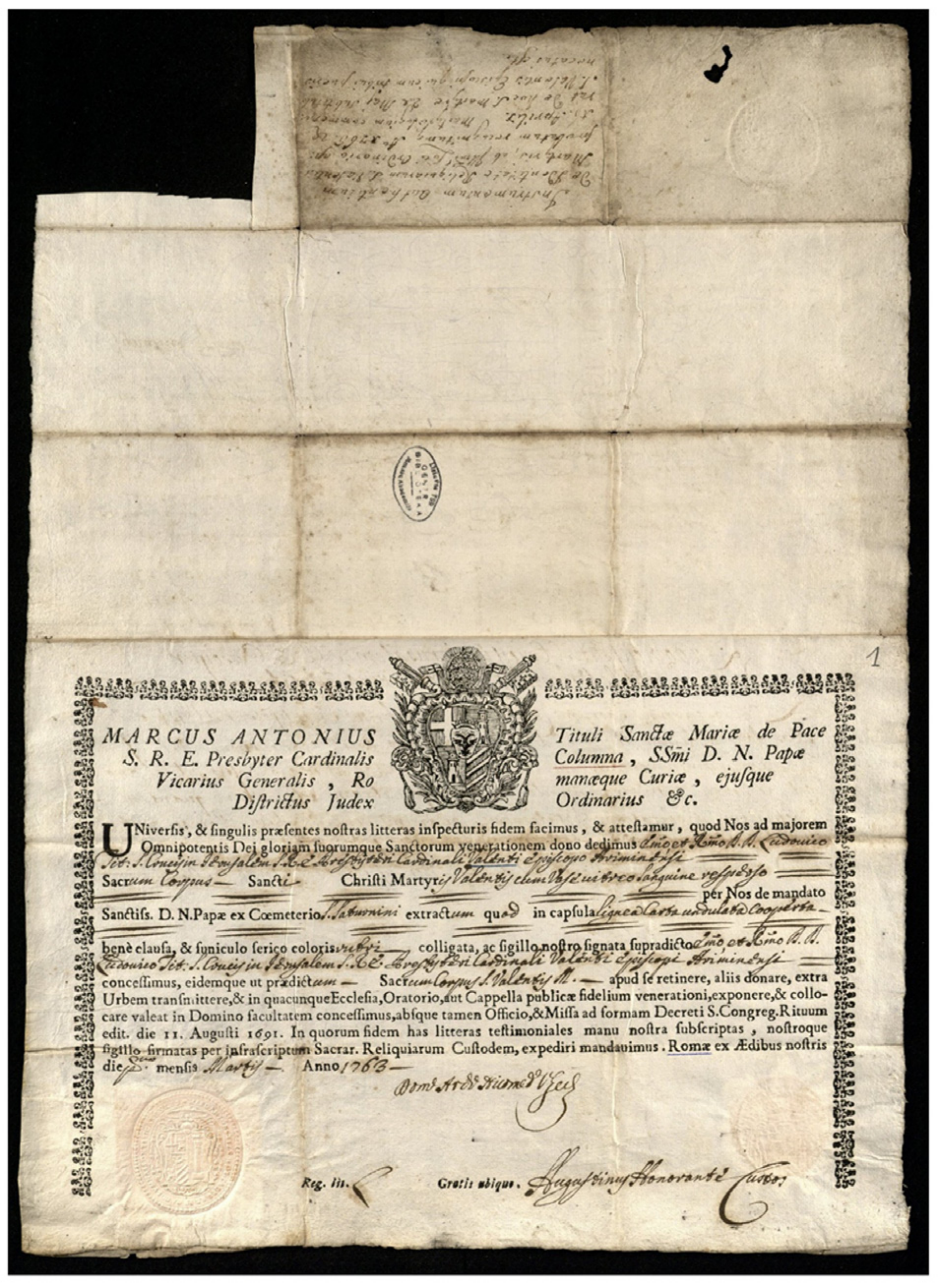
Authentic for catacomb relics of St. Valens from Catacomb of St. Saturninus, issued by the Custodian of Relics and Cemeteries on behalf of the Vicar-General. Ink on laid rag paper. 1763. Vilnius, Wroblewskis Library of the Lithuanian Academy of Sciences Archives, LMAVB_RS F273-388 1r-2v. This figure has been reproduced with permission from: Wroblewskis Library of the Lithuanian Academy of Sciences Archives.

That Borch was swiftly conceded prestigious relics of a supposed Rome soldier martyred for the true faith should be viewed against the unfolding antagonism between encroaching Russification in his homeland and the efforts of the Holy See to sustain its legacy of global hegemony. Victor’s bones may have been autopsied by Church-appointed medical experts for signs of violent injury to substantiate a martyr’s death, though such paleopathology was not carried out in every case ^65^. To ratify their genuineness and ensure the chain of custody, Victor’s relics were furnished with a so-called Authentic: these pre-printed notarized certificates baptized the sacred remnants with names and identities that as noted above were usually indicated by the supplicant ^66^ ( Figure 9). As can be seen from a period example for catacomb relics of St. Valens from the Catacomb of St. Saturninus, issued by the Custodian of Relics and Cemeteries on behalf of the Vicar-General in 1763, Authentics also furnished a validating description of the relics and the container into which they were placed, and specified from which cemetery the remains were taken and to whom they were entrusted ^67^ ( Figure 10).

Luigi Francesco Leonardo Desanctis (1808–1869), an Italian-born Catholic theologian turned evangelical protestant and ardent Catholic critic, penned an eyewitness exposé of the processing of catacomb relics in an account of his shocking visit to the former Jesuit College in Rome, partly repurposed as a relic depository:

“…The second room is full of wooden chests, dyed green, containing the relics of popular saints. In this room there are four priests, whose business it is to place the relics in shrines for distribution, and you shudder to see the tables covered with a confused mass of bones, teeth, pieces of old garments, hair, and so forth, tumbled together with the greatest indecency, so that I could not believe that they were relics, till our priest assured me of the fact. I conclude the priests who distribute them do not believe in them; if they did, they would treat them less contemptuously….[The Reverend Custodian] said ‘The catacombs send us each week the bodies of saints, such that we have an overflow of common relics. Our catacombs are an unexhaustable mine of relics, but we have really very few distinguished relics, and don’t know what we’ll do in 50 years’ time.’ I asked how the Pope could decide whether the skeletons found in the catacombs belonged to the saints. ‘The Pope!’ he replied, ‘The Pope doesn’t get mixed up in such things. He commits them to the Cardinal-Vicar, the Vicegerent, and the Sacristan…who visits the remains as they are disinterred, and when he believes them to have been saints, sends them here for us to baptize and distribute to the faithful’. ‘To baptize!’ I interrupted, with astonishment; ‘do you baptize dead bones?’ The Custodian explained that by baptizing, he meant nothing more than naming them. No one knows to whom a certain skeleton once belonged, but the reliquary stands in need of relics of S. Pancrazio, for example, and the skeleton is called S. Pancrazio.” ^68^

## “miraculous bones, with unqestionable signs of martyrdom”

Desanctis’s colorful description gives a sense of the sordid banality of the catacomb relic boom, a banality that unfolded from the dark and dank *loculi* of the catacombs mined by professional quarrymen to the ersatz workshops in the houses of religious orders where bones and other remnants were sorted and christened, and a banality camouflaged not only by the intricate courtly rituals, conventions and procedures that unfolded from the palaces of cardinals who facilitated supplicants like Borch to the Vatican offices and lavish rooms of the papal palace, but also by the material enclosing of the relic-remains within a sealed *theca* (small glass capsule) for miniscule relics or *baule* (chest) for larger remains, in which these relics were ordinarily delivered to supplicants ^69^. These typical containers accomplished in an economical fashion essential reliquary functions, instantiating the ritual processes of selection, enshrinement, and institutional authentication that imbued the contents with beauty, value and efficacy, and staging their advent to the audience of the faithful, even if this appearance actually entailed the contents’ invisibility ^70^. Once the relics arrived at their final destination, local communities often had them translated into more elaborate enclosures and displays, a widespread phenomenon most spectacularly realized in the reassembled, bedecked, and bejeweled skeletons installed at the Bavarian collegiate basilica of Waldsassen from the late seventeenth century ^71^.

By the late eighteenth century when Borch received St. Victor, however, a confluence of factors in Rome had given rise to a new satellite industry that sprung up around the robust trade in catacomb relics, in part as a solution to the Custodian’s complaint as relayed by Desanctis: namely, the mounting dilemma that while global demand for relics remained strong, the catacombs offered an “unexhaustable mine” for countless “common” but increasingly scarce “distinguished” relics, a state of affairs that threatened to put the entire economy of catacomb relic translation in jeopardy ^72^. By the turn of the nineteenth century, prestigious *insigne* remains—intact major bones, integral skulls, and especially complete skeletons—were in increasingly short supply, as the seemingly endless resource of the *urbe*’s underground ossuaries had been overexploited and largely reduced to small, fragile, friable fragments, which lacked the spiritual (and social) caché associated with large, intact, and recognizably corporal integral relics ^73^. This crisis in numinous resource management intersected with a constellation of others during the final pontificates of the century, that of Clement XIV (Giovanni Vincenzo Antonio Ganganelli, 1705–74; r. 1769–74) and Pius VI (Giovanni Angelo Braschi, 1717–99; r. 1775–99), perhaps best remembered for presiding over the dissolution of the Jesuit Order, of the territory of the Papal States, of the papacy’s financial resources, and of Catholic hegemony more generally ^74^.

Facing a centuries-long Papal dominion under threat as never before, under their purview the Holy See mounted an Enlightenment counter-reform campaign to reaffirm the papal city as *caput mundi*, renovate the image of the papacy as international arbiter of taste, reaffirm the illusion of integral Catholic empire, and refill depleted Vatican coffers ^75^. Crucial to these manifold endeavors was the promotion of Rome as a primary destination on the Grand Tour, attracting flocks of foreigners as pilgrims to marvel at not only pious Christian sites and the opulent papal court, but also the remarkable antiquities that had endowed fame on the city for centuries and were concurrently collected, showcased, and safeguarded in new buildings and galleries overseen by the pontiff, his extended family and courtly circles, as well as sold in the form of originals, reproductions, and forgeries to well-off tourists like Count Borch ^76^.

**Figure 11.**
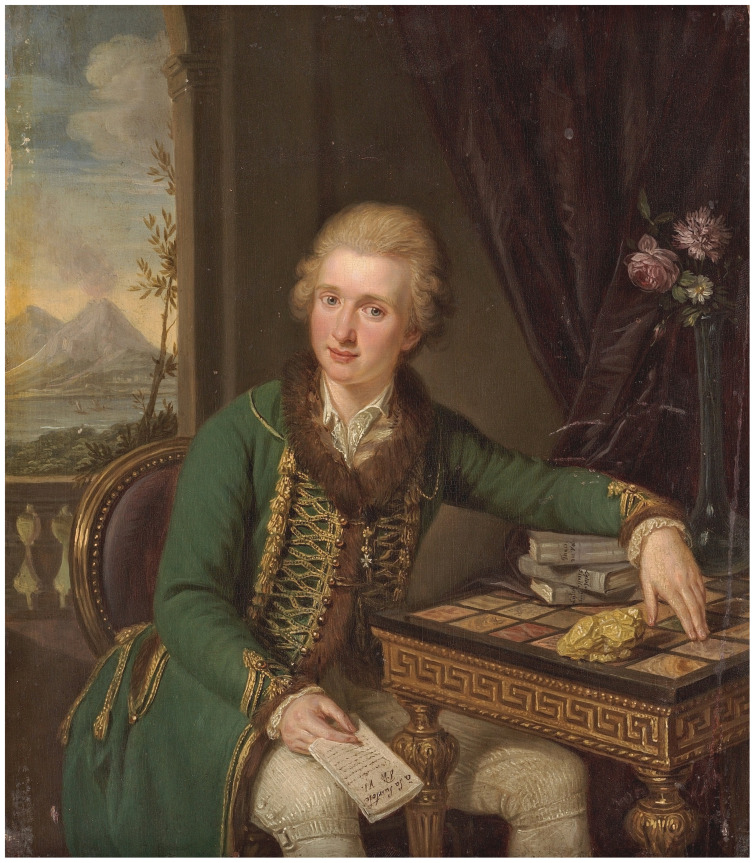
Ludwig Guttenbrunn (1750–1819), Portrait of Michał Jan Borch. Oil on wood panel. Circa 1778. Tretyakov Gallery, Moscow, Russia. Image in the public domain: https://www.tretyakovgallery.ru/en/collection/portret-grafa-mikhaila-ioganna-fon-der-borkha/ (accessed 15 February 2021).

For his part, while in Rome Borch longed to sit for sought-after portraitist Pompeo Battoni and hankered for cameos by renowned glyptic artist Giovanni Pichler; his predilections as an aspirational collector were documented in an oil portrait from this period by Austrian-born artist Ludwig Guttenbrunn (1750–1819), today in the Tretyakov Gallery, Moscow ( Figure 11). The painting figures him against a Neapolitan landscape, seated at an ornate *Scagliola* or *Pietre Dure* table, signaling both his good taste ( *Scagliola* was equally popular in Italy and the Grand Duchy) and mineralogical and antiquarian interests ^77^. Gesturing to his own volumes on natural history and ethnography in Italian lands that he published during his sojourn on the peninsula, he proffers a folio with the inscription “to His Holiness Pius VI,” adumbrating at once the papal audience where Borch solicited a promise of relics from the pontiff, and the dedication to Braschi of his *Lythologie sicilienne* ( *Sicilian Lythology*), a work on petrology and mineralogy, which received its imprimatur from the Vatican Master of the Sacred palace on 9 June 1778, shortly after Borch received his relics ^78^. His dedication framed his own natural historical expertise against Pius’s many initiatives and strategies to systematize, valorize, and extract profit from resources under the papal aegis ^79^, most famously in the *Bonificazione Pontina* (“Pontine Land Reclamation”) to develop and cultivate the malarial Pontine Marshes south of Rome ^80^, but also to drain marshes around Città della Pieve, Perugia, Spoleto and Trevi and connect lake Trasimeno near Perugia to the Tiber river ^81^, open lead and aluminum mines ^82^ and marble and alabaster quarries ^83^, boost tobacco and paper production ^84^, reform the *Annona Pontificia* regulating grain in papal territories ^85^, and monetize water flowing into Rome ^86^.

**Figure 12.**
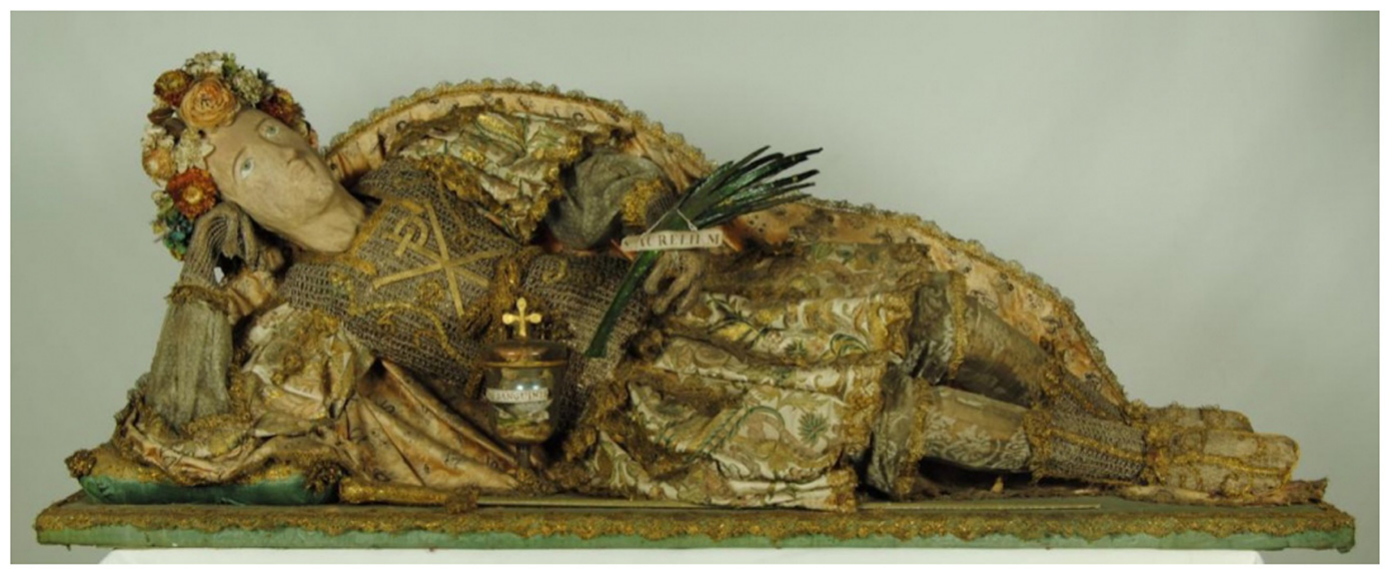
*Corposanto* relic-sculpture of St. Aurelius, frontal view. Human remains (skeletal fragments), metal wire, wood, wax, textile, polychromy, laid paper, and mixed materials. Circa 1789. Cathedral of Porto, Portugal. This figure has been reproduced with permission from: Joana do Carmo Palmeirão, “Imagem-relicário de Santo Aurélio mártir pertencente à Sé Catedral do Porto. Estudo e conservação integrada das relíquias,” MA thesis, Universidade Católica Portuguesa, 2015, 153, fig.1.

Amongst this range of environmental resources might also be posited the numinous remains conserved within the *loculi* of the catacombs. The entwined issues of dwindling supply and strong demand created an opportunity for innovation: *corpisanti* catacomb relic-sculptures represented a solution that interwove patronage, collecting and production in the arts, spiritual, bureaucratic and economic reforms, and resource management. *Corpisanti* bring together the holy relic and the reliquary container into a single sculptural form, incorporating bodily remains in the form of scant bone fragments within a three-dimensional sculpture made to approximate a human figure, outfitted and staged to appeal both to late baroque-rococo courtly tastes and neoclassical antiquarian sensibilities ( Figure 2, Figure 12). The excavation, production, and distribution of *corpisanti* entailed strip-mining the already over-exploited Roman catacombs for friable skeletal fragments and consolidating the often pulverized relics within a luxurious life-size material fantasy of integral anthropomorphy. Scholarship tends to posit 1772 as the *terminus post quem* for the advent of these relic-sculptures in the Roman milieu, attributing the first exemplar to the administration of surgeon and would-be Roman courtier Antonio Magnani. He oversaw in 1772 the reconstituted catacomb relics of St. Felicissima, displayed in the Palazzo Ruspoli to great popular acclaim, and thereafter under the aegis of the Papal Sacristan superintended the burgeoning *corpisanti* industry on the papacy’s behalf in a specially created Vatican post, “Restorer of Holy Bodies of the Pontifical Chapel,” which vanished with his death in 1808 ^87^. However, it will be shown here that relic-sculptures surviving in the Baltic suggest that Magnani's role was that of consolidating (not initiating) a phenomenon that began by the middle of the eighteenth century and climaxed during the Braschi pontificate. Their serial manufacture by nameless artisans in workshops under the Vatican’s purview started c. 1750 and reached a kind of apogee under Pius VI, proliferating exponentially and achieving such a morphological proto-industrial consistency that some specimens appear almost identical. Scholarship also widely refers to these unique objects as *ceroplastic* relics, since some examples (possibly the earliest specimens, though this remains to be verified) included visages, hands, or other body parts modeled from wax ^88^. However, that most surviving specimens include very minimal wax in their fabrication renders this nomenclature somewhat imprecise and suggests that wax was phased out of production—possibly because of the expense of high-quality material, because it demanded highly skilled artisans and was less conducive to large-scale production, or because it tended to degrade (discolor, melt, or attract hungry church mice) ^89^. The nomenclature *cuerpo-relicario* (“body-reliquary”) adopted in some scholarship is more accurate ^90^.

**Figure 13.**
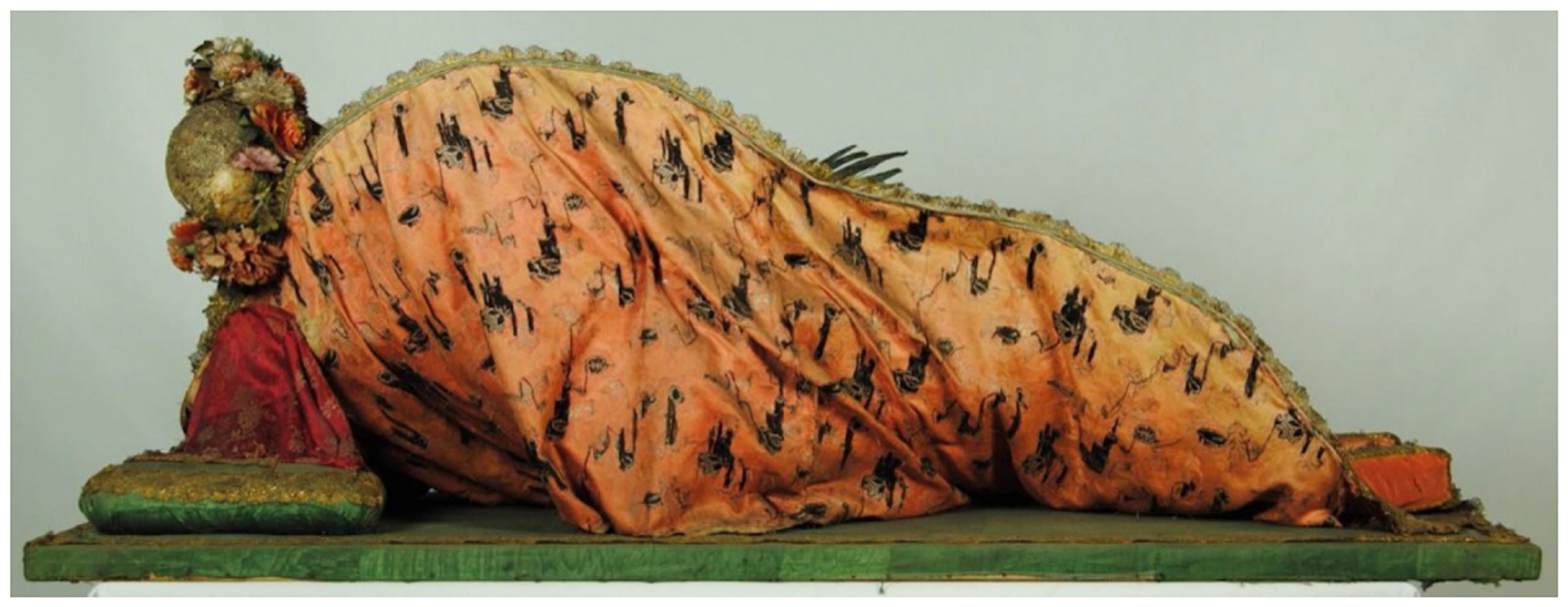
*Corposanto* relic-sculpture of St. Aurelius, dorsal view. Human remains (skeletal fragments), metal wire, wood, wax, textile, polychromy, laid paper, and mixed materials. Circa 1789. Cathedral of Porto, Portugal. This figure has been reproduced with permission from: Joana do Carmo Palmeirão, “Imagem-relicário de Santo Aurélio mártir pertencente à Sé Catedral do Porto. Estudo e conservação integrada das relíquias,” MA thesis, Universidade Católica Portuguesa, 2015, 153, fig.2.

Borch’s pursuit of geological knowledge regarding the formation and detection of subterranean resources and riches, his aspirations at the courts of Italy, his self-fashioning as an elite defender of Catholicism on the north-eastern frontier of Europe, and his ambitions as a collector of precious *naturalia*, *mineralia* and art works, made him an ideal consumer of this relatively new luxury commodity. Once he had the authenticated relics of St. Victor deposited without art, as Desanctis observed, in a wooden casket for transport, he could opt to keep the box of bones; or, he could engage additional middlemen to broker the transformation of the fragments into a *corposanto* relic-sculpture. That a good portion of period requests for *corpisanti* came from petitioners like Borch who had in fact not come to Rome with the principal aim of securing such relics, but only arrived at the idea after some time in the city, suggests aggressive advertising, even as these numinous artworks grew more difficult to obtain, thereby enhancing their perceived value as luxury goods. *Corpisanti* were of course in of themselves advertisements for a variety of Roman luxury industries concurrently fostered by the Apostolic Chamber, for which the fertile *Bonificazione Pontina* and the Apostolic Chamber’s other initiatives were to have furnished raw materials, manufactured products, and byproducts, such as wire, paper, cotton and hemp, and textiles. A recently analyzed example utilized at least eleven different textiles, eight embroidery techniques, and fifteen different kinds of metallic thread ^91^ ( Figure 12, Figure 13). Spectacular ornamentation made these figures very much at home in outré courtly environments of the period.

**Figure 14.**
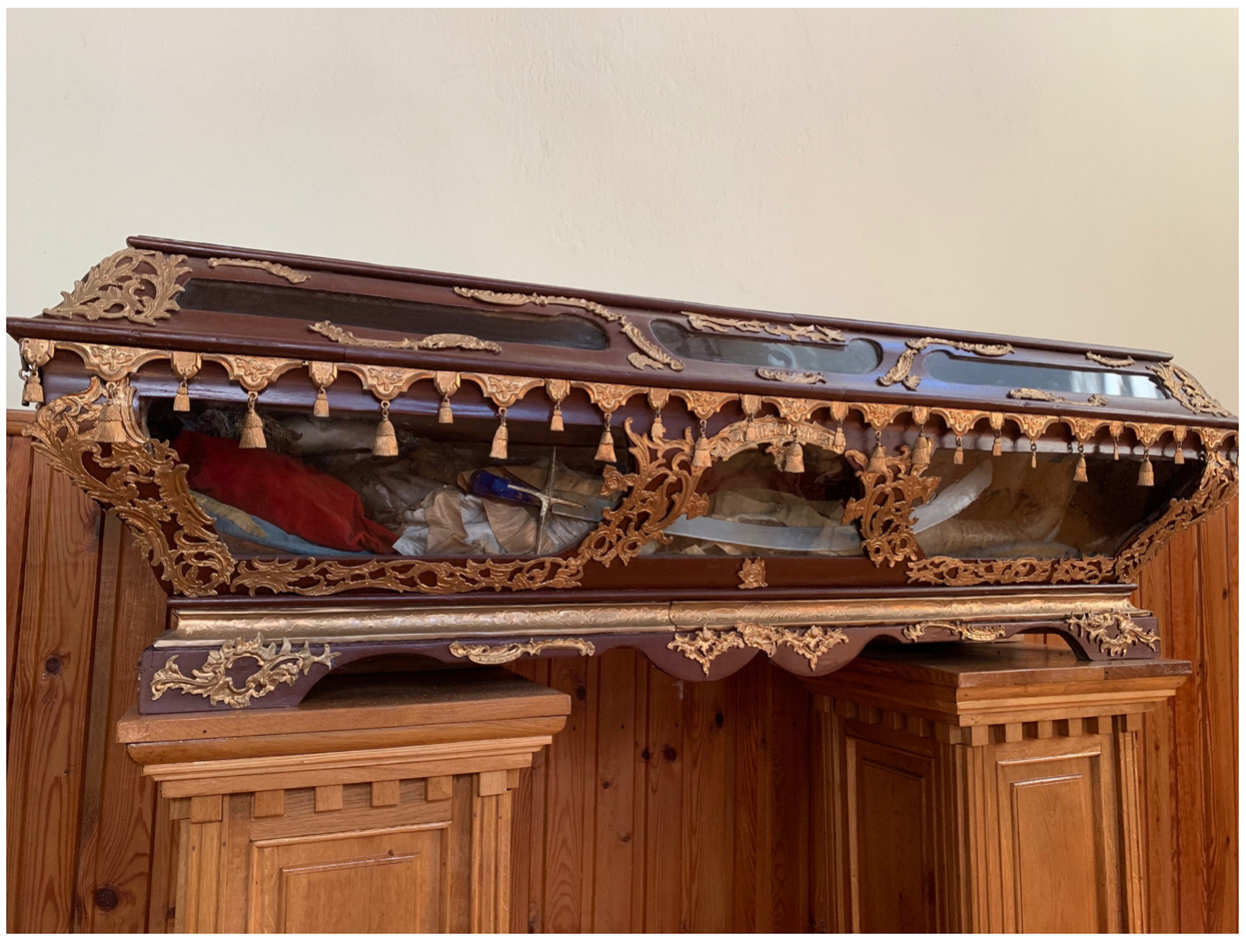
*Corposanto* relic-sculpture of St. Bonifacus. Human remains (skeletal fragments), metal wire, wood, wax, textile, polychromy, laid paper, and mixed materials. Second half of the eighteenth century ( *post* 1765), altered late nineteenth century. Formerly Franciscan convent in Valkininkai. Valkininkai Parish Church, Lithuania. Image: Ruth Sargent Noyes.

**Figure 15.**
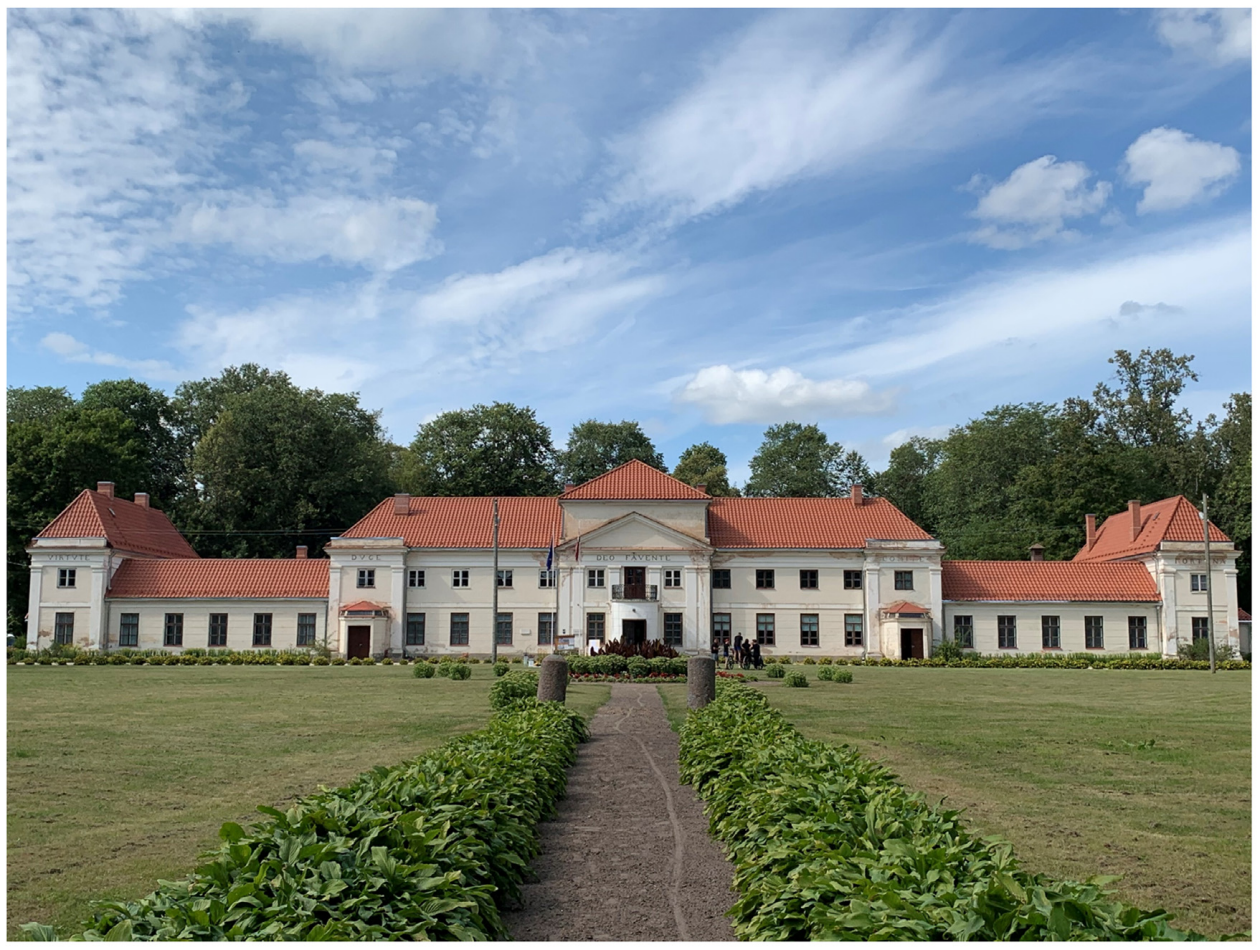
Vincenzo Mazotti, Borch manor palace. Turn of the nineteenth century, circa 1783–1810. Varakļāni, Latvia. Image: Ruth Sargent Noyes.

**Figure 16.**
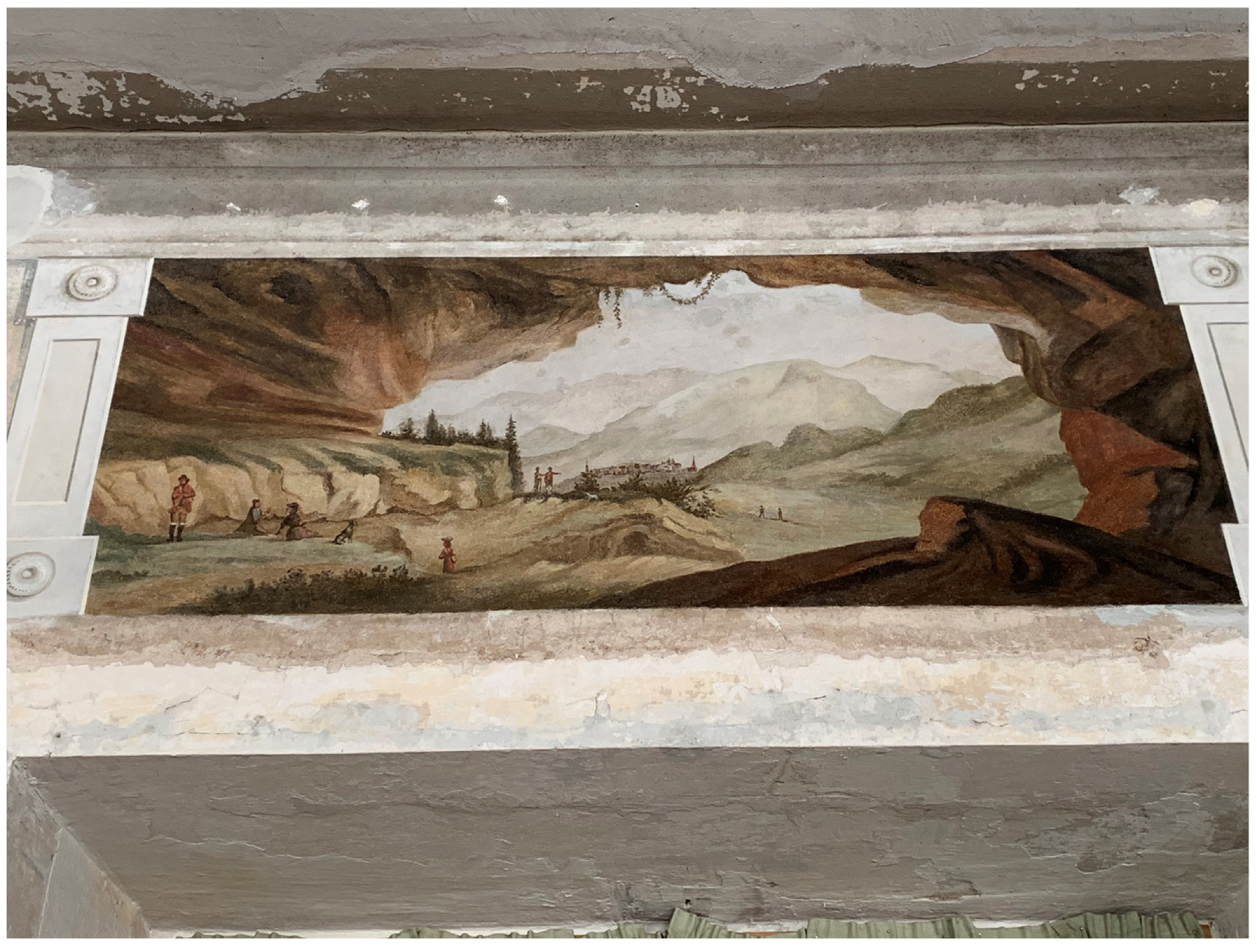
Unknown artist, mural painting of a landscape *veduta* with grotto. Turn of the nineteenth century, circa 1783–1810. Borch manor palace, Varakļāni, Latvia. This figure has been reproduced with permission from: courtesy of Varakļāni Regional Museum.

Dressed and cabinetized with an air of archaeological authenticity within a tableau setting redolent of the catacomb burial cubiculum inside a sumptuous glass coffin, *corpisanti* like Victor generally represented one of two types: the male *Miles Christi*, equipped as one of the myriad Roman soldiers martyred after converting to Christianity, and the female *Sponsa Christi*, fitted out as one of the virtuous Roman virginal converts martyred for refusing marriage to anyone but Christ ( Figure 14). Both types were typically arranged in a reclining position modeled after ancient sarcophagi, classical statuary, and Roman baroque church sculpture that evoked hagiographic accounts of execution, entombment, and ecstasy—leaning on an elbow subtly addressing the beholder, or recumbent facing heavenward ^92^. They were most often posed on a thin mattress-like upholstered platform and propped up on silk chintz or similarly elaborately covered cushions, accessorized with sandals, armor, weapons, other accoutrements, and a small chalice representative of the so-called *vas sanguinis,* an ampule of blood often found in the catacombs and identified as proof of martyrdom ^93^. When Borch eventually did return to newly Russified Livonia after his father’s death in 1780 and settled on his inherited estate of Varakļāni (Pol. Warklany), he first kept Victor in the chapel inside his Italianate palace, which he had rebuilt by Vincenzo Mazotti, a Roman of noble parentage with courtly aspirations in Warsaw and some architectural training ^94^ ( Figure 15). Borch decorated the interior of his palace with mural paintings of Italianate landscape *vedute*—featuring mountains and grottos that echoed his expertise in lithology—and classicizing trompe-l'oeil recreating palatial decoration of imperial Rome that he likely saw first-hand during his Grand Tour ^95^ ( Figure 16– Figure 18). Inside the Varakļāni palace surrounded by vestiges of the count’s Italian travels and a gallery of family portraits, the relic-sculpture disinterred from Roman earth was initially placed in proximity to natural historical cabinets displaying ample collections of minerals, stones, and gems unearthed from Italian soil, realizing the Pauline metaphor in First Peter 2:4 of the bodies of saints as precious “living stones” in luxurious textiles and bric-a-brac ^96^. In this highly suggestive context, St. Victor elicited the Borch’s origins in Livonia among pious Medieval crusaders under the papal aegis, forging a connection with the first victorious Christian martyrs of the past and devout champions of Roman Catholicism amidst religio-political and cultural upheaval in his homeland.

**Figure 17.**
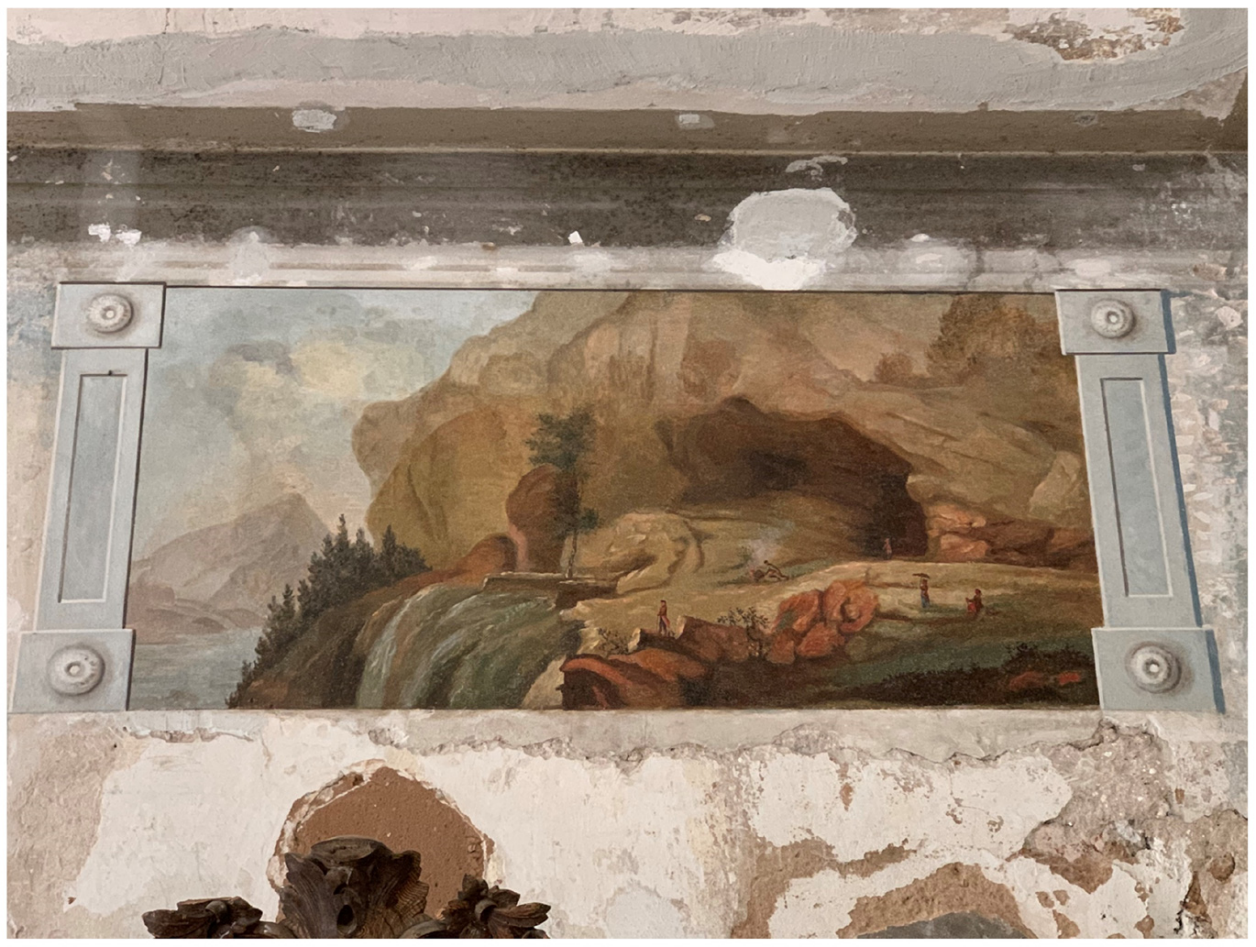
Unknown artist, mural painting of a landscape *veduta* with grotto. Turn of the nineteenth century, circa 1783–1810. Borch manor palace, Varakļāni, Latvia. Image: Ruth Sargent Noyes.

**Figure 18.**
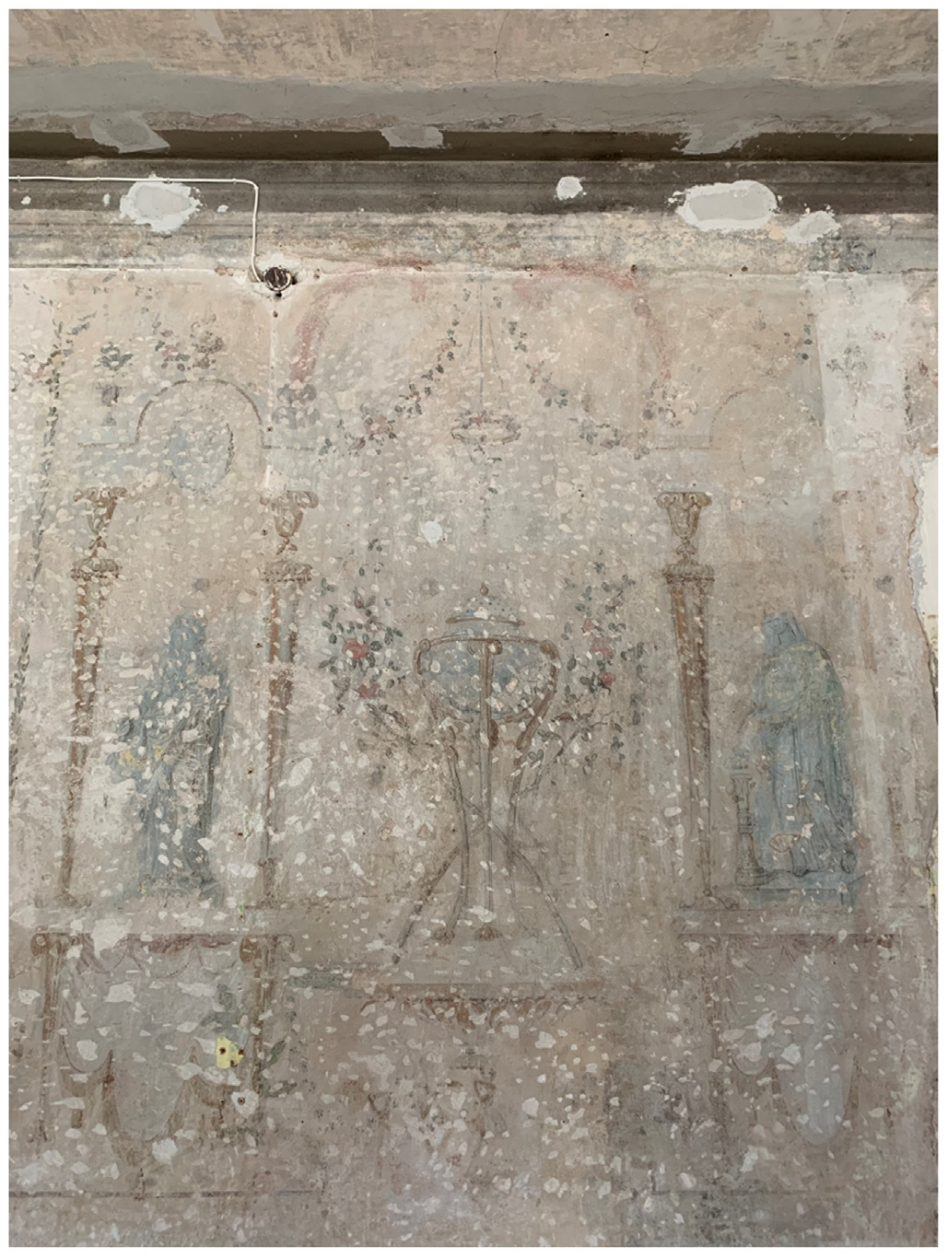
Unknown artist, trompe-l'oeil mural painting with classical statues, grotesques, festoons, and tripod. Turn of the nineteenth century, circa 1783–1810. Borch manor palace, Varakļāni, Latvia. This figure has been reproduced with permission from: courtesy of Varakļāni Regional Museum.

**Figure 19.**
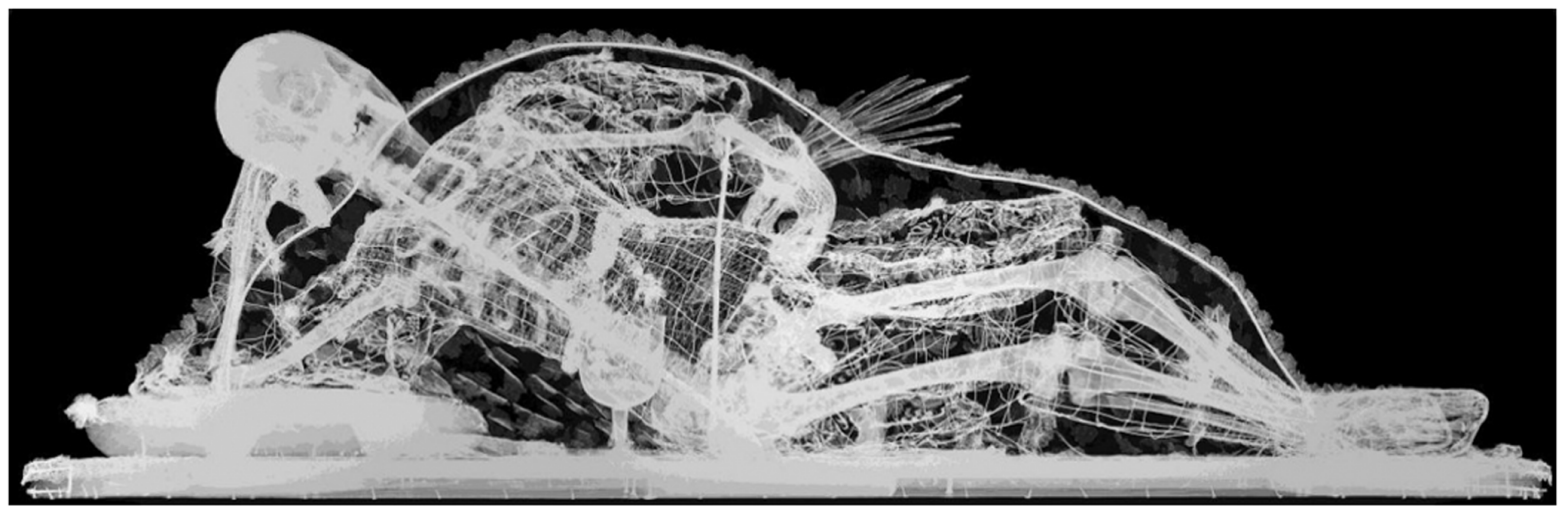
*Corposanto* relic-sculpture of St. Aurelius, radiographic view. Human remains (skeletal fragments), metal wire, wood, wax, textile, polychromy, laid paper, and mixed materials. Circa 1789. Cathedral of Porto, Portugal. This figure has been reproduced with permission from: Joana do Carmo Palmeirão, “Imagem-relicário de Santo Aurélio mártir pertencente à Sé Catedral do Porto. Estudo e conservação integrada das relíquias,” MA thesis, Universidade Católica Portuguesa, 2015, 172, fig. 48.

Both the erudite fashioning and lavish decoration of these sculptures served to mask the material banality of their manufacture: while referencing monumental sculpture in form and style and functioning as a reliquary uniquely embodying a member of the heavenly court in distinctly worldly materials, these objects were actually closer to puppets, dolls, ex-votos, pignattas, and similar ephemeral sculptures in fabrication ^97^. Recent investigations using more and less invasive methods reveal common aspects: skeletal armatures of wood and wire, flesh and limbs of *cartapesta* (compressed, molded rough-grade paper akin to papier maché) stuffed with cotton wool and mixed vegetal materials like hemp, skin of glue-saturated thin fabric polychromed with wax—all comprising a three-dimensional scaffold into which were interpolated scarce bone fragments puzzled together in defiance of actual anatomy, lavishly translated into a visual fantasy of a whole restored beatific corpse ^98^ ( Figure 19). Their scientific inaccuracy and material and socio-cultural precarity constituted the punchline to French occultist Jacques Albin Simon Collin de Plancy’s 1821 description of the sculpted relic-figure of St. Ovide, “a practically unknown holy martyr, who was the patron [saint] at one time quite famous in Paris. His body was greatly venerated by the Capuchins of place Vendôme…That which was particular about this body, was that it had two left feet, which were burnt in 1793” ^99^.

The precarious position of the figure St. Victor as a diplomatic avatar for the Roman Church amidst tense religio-political struggles in the northern borderlands is attested by the conditions of the eventual translation of the *corposanto* relic-sculpture in April 1783 from Borch’s palace to an eponymous chapel inside the parish church in the center of Varakļāni, which he was developing into a center for religion and trade ^100^ (see extended data, Appendix 2). His plan for the St. Victor chapel included an altarpiece (apparently never realized) depicting the martyr before the Roman Emperor Diocletian ^101^. The count’s translation of St. Victor and his ability to obtain a plenary indulgence for celebrating the saint’s annual festival can not only be linked to contemporary negotiations between Russian imperial and Roman papal authorities regarding the status of the Archdiocese of Mohilev to which Varakļāni was subject, but was directly predicated on Pius VI’s recognition of Mohilev in April that same year ^102^. The jumbled and heavily edited text of a draft for the dedicatory inscription for St. Victor’s eponymous chapel that survives amongst Borch family papers in the Latvian Historical Archive suggests either confusion about or resistance against Siestrzeńcewicz’s title and the precise status of the Roman Church in the region ^103^.

## “a special body of some Saint”

**Figure 20.**
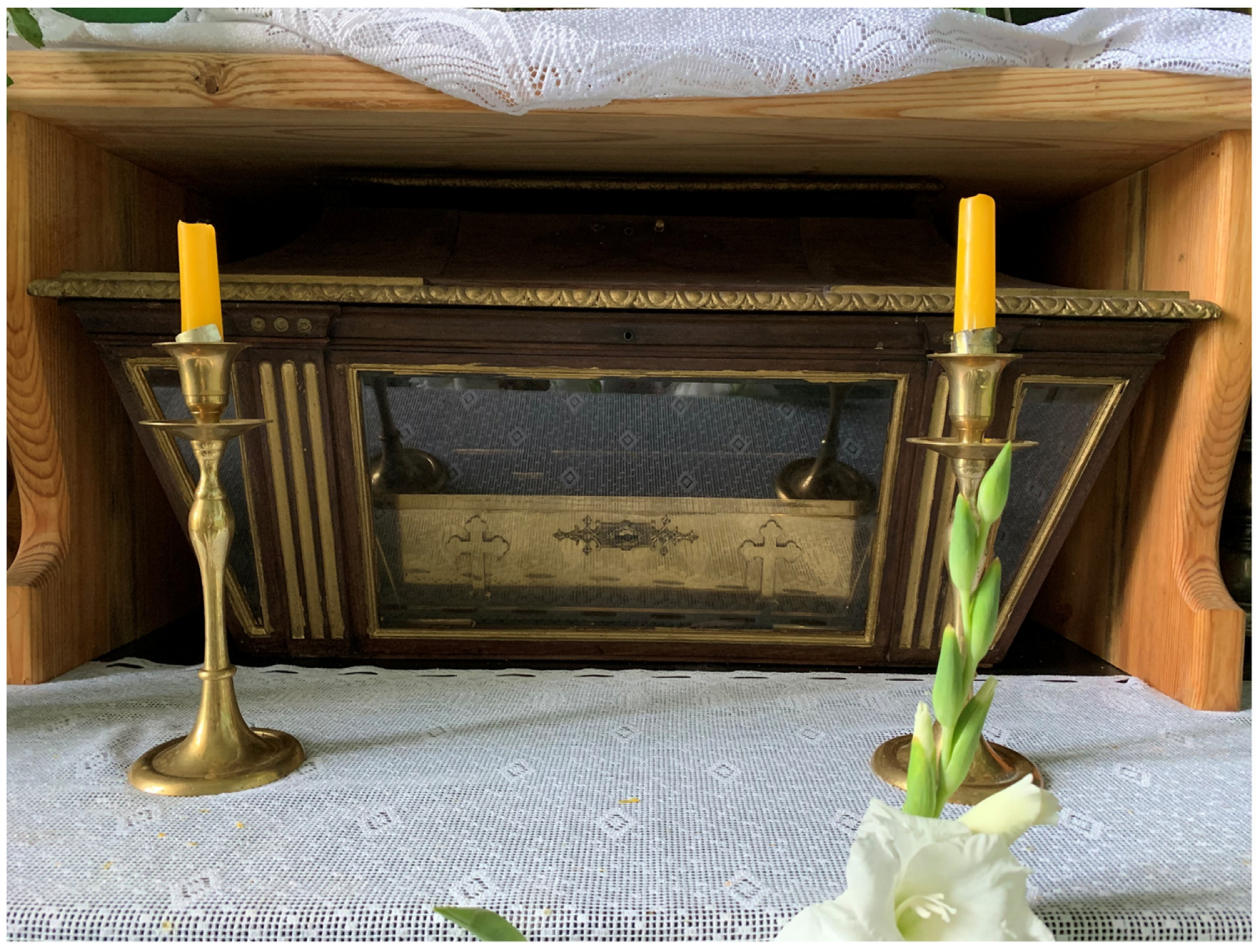
Outer reliquary coffin containing remains of St. Victor relics. Polychromed wood and glass. Early- to mid-twentieth century. Varakļāni parish church, Latvia. Image: Ruth Sargent Noyes.

**Figure 21.**
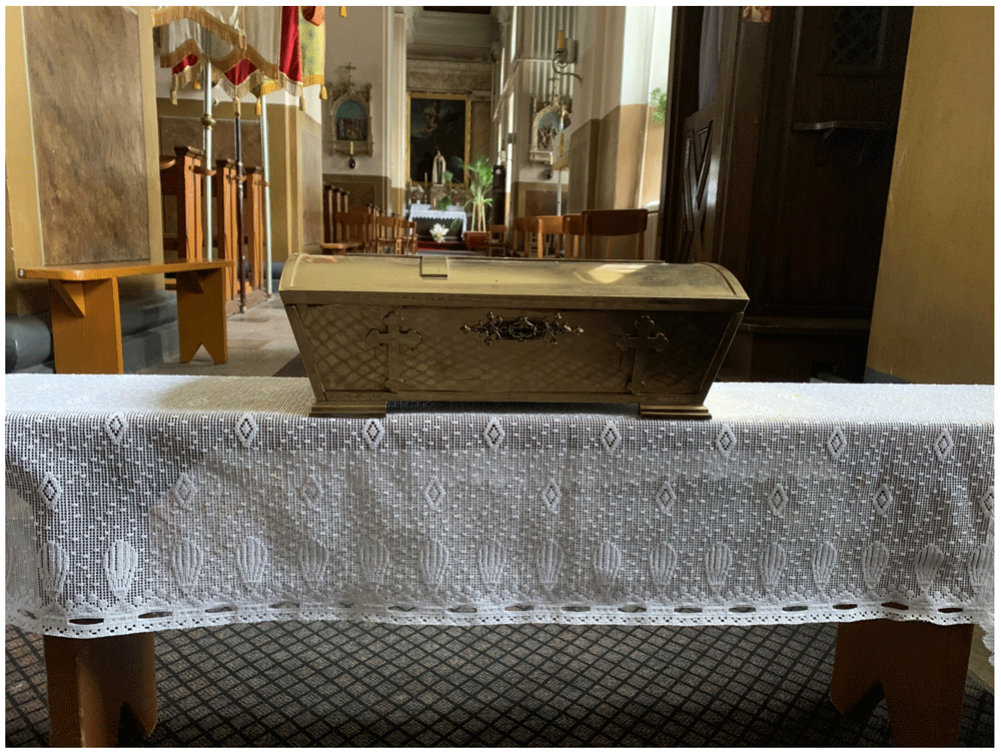
Inner reliquary coffin containing remains of St. Victor relics. Metal (likely brass). Early- to mid-twentieth century. Varakļāni parish church, Latvia. Image: Ruth Sargent Noyes.

St. Victor unfortunately did not survive the destruction wrought upon the northern borderlands of Russia, Estonia, Latvia, Belarus, Poland, and Lithuania, in the course of conflicts during the early twentieth century, a fate shared by many fellow *corpisanti*, whose annihilation metonymized the violence incurred on local human populations of diverse ethnic and religious backgrounds. Whatever remains of Victor is guarded in a small sealed metal casket of modern facture in Varakļāni parish church (Varakļānu Romas katoļu draudze), Latvia ^104^ ( Figure 20– Figure 21).

**Figure 22.**
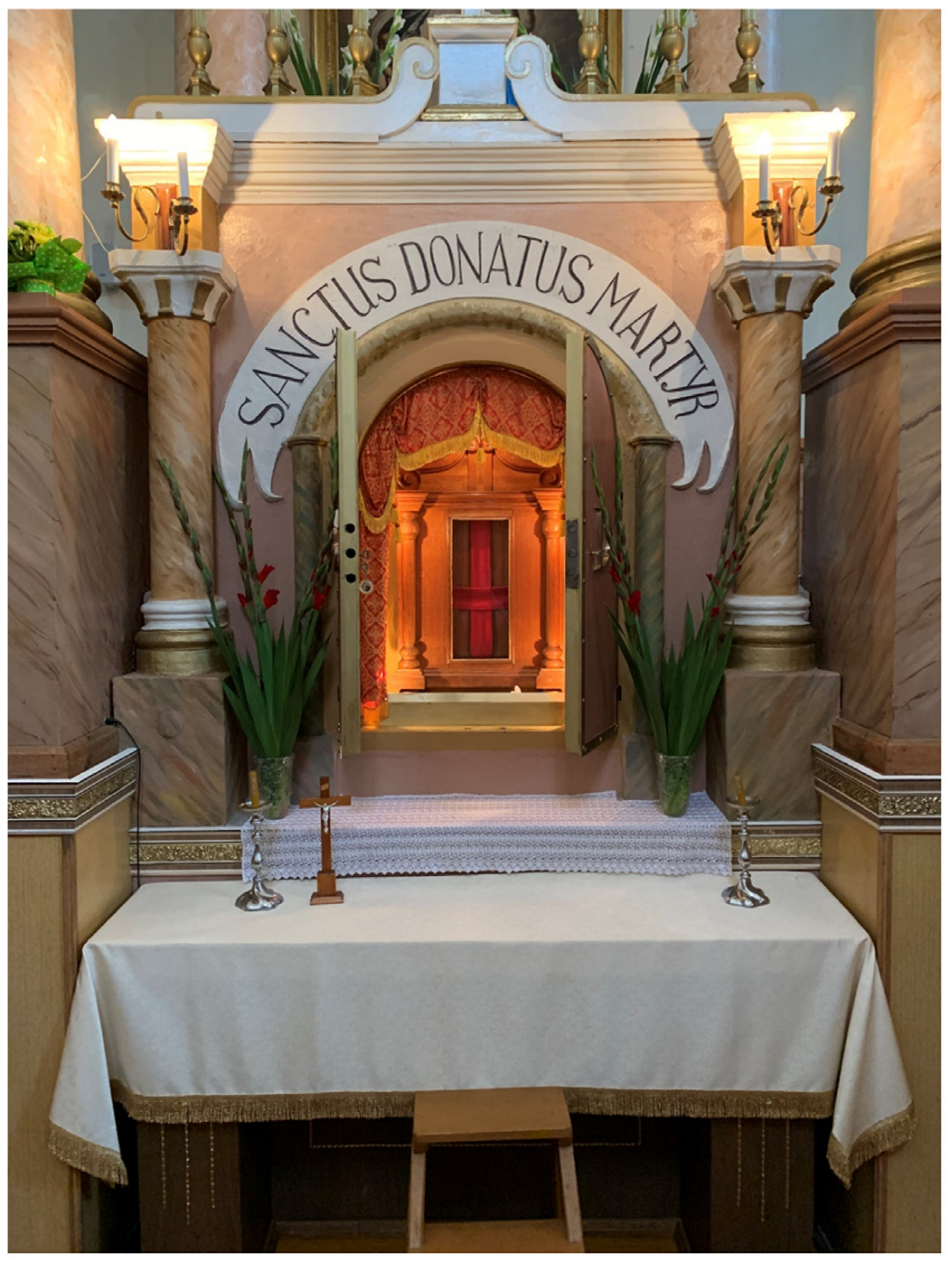
Reliquary tabernacle containing remains of St. Donatus relics. Polychromed wood. Mid-twentieth century. St. Louis Church, Krāslava, Latvia. Image: Ruth Sargent Noyes.

**Figure 23.**
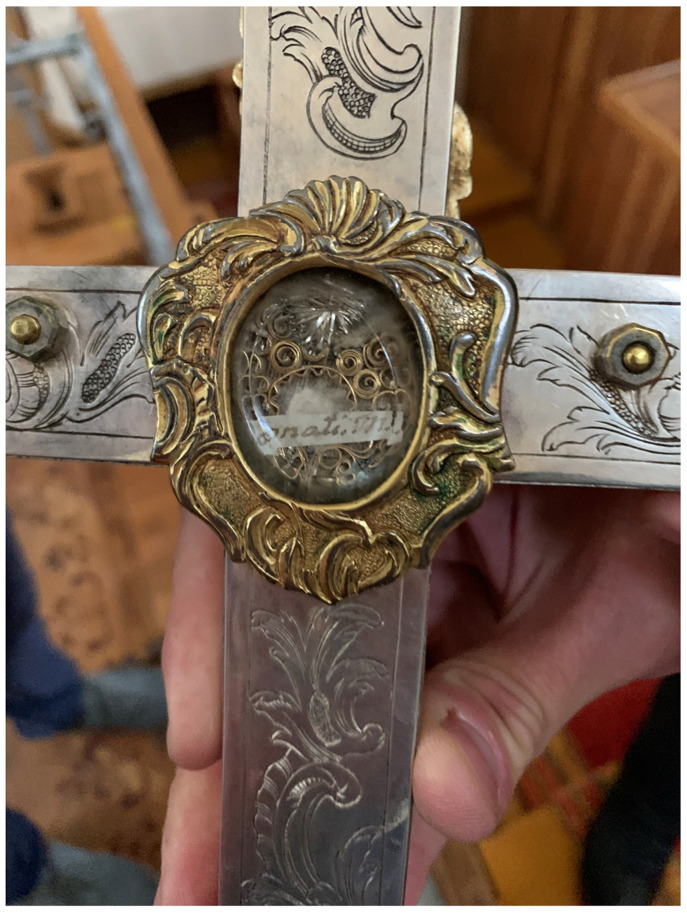
Crucifix with reliquary theca containing St. Donatus relics. Silver, gilding, modern hardware. Late eighteenth century. St. Louis Church, Krāslava, Latvia. Image: Ruth Sargent Noyes.

Likewise devastated was the relic-sculpture of St. Donatus in nearby Krāslava (Pol. Krasław), a town in present-day Latvia that was the center of a latifundium on the Daugava river, and close to that of Varakļāni ^105^. What survived of Donatus in the wake of the Second World War is today kept in a modern tabernacle in St. Louis Church (Krāslavas svētā Ludvika Romas katoļu baznīca) in Krāslava, where the saint is still the focus of a contemporary regional cult, annual festival, and pilgrimage; a tiny relic is also displayed within a glass theca inside a silver crucifix likely dating to the late eighteenth century ^106^ ( Figure 22– Figure 23).

**Figure 24.**
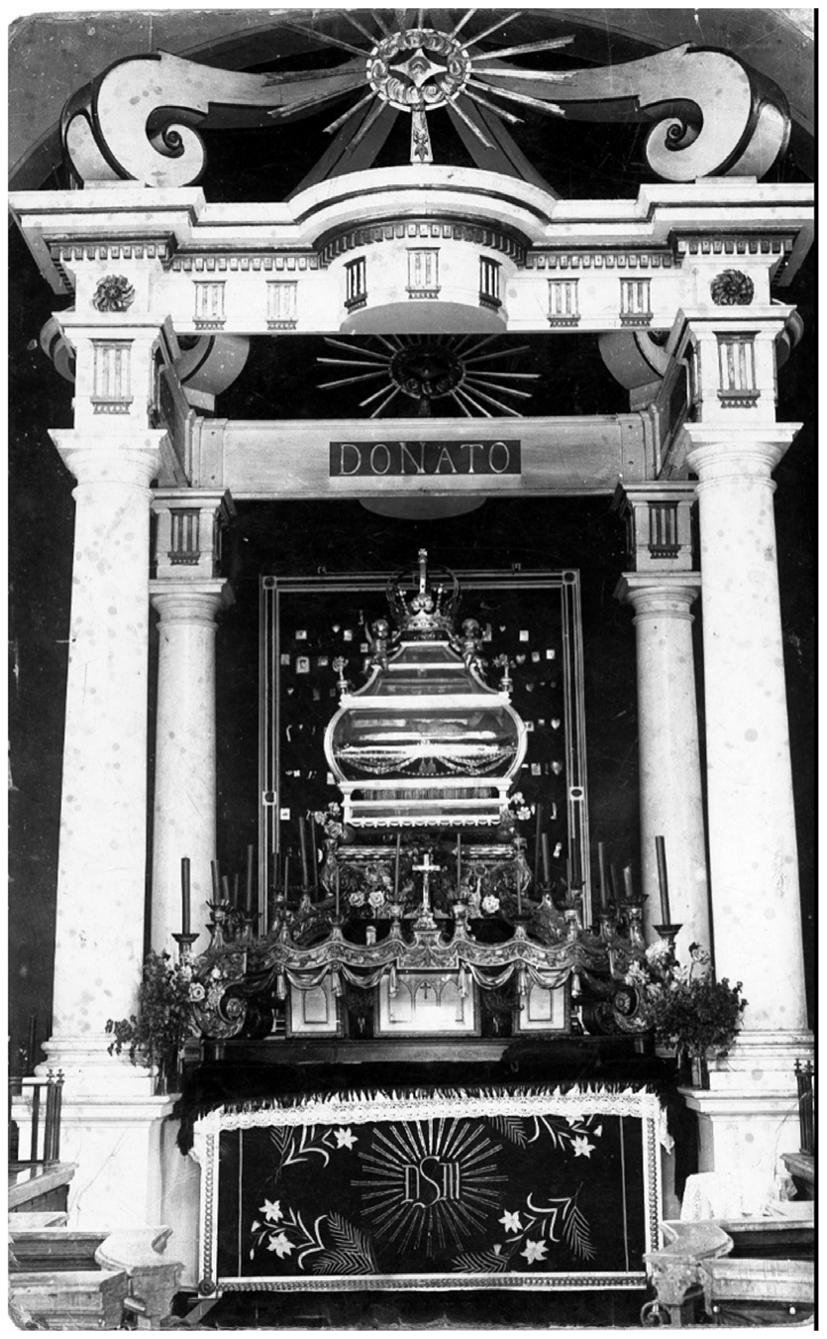
St. Donatus Chapel. Early- to mid-nineteenth century (photo circa 1920s, installation pre-1944). St. Louis Church, Krāslava, Latvia. This figure has been reproduced with permission from: courtesy of Krāslavas vēstures un mākslas muzejs, Krāslavas novada Tūrisma informācijas centrs un Starptautiskais kulinārā mantojuma centrs.

**Figure 25.**
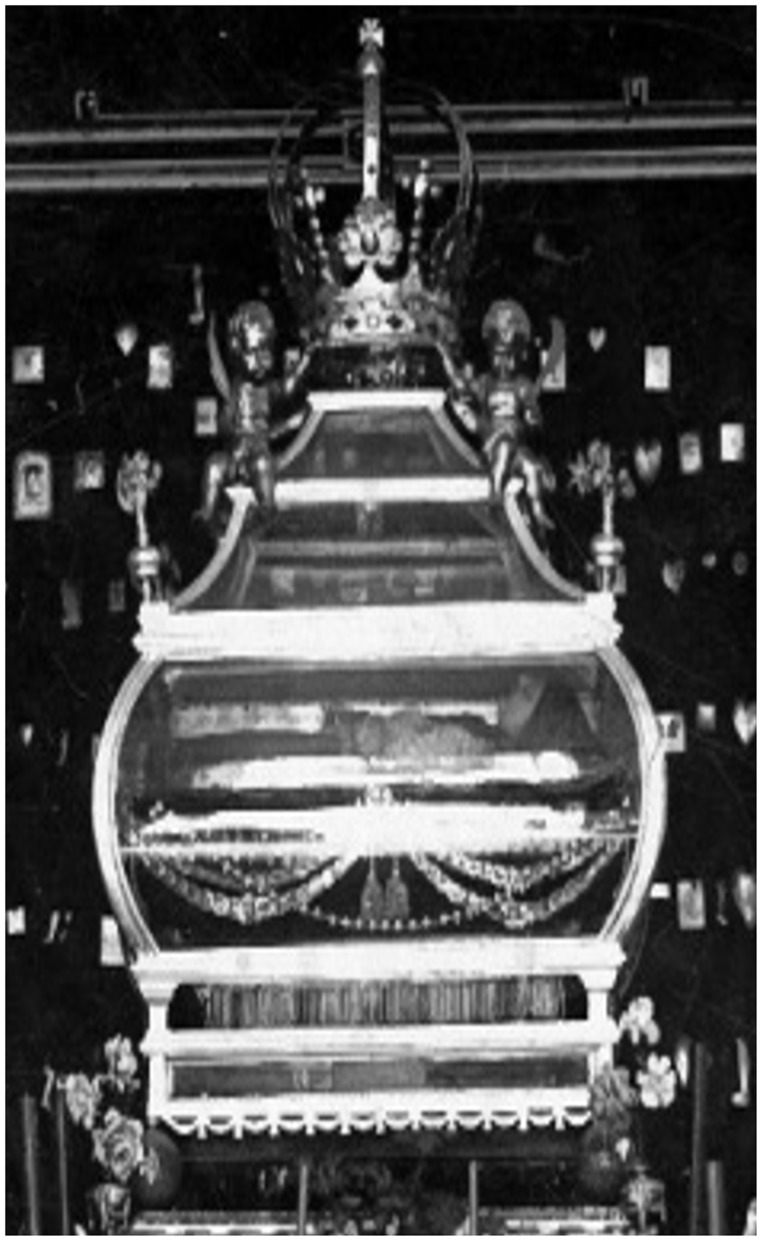
St. Donatus Chapel, detail. Early- to mid-nineteenth century (photo circa 1920s, installation pre-1944). St. Louis Church, Krāslava, Latvia. This figure has been reproduced with permission from: courtesy of Krāslavas vēstures un mākslas muzejs, Krāslavas novada Tūrisma informācijas centrs un Starptautiskais kulinārā mantojuma centrs.

However, Donatus survives in photographs from the early twentieth century, when the figure occupied an exalted position in a glass coffin installed above the altar in an eponymous chapel ( Figure 24– Figure 25). In later the eighteenth century, Krāslava was the private town of the powerful Plater (or Broel-Plater), magnates who like the Borch were of Westphalian origin, presided over vast estates in Inflanty, held top governmental positions in the Grand Duchy, and played key roles in negotiations related to the partitions of Poland-Lithuania ^107^.

**Figure 26.**
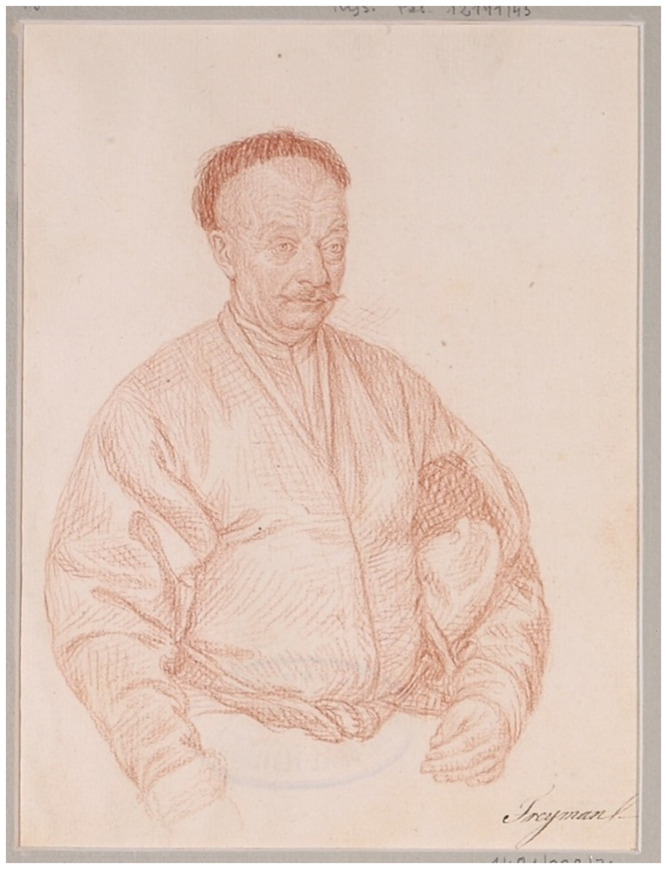
Filippo Castaldi (1734–1814), portrait of Count Konstanty Ludwik Plater. Circa 1778. Sanguine (Red chalk) on paper. *Album Obywateli Inflant*, Rys.Pol.12141, National Museum, Warsaw, Poland. This figure has been reproduced with permission from: courtesy of Department of prints and drawings, National Museum.

**Figure 27.**
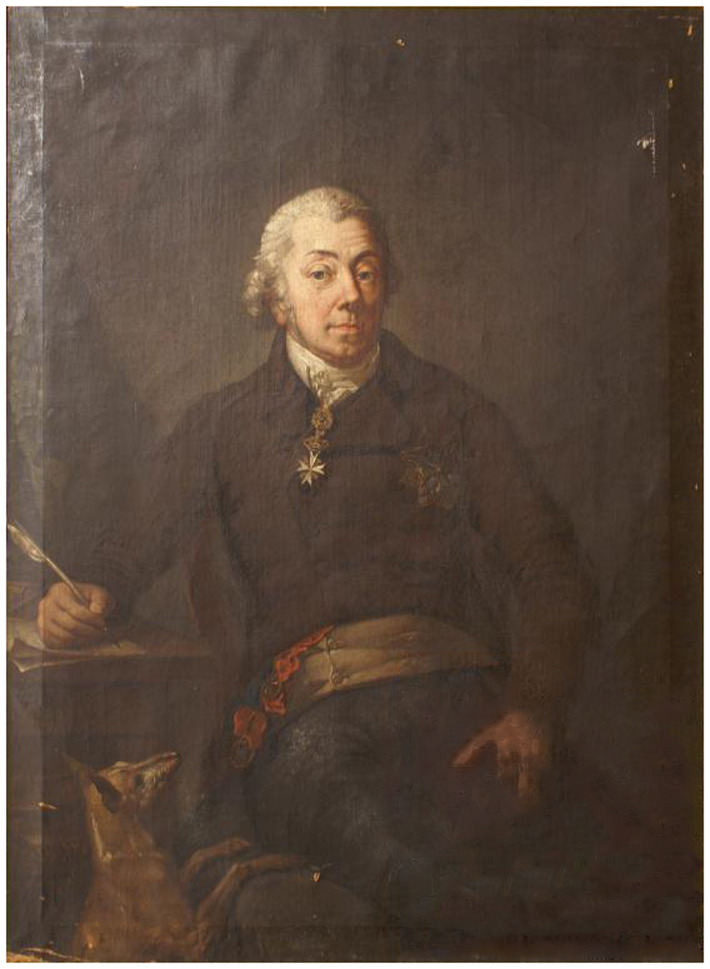
Anton Graff (1736–1813), portrait of Kazimierz Konstanty Plater. Circa 1800. Oil on canvas. The Smolensk State Museum-Preserve, Smolensk, Russia. Image in the public domain: Wikipedia, https://upload.wikimedia.org/wikipedia/commons/7/74/Kazimier_Kanstantyn_Plater._%D0%9A%D0%B0%D0%B7%D1%96%D0%BC%D0%B5%D1%80_%D0%9A%D0%B0%D0%BD%D1%81%D1%82%D0%B0%D0%BD%D1%82%D1%8B%D0%BD_%D0%9F%D0%BB%D1%8F%D1%82%D1%8D%D1%80_%28A._Graff%2C_1800%29.jpg (accessed 15 February 2021).

**Figure 28.**
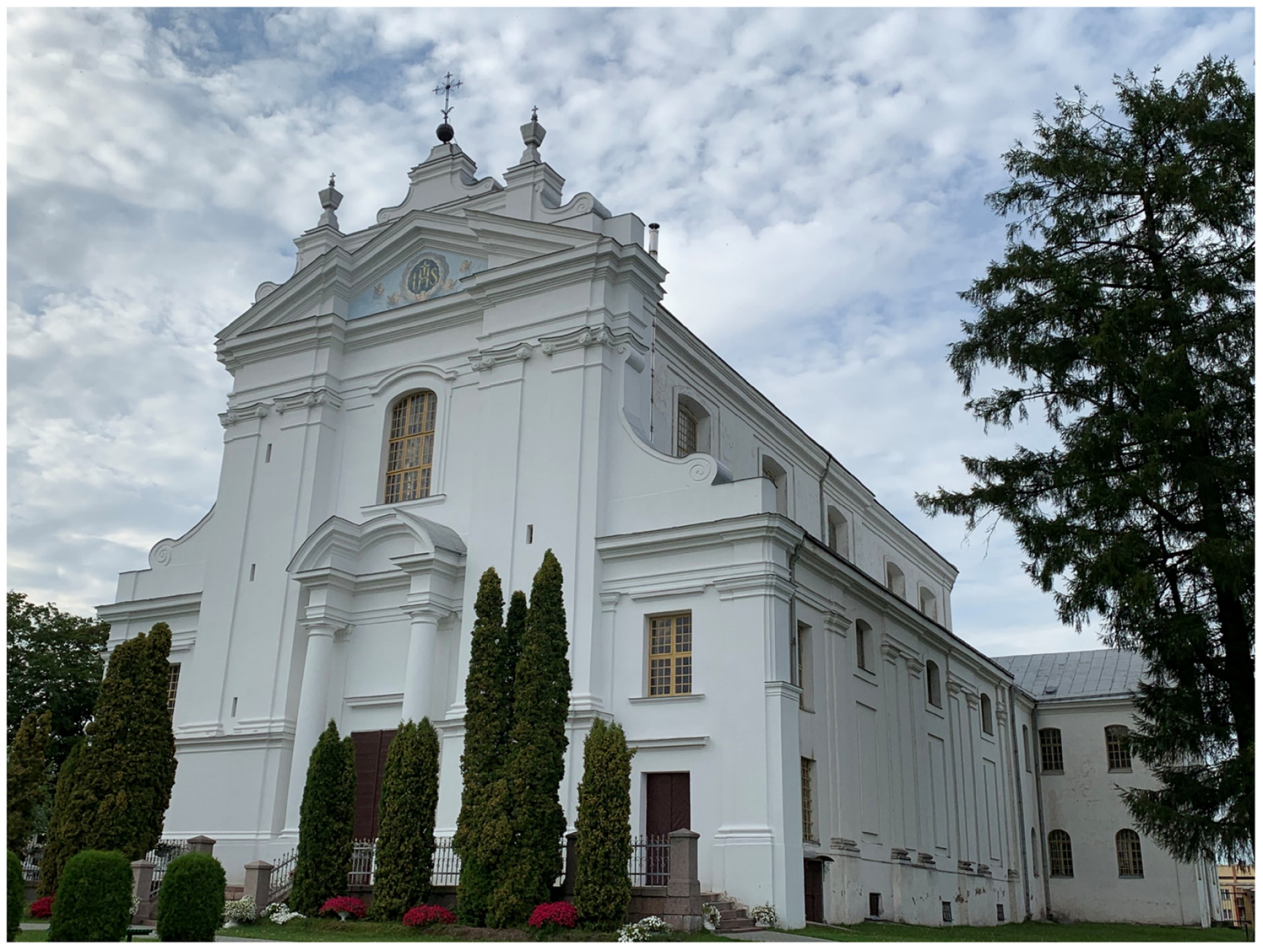
Antonio Ludovico Paracca (1722-c. 1790), Church of St. Louis (attributed). Circa 1755–1767. Krāslava, Latvia. Image: Ruth Sargent Noyes.

Under Count Konstanty Ludwik Plater (1722–78) and his son Kazimierz Konstanty Plater (c. 1749–1807), the Plater staged a renovation of the region’s spiritual and political landscape that proved chameleon-like in its adaptability to changing conditions on the ground and would later serve as a model for regional development emulated by Michał Jan Borch at Varakļāni ^108^ ( Figure 26– Figure 27). Notably, Kazimierz Konstanty Plater married Borch’s sister Izabela Ludwika Borch (1752–1813), closely linking the two families ^109^. Begun as a series of prestigious building projects with the aim of transforming Krāslava’s status to become the new seat of the Bishopric of Livonia, the Plater’s campaign entailed a series of projects in art, architecture and the built environment focused around the town that included a Catholic church dedicated to St. Louis IX (1214–70), pious medieval French monarch, famed Holy Land crusader and collector of relics, and likely designed by the Paracca, a northern Italian family of architects from the Lugano lake region of Lombardy and Plater protégés ^110^ ( Figure 28). The masonry church was sufficiently complete in 1768 to be awarded the status of Cathedral of Livonia by the Polish *sejm* (parliament of nobles) ^111^.

The status of the Plater’s church and latifundia radically changed subsequent to the first partition of 1772, as Krāslava became subordinate to the new Russian diocese of Mohilev initially centered in St. Petersburg. At this moment of uncertainty, the Plater themselves, like Count Michał Jan Borch, traveled to Rome and solicited catacomb relics a few years before Borch arrived at the end of his Grand Tour. However, the Plater undertook their pilgrimage to the *urbe* not in person but by proxy, dispatching another artist-protégé from Inflanty to the peninsula to negotiate on their behalf: their agent was painter Filippo Castaldi (1734–1814), born in Arpino in the province of Frosinone just outside Rome, who had immigrated to the Commonwealth and first appears in the records of Inflanty in 1760, and may have traveled south both for personal reasons and on his patrons’ behalf ^112^. On 22 January 1774 Castaldi wrote from Rome to Kazimierz Konstanty Plater with an account of his hitherto unsuccessful efforts to obtain “a special body of some Saint” ( *un corps particulier de quelque Saint*) through unofficial channels, claiming to have “gone already twice in vain to find [relics] in the catacombs according to established custom and practice” ^113^ (see extended data, Appendix 3). The painter explained the necessity of a papal rescript and a papal audience where he should appear on the Platers’ behalf; he even provided a script—no doubt conveyed to him by a secretary or scribe in the Vatican palace well acquainted with procedure—to be translated into Latin, signed, and sealed by the elder Count Konstanty Ludwik Plater. Lastly, Castaldi enclosed with his letter a gift of two small relics: one of St. Louis King of France and another of the true Cross for Kazimierz Plater’s wife Izabela Ludwika Borch, sister of Count Michał Jan Borch ^114^.

**Figure 29.**
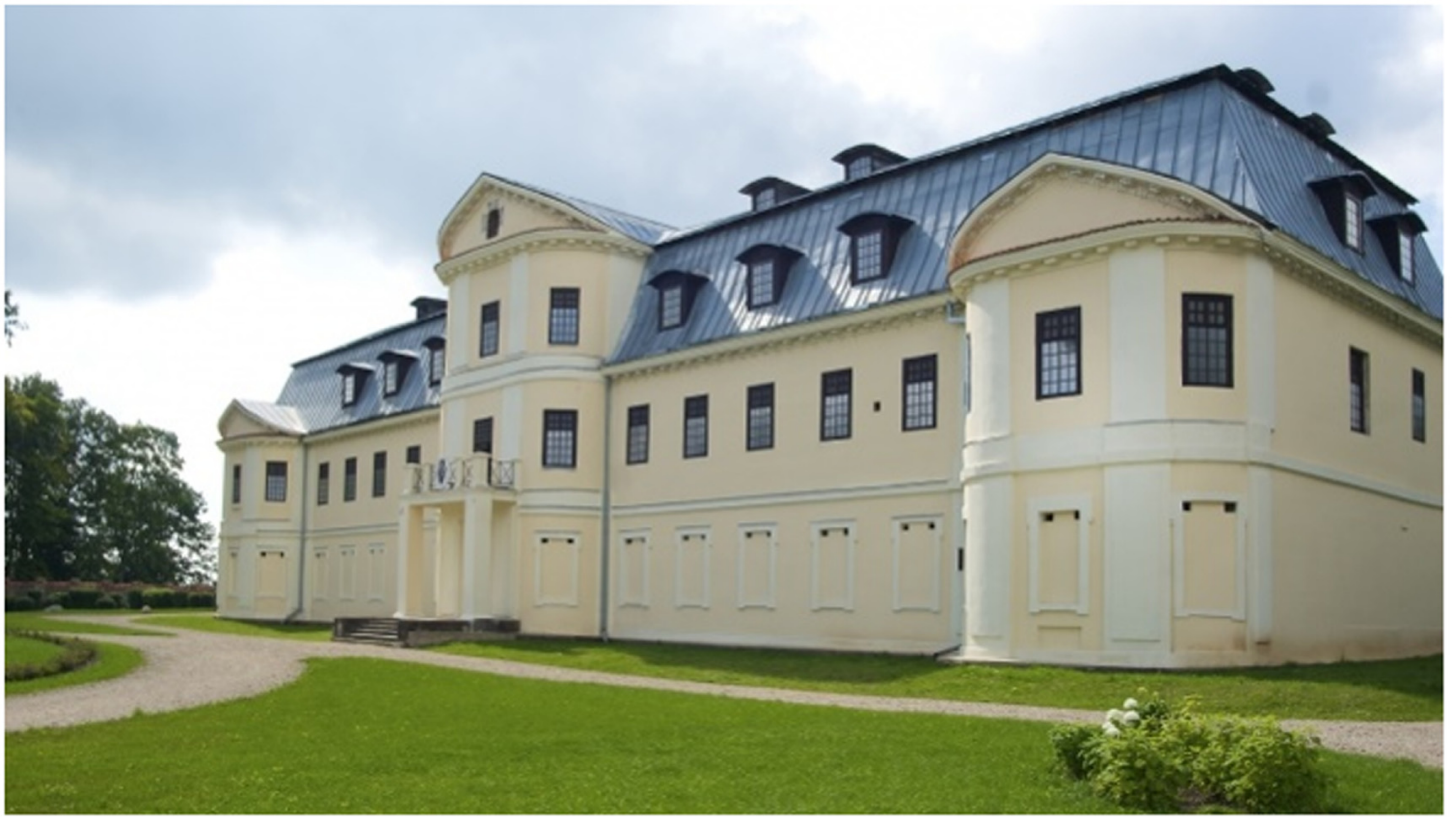
Plater manor palace. Begun c. 1760. Krāslava, Latvia. Image: Ruth Sargent Noyes.

Thus Borch’s own request four years later for the *corposanto* of St. Victor and a true Cross relic was apparently made in direct imitation of what his Plater relations had previously obtained with Castaldi’s help. Indeed, the relic-sculpture of St. Donatus imported to Krāslava likewise represented an ancient Roman soldier martyred for the faith ^115^. Like Borch at Varakļāni, the Plater only oversaw the official *translatio* of St. Donatus to Krāslava’s new church in 1784, the year following Pius’s aforementioned Bull “Onerosa pastoralis.” Before this, they first kept the relic-sculpture in the private chapel inside their palace, surrounded by mural paintings of Roman *vedute* and architectural *quadrature* executed by Castaldi after prints by Gianbattista Piranese, likely souvenirs of his Roman mission for the Plater ^116^ ( Figure 29– Figure 31).

**Figure 30.**
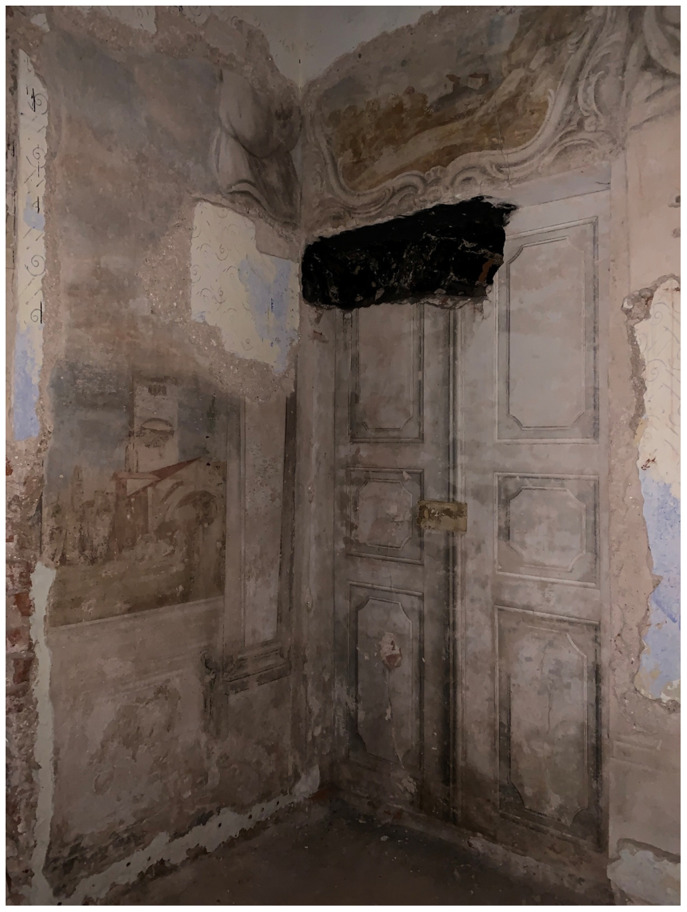
Filippo Castaldi, mural paintings of landscape *vedute* and trompe-l'oeil of classical statues and architectural *quadratura*. *Post* 1760. Plater manor palace, Krāslava, Latvia. Image: Ruth Sargent Noyes.

**Figure 31.**
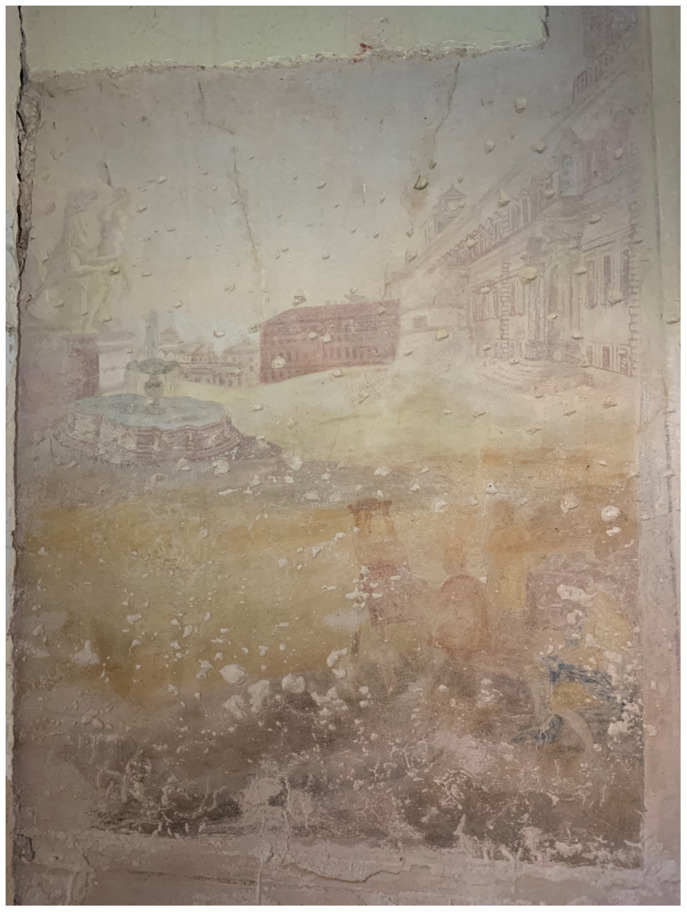
Filippo Castaldi, mural painting *veduta* of the Palazzo Quirinale, Rome. *Post* 1760. Plater manor palace, Krāslava, Latvia. Image: Ruth Sargent Noyes.

In 1790, at the start of Kazimierz Plater’s three-year stint as Castellan of Trakai (Pol. Troki), with a plenary indulgence conceded by Pius VI a new liturgical holiday in the name of St. Donatus was established in the region, prompting construction of an eponymous chapel to safeguard the relic-sculpture and accommodate pilgrims. This project was initiated by Konstanty Plater’s widow Augusta Ogińska (1724–91), whose testament accorded funds for construction of the Donatus chapel and beneath it a subterranean family crypt, a design that gestured to the promise of resurrection through its physical alignment of the decaying corpses of deceased Plater family members with the artificially reintegrated body of their special saint ^117^ (see extended data Appendix 4).

## “some parts of the saintly martyr are visible”

Appearances of *corpisanti* in Baltic territories seem to cluster within certain geographic areas and around noble family relations thrown into relief against the dramatic transformations of Grand Duchy territories immediately prior to and during the Age of Partition. This phenomenon suggests that emulation among local magnates and along axes of intermarriage and religious affiliation may have been a contributing factor in efforts to procure such distinctive relic-sculptures. Konstanty Plater’s wife Augusta Ogińska hailed from the princely family Ogiński, one of the largest and most powerful in the Grand Duchy, who maintained their political stronghold in the Vitebsk (Pol. Witebsk) Voivodeship, an administrative division and local government in the north-eastern Grand Duchy (today divided between Belarus and Russia), not far from Polish Livonia. Two years before the Plater dispatched Castaldi to Rome, in 1772 the *corposanto* of catacomb saint Fortunatus was translated to the Franciscan Church of the Holy Trinity in Syanno (Pol. Sienno) and the pope conceded plenary indulgence for the saint’s feast day on 22 June ^118^. Syanno was a private town of the Ogiński, part of a Vitebsk latifundium annexed by the Russian Empire in the first partition, like those estates in Inflanty ^119^. Fortunatus was procured through the patronage of Castellan of Trakai and Grand Clerk of Lithuania Tadeusz Franciszek Ogiński (1712–1783) and his wives Izabella Radziwiłł (1711–1761) and Jadwiga Załuska (d. 1793) ^120^. That this relic-sculpture and other catacomb relics were significant for the Ogiński is evinced by their mention in personal accounts ^121^.

Similar to the case of the Plater, the importing of St. Fortunatus was apparently executed through a Franciscan middle-man operating on behalf of elite patrons ^122^. The translation of Fortunatus was moreover recorded in two 1772 publications: a devotional booklet and a sermon delivered on the occasion ^123^ (see extended data, Appendix 5). Both printed in Vilnius on the Franciscan presses, these hagiographies signal efforts invested in promoting the broader importance of the catacomb saint’s nascent cult on both a regional and national level in Lithuania. Ogiński was furthermore responsible for the catacomb relics of St. Teofilus (or Theophilus), translated in 1680 in a silver coffin to the Church of St. Johns in Vilnius; and others of unnamed saints in 1768 to the Trinitarian monastery in Maladzyechna (Pol. Mołodeczno), a town in the Minsk Vovoideship (today in Belarus) purchased by the family early in the century ^124^. These three strategically located locations manifested the magnates’ power and presence throughout the Grand Duchy, from its capital to its provinces, and cast a numinous net across their far-flung territories that reified a transhistorical notion of *translatio imperii*, whereby the glory and sacral power of Rome might be transferred to the far north, a concept nurtured in historiographical discourse of this period ^125^.

The Franciscans, in turn, represent a direct connection to a still earlier case of catacomb relic-sculptures that may have inspired the emulation of the Ogiński: the aforementioned Franciscan monastery in Valkininkai that received an array of catacomb relics (detailed above) in the form of small fragments and *pasta di reliquie*, was also endowed with the *corposanto* figure of martyr-soldier St. Bonifacus, excavated from the catacombs of St. Agnese and imported by Provincial Superior Bonawentura Bunielski in 1765 ^126^. The Valkininkai Franciscans were under the protection of Great Hetman of the Crown Józef Potocki (1673–1751), among the wealthiest magnates in the Commonwealth of the period, and his wives Wiktoria Leszczyńska (d. 1732) and Ludwika Mniszech (1712–1785), thus offering a striking precedent for emulation by the Ogiński ^127^. Funds provided through the testament of Wiktoria furnished an oak and glass coffin lavished with rococo ornaments to contain Boniface, while Ludwika was involved in the solemn *translatio* to the Franciscan church on 28 June 1766, after inspection and authentication of the relics by Vilnius Bishop Ignacy Jakub Massalski (1727–1794) on 29 November 1765. Evidence of these events and the Franciscans’ promotion of Bonifacus’s cult can be found in a devotional booklet they published in Vilnius in 1770, *O Sprowadzeniu I złożeniu ciała Świętego Bonifacego Męczennika w Kościele Olkinickim Franciszkańskim krótka Wiadomość* ( *On the Bringing and Deposition of the Body of Saint Bonifacus Martyr to Olkiniki [Valkininkai] Franciscan Church*), which includes a relation of the 1766 translation ^128^ (see extended data, Appendix 7).

**Figure 32.**
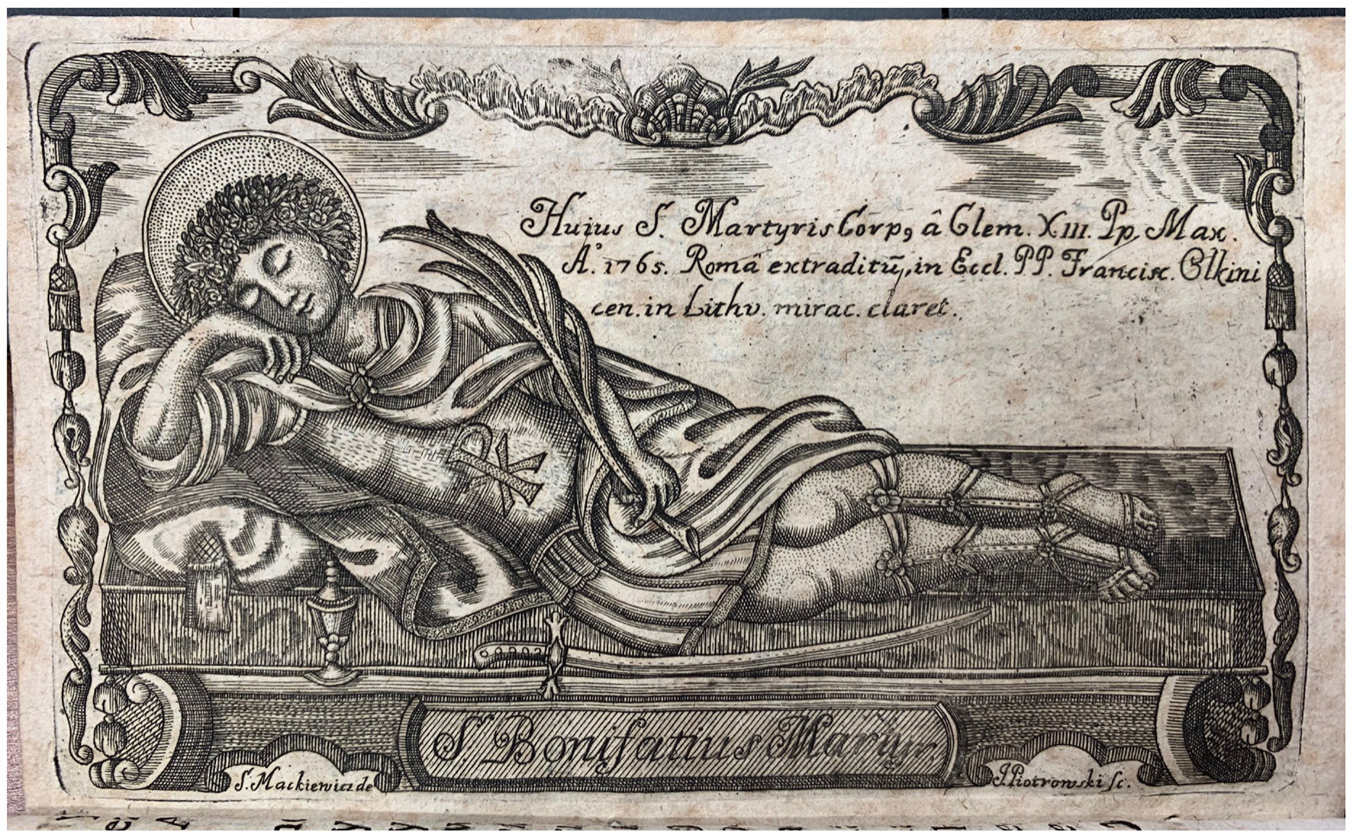
J. Piotrowski after a design by S. Mackiewicz, “the body of the holy martyr St. Bonifacus exported from Rome by Pope Clement XIII [Carlo della Torre di Rezzonico, 1693–1769; r. 1758–1769] in 1765.” Circa 1770. Etching and engraving on paper. In *O Sprowadzeniu I złożeniu ciała Świętego Bonifacego Męczennika w Kościele Olkinickim Franciszkańskim krótka Wiadomość.* Vilnius: W Drukarni J K M. XX. Franćiszkanow, 1770. Vilnius University Library, Rare books collection, IV-22490. Image: Ruth Sargent Noyes.

This publication is important not only for its detailed textual account, but also for its visual representation of the relic-sculpture in its original state as it arrived from the papal city: a print etched and engraved by J. Piotrowski after a design by S. Mackiewicz identifying “the body of the holy martyr St. Bonifacus exported from Rome by Pope Clement XIII [Carlo della Torre di Rezzonico, 1693–1769; r. 1758–1769] in 1765” was bound in the slim volume ( Figure 32). It shows the relic-sculpture of a male figure dressed in the guise of an ancient soldier and lounging on its side with elbow bent upon a silken mattress, its head reclining on fringed cushions and right hand, while its left hand grips the palm of martyrdom; arranged on the mattress are a sword and ampoule of blood. This design already in 1765 clearly reflected the common morphology shared by the serially manufactured *corpisanti* in the final decades of the century, suggesting that the chronology dating these relic-sculptures to a date after 1772 should be reconsidered.

A nineteenth-century church inventory recorded not only the manner of the installation of the glass coffin on the altar *mensa*, but also the ornamentation and appearance of the relic-sculpture itself:

“…some parts of the saintly martyr are visible: the head, both shins, and two arm bones, all dressed in knight's armor. The cape is made out of red velvet, lined with white brocade, embellished with wide gold galloon, with sleeves made out of silver net and shoes and socks made from the same material, and next to each of the shoes and socks there are four Czech green stones, whereas on the chest – a star of the white eagle, embroidered in gold by Mychal Granovsky, the sword is wooden, plated with silver. Hands are made from Czech glass, ampoule with blood of this Saint....” (see extended data, Appendix 6)

**Figure 33.**
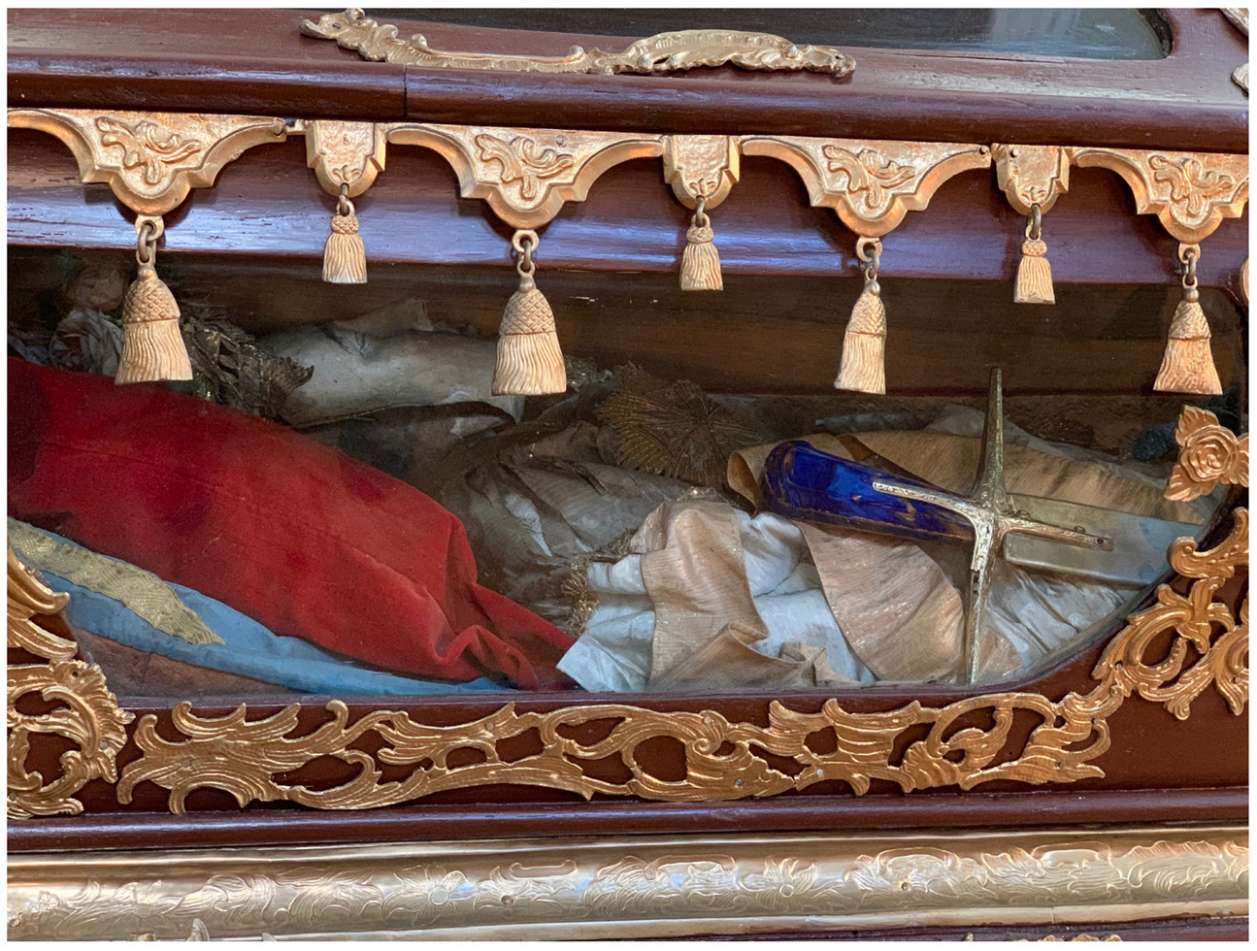
*Corposanto* relic-sculpture of St. Bonifacus. Human remains (skeletal fragments), metal wire, wood, wax, textile, polychromy, laid paper, and mixed materials. Second half of the eighteenth century ( *post* 1765), altered late nineteenth century. Formerly Franciscan convent in Valkininkai. Valkininkai Parish Church, Lithuania. Image: Ruth Sargent Noyes.

Crucially, this description underscores what would become by the end of the century the typical mode of fabrication of *corpisanti*, whereby portions of the skull and bones of the lower shins or wrists were attached to the wire or wooden armature constituting the underpinning ”skeletal” structure and left visibly exposed for maximum effect. Valkinainkai further constitutes an important case because unlike many other examples of *corpisanti* from the period, this relic-sculpture has survived largely intact until today, having been moved from the monastery (since destroyed) to the local parish church in the late nineteenth century; today this unique sacral artwork has been transferred to the Church Heritage Museum in Vilnius, where at the time of this study it is presently the object of analysis and conservation ^129^ ( Figure 14, Figure 33– Figure 40).

**Figure 34.**
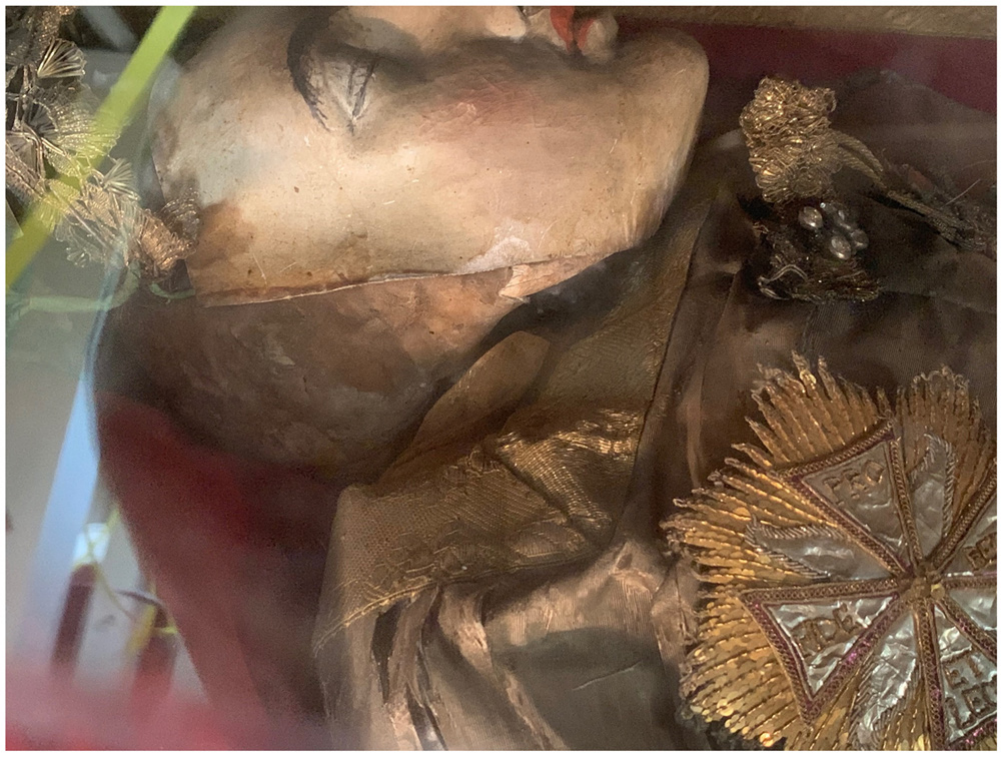
*Corposanto* relic-sculpture of St. Bonifacus. Human remains (skeletal fragments), metal wire, wood, wax, textile, polychromy, laid paper, and mixed materials. Second half of the eighteenth century ( *post* 1765), altered late nineteenth century. Formerly Franciscan convent in Valkininkai. Valkininkai Parish Church, Lithuania. Image: Ruth Sargent Noyes.

**Figure 35.**
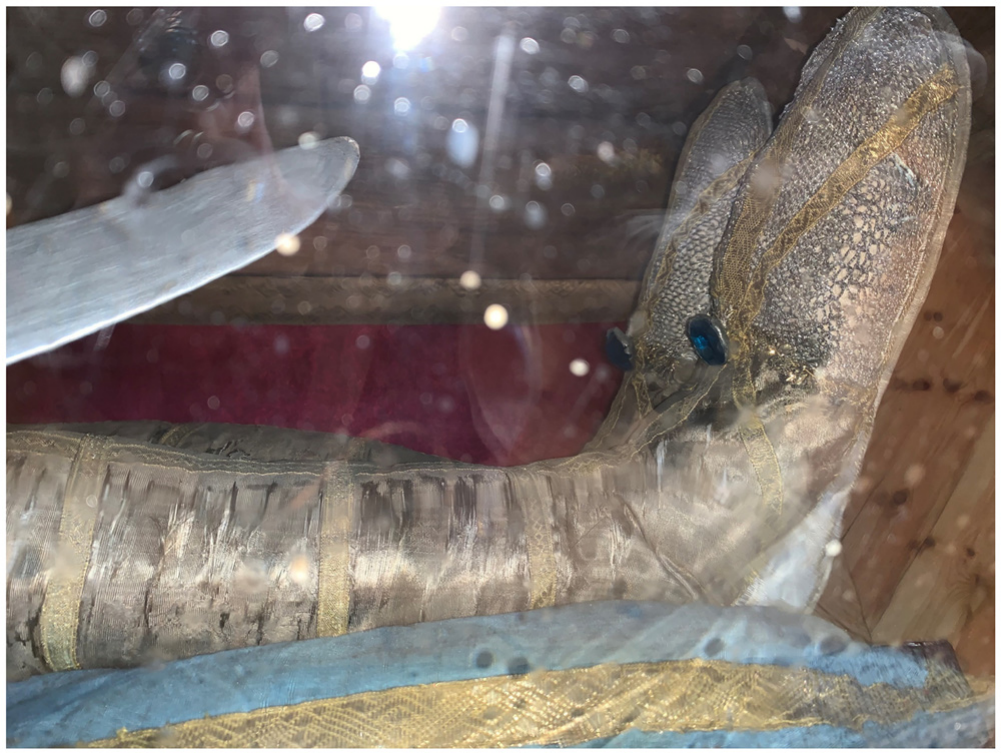
*Corposanto* relic-sculpture of St. Bonifacus. Human remains (skeletal fragments), metal wire, wood, wax, textile, polychromy, laid paper, and mixed materials. Second half of the eighteenth century ( *post* 1765), altered late nineteenth century. Formerly Franciscan convent in Valkininkai. Valkininkai Parish Church, Lithuania. Image: Ruth Sargent Noyes.

**Figure 36.**
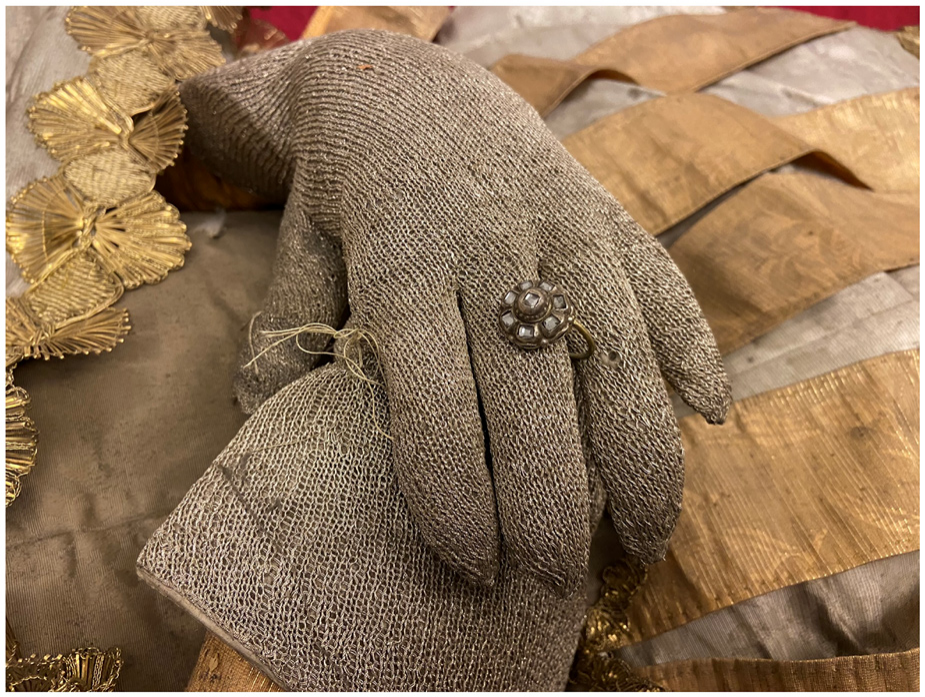
*Corposanto* relic-sculpture of St. Bonifacus. Human remains (skeletal fragments), metal wire, wood, wax, textile, polychromy, laid paper, and mixed materials. Second half of the eighteenth century ( *post* 1765), altered late nineteenth century. Formerly Franciscan convent in Valkininkai. Valkininkai Parish Church, Lithuania. Image: courtesy of Sigita Maslauskaitė-Mažylienė.

**Figure 37.**
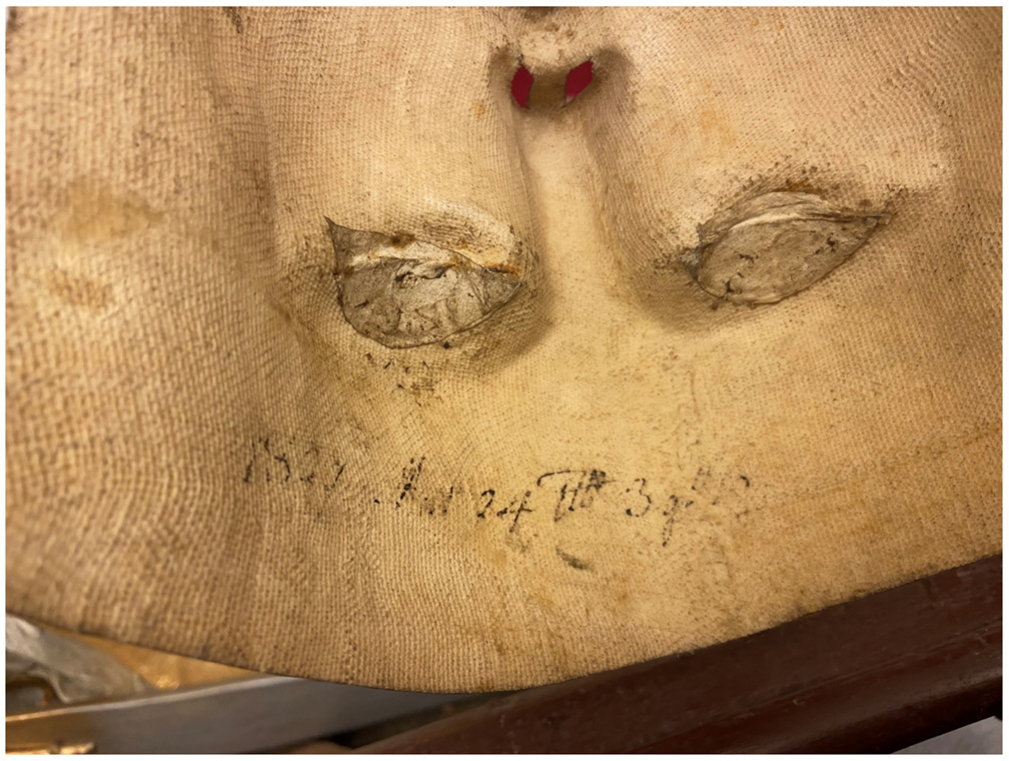
*Corposanto* relic-sculpture of St. Bonifacus. Human remains (skeletal fragments), metal wire, wood, wax, textile, polychromy, laid paper, and mixed materials. Second half of the eighteenth century ( *post* 1765), altered late nineteenth century. Formerly Franciscan convent in Valkininkai. Valkininkai Parish Church, Lithuania. Image: Ruth Sargent Noyes.

**Figure 38.**
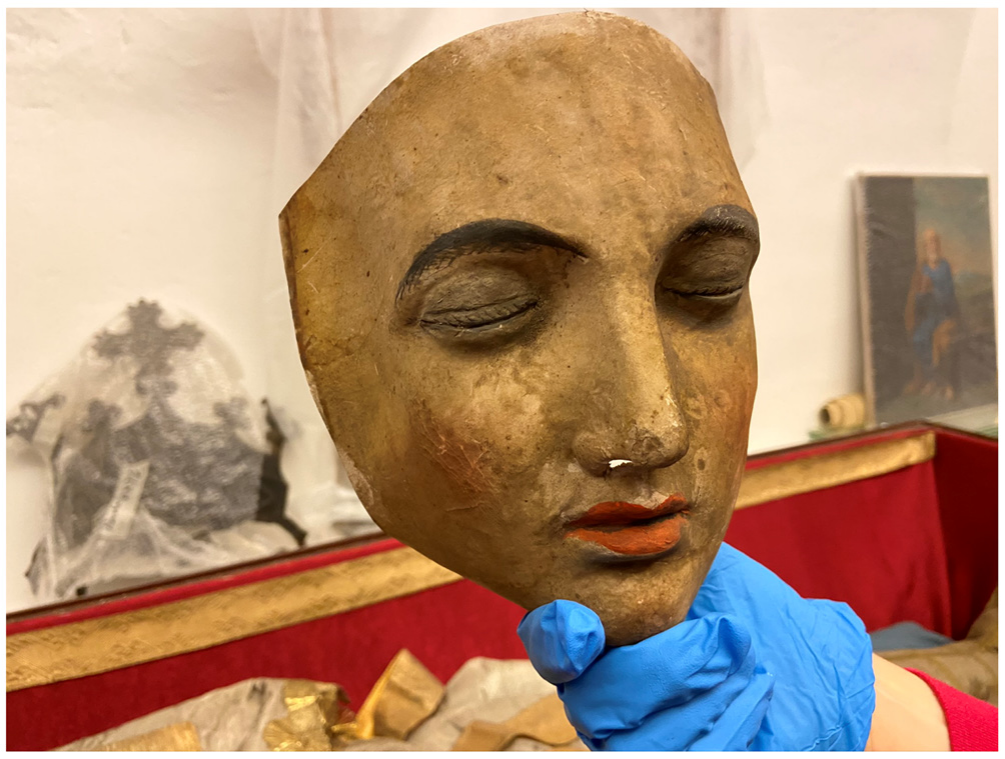
*Corposanto* relic-sculpture of St. Bonifacus. Human remains (skeletal fragments), metal wire, wood, wax, textile, polychromy, laid paper, and mixed materials. Second half of the eighteenth century ( *post* 1765), altered late nineteenth century. Formerly Franciscan convent in Valkininkai. Valkininkai Parish Church, Lithuania. Image: courtesy of Sigita Maslauskaitė-Mažylienė.

**Figure 39.**
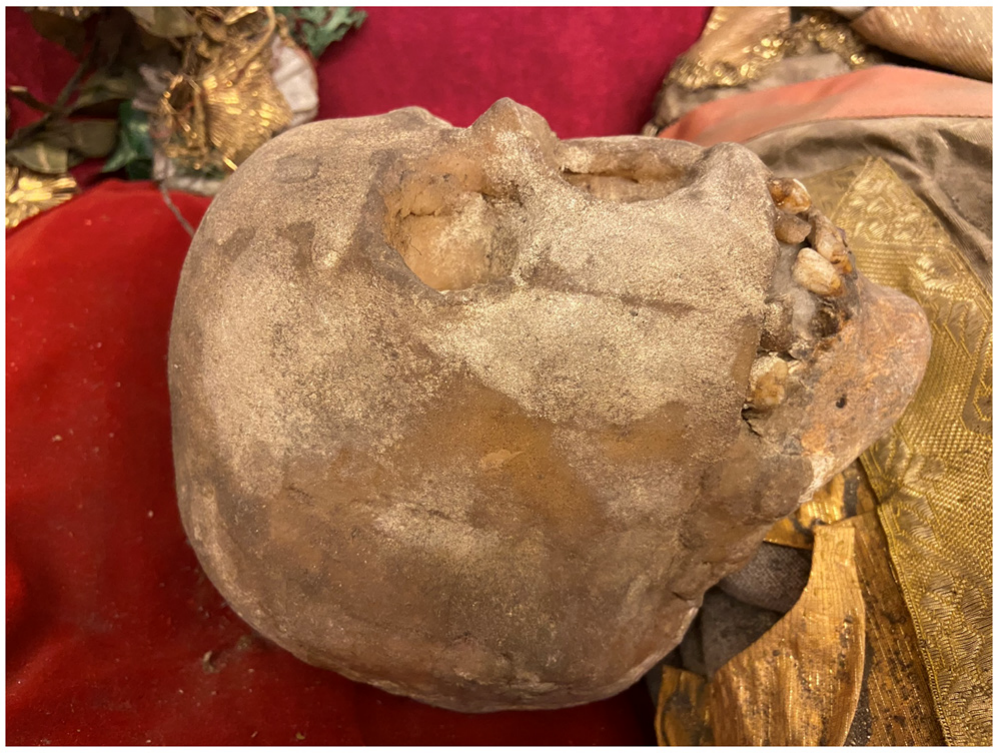
*Corposanto* relic-sculpture of St. Bonifacus. Human remains (skeletal fragments), metal wire, wood, wax, textile, polychromy, laid paper, and mixed materials. Second half of the eighteenth century ( *post* 1765), altered late nineteenth century. Formerly Franciscan convent in Valkininkai. Valkininkai Parish Church, Lithuania. Image: courtesy of Sigita Maslauskaitė-Mažylienė.

**Figure 40.**
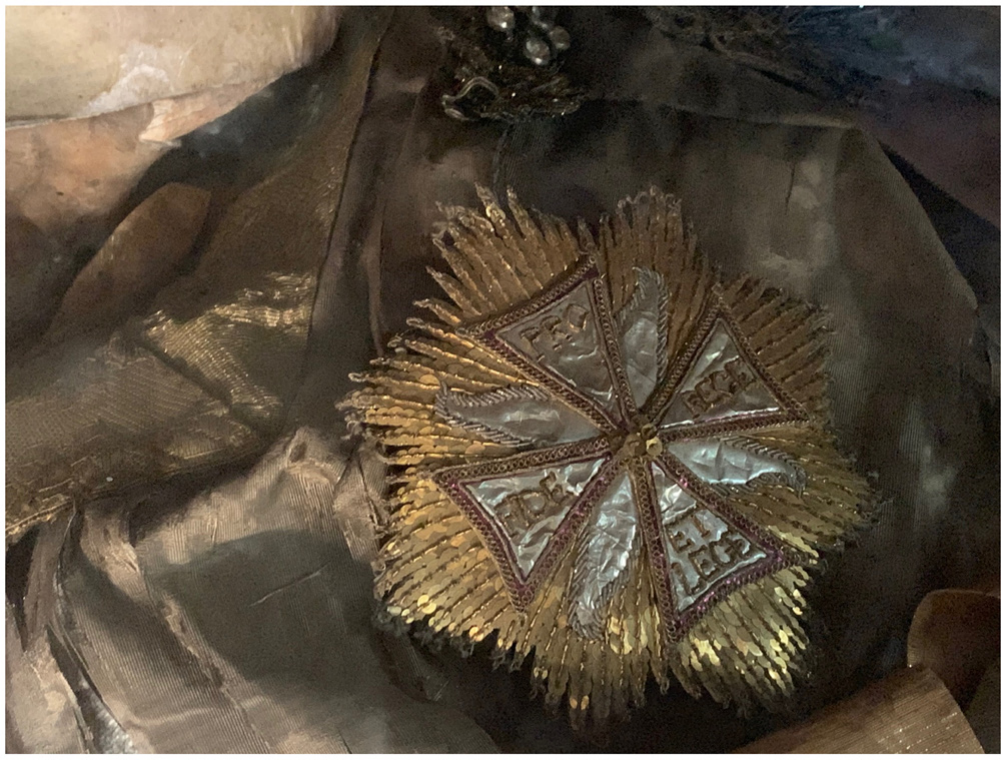
*Corposanto* relic-sculpture of St. Bonifacus. Human remains (skeletal fragments), metal wire, wood, wax, textile, polychromy, laid paper, and mixed materials. Second half of the eighteenth century ( *post* 1765), altered late nineteenth century. Formerly Franciscan convent in Valkininkai. Valkininkai Parish Church, Lithuania. Image: courtesy of Sigita Maslauskaitė-Mažylienė.

It is clear from photos that Bonifacus in its current state has undergone significant changes since its early depiction in the 1770 print: the general posture has been completely alterred from the initial position as it would have left the Roman workshops. This was likely done at an early moment in its history, in conjunction with the initial installation to fit in the specially donated coffin. The accompanying ampoule, martyrial palm frond, and red velvet cape have been lost and the entire figure redressed, most likely in the course of later (probably nineteenth-century) restoration efforts by the local community.

Still preserved are the ornamental sword—probably of Roman manufacture—and the embroidered star of Poland's highest order, the Order of the White Eagle (Pol. Order Orła Białego), most certainly added on the occasion of the 1765 *translatio* to represent Potocki’s membership ^130^ ( Figure 37).

The same restoration that redressed Bonifacus also furnished the figure with a fresh face, in the form of a molded, polychrome canvas mask inscribed with the year 1899 on its reverse ( Figure 38– Figure 39).

Such a provision was no doubt made because the original head of the sculpture had deteriorated to the point of violating Church decorum; in fact, the unmasked cranium as it is today presents a strong impression of eighteenth-century pastiche, with only a partial mandible bone and a few teeth visible ( Figure 40). It was common manufacturing process to reconstitute and reshape often extremely fragmentary cranial bones quarried from the catacombs into the semblance of a complete skull using an amalgum of plaster, glue, dessicated milk powder, and other malleable materials, or even papier maché ^131^.

## “with unquestionable signs of martyrdom”

**Figure 41.**
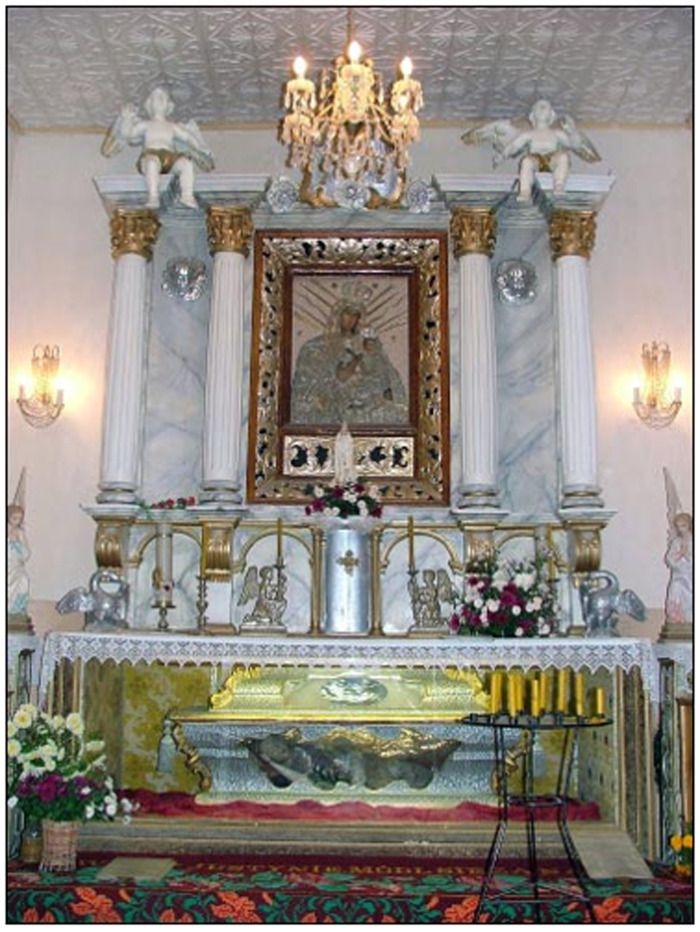
*Corposanto* relic-sculpture of St. Justinian. Human remains (skeletal fragments), metal wire, wood, wax, textile, polychromy, laid paper, and mixed materials. Second half of the eighteenth century ( *post* 1754). Formerly Church of the Discalced Carmelites in Old Myadzyel. Mosar Parish Church, Belarus. Image in the public domain: http://www.radzima.org/eng/object-photo/4815.html (accessed 15 February 2021).

A final case of *corpisanti* in the Baltics significant both for reasons of dating and survival is that of St. Justinian (or Justin) in Mosar (Pol. Mosarz), a village in present-day western Belarus, where the relic-sculpture was transferred in the nineteenth century from its original position in the church of the Discalced Carmelites in Old Myadzyel (Pol. Stary Miadzioł, hereafter Myadzyel) ^132^. Today Justinian in Mosar, like Donatus in Krāslava, remains a center of pilgrimage activity in the region ^133^ ( Figure 41). Within the Grand Duchy of Lithuania, Myadzyel was part of the Vilnius Voivodeship and was spared from the first partition of 1772. In the late seventeenth century the town and surrounding properties were acquired by a rising clan from Vitebsk in the north-east, the Koszczyc, who obtained privileges from Polish King King Augustus III in 1736 for the town. In 1754 Starost of Zarzecze ^134^, Antoni Tadeusz Koszczyc (b. 1720), founded a community of Discalced Carmelites in Myadzyel. For this, he had erected a masonry monastery and church of Our Lady of the Scapular (consecrated on 15 August), and also journeyed to Rome for the Holy See’s sanction by Pope Benedict XIV (Prospero Lorenzo Lambertini, 1675–1758; r. 1740–1758). From Benedict XIV, Koszczyc likewise obtained the catacomb relics of St. Justinian, one of the oldest *corpisanti* still preserved today in more or less original form, thanks in part to a local cult in Mosar in the early twentieth century ^135^.

Despite its early date relative to the established corpus of this typically late eighteenth-century art form, the relic-sculpture shares morphological similarities with later examples, demonstrating that *corpisanti* were already well in production and becoming established as a distinct genre by mid-century. Although Justinian’s advent in the Grand Duchy predated the partitions of the Commonwealth by some decades, evidence suggests there was renewed bidirectional activities between Myadzyel and Rome involving the saint’s cult soon after the first partition in 1772: in April 1773 Pope Clement XIV issued a papal brief establishing a brotherhood of St. Justinian ^136^. In 1780, perhaps following the example of hagiographies printed for Bonifacus and Fortunatus, the devotional booklet *Nabozenstwo Do S. Justyna Męczennika* ( *Devotion to St. Justin Martyr*) was published in Vilnius ^137^ (see extended data, Appendix 8). The text sets a brief *vita* of the relatively obscure martyr, an adolescent killed under Emperor Diocletian, against an account of the heroic and saintly pilgrimage of Antoni Koszczyc to procure the relics:

“…Such saintly efforts, supported by a fortunate desire, received a result: the Holy Father Benedict XIV was graciously persuaded, and he ordered to look for bodies of the Holy Martyrs in the aforementioned cemetery [of St. Sebastian]. Soon those bodies, buried for one thousand four hundred and fifty years, have been found. The whole body of one willingly fallen in his young years for Christ's faith, saint Justinian's miraculous bones, with unqestionable signs of martyrdom: for which the Most High Shepherd of the Church gave thanks to God [and] gave this holy gift to the greater honor and glory of God to … Lord Antoni Koszczyc the Starost of Zarzecze.”

The mention of “unqestionable signs of martyrdom” indicates the probable forensic examination by Vatican experts of the skeletal remains incorporated into this *corposanto*, a practice mentioned above that emerged as part of the catacomb relic regulatory system in Rome. Likewise pointing to Roman origins are the circumstances of Justinian’s installation today in Mosar beneath the altar *mensa*. This reflects a widespread practice throughout the Commonwealth for displaying whole-body relics within a so-called *confessio* altar design, with the relics behind or beneath the altar table ^138^. This reliquary altar configuration resonated the biblical account in the Book of Revelation 6:9 (“I saw there, beneath the altar, the souls of all who had been slain for love of God’s word and of the truth they held”) and had its origins in the papal city ^139^. Catholic Counter-Reformation historians maintained this was an ancient Christian liturgical architectural arrangement first initiated in the *urbe* for the conservation and ritual display of martyrs’ relics, to mark the site where the first saints “confessed” and died for their faith ^140^.

## Conclusion: “relic states”

This article has undertaken to shed new light on an overlooked but significant chapter in the intersecting histories of the Catholic cult of relics and cross-cultural relations between the Italian peninsula and the Baltic littoral, by mapping the invention, production, and distribution of *corpisanti* catacomb relic-sculptures from papal Rome against elite religio-political networks in Inflanty and the Lithuanian Grand Duchy, during a watershed period when constituencies involved faced new horizons of territorial and cultural degeneration. This initial study has also aimed to provide an overview of the research landscape on this interdisciplinary and intercultural topic, including offering an array of primary source texts in translation as appendices, which constitute a methodological essay on the issue of what kinds of multifaceted investigative strategies might be most effective for analyzing this multidimensional subject. Hardly exhaustive, this study might furnish a point of entry into what is a broad phenomenon and indicate fruitful ground for future research. There are numerous cases of Baltic *corpisanti* that remain to be contextualized and indeed identified in the north: in the territory of present-day Belarus, for example, it is known from nineteenth-century church inventories that the *corposanto* of St. Clemens (or Clement) was once in the parish church in Kyemyelishki (Pol. Kiemieliszki), not far from Vilnius (now the Hrodna region in Belarus), and the *corposanto* of another St. Clemens still survives in Hrodna (Pol. Grodno), the latter brought by Karol Litawor Chreptowicz (d. 1801/1802), starost of Hrodna, Lithuanian field guard, and ensign at the Lithuanian court ^141^ (see extended data, Appendix 9). Chreptowicz obtained the relics during a pilgrimage to Rome in 1767, when in a papal audience with Clement XIII the starost protested destruction wrought in his lands by Russian forces and solicited catacomb relics of a martyred soldier; the Authentic was issued by the Cardinal Vicar on 16 January, 1768 for remains from the catacomb of St. Priscilla ^142^. Chreptowicz only returned to Hrodna with Clemens in 1780, which occasioned another authentic manuscript prepared under Bishop of Vilnius Ignacy Jakub Massalski, who then oversaw the translation to the Brigittine monastery church during a three-day indulgenced festival on 3–5 June 1781 ^143^. The festivities and *translatio* were recorded in the hagiographical booklet, *Solenna introdukcya ciała S. Klemensa Męczennika* ( *Solemn introduction of the body of S. Clement Martyr*) ^144^ (see extended data, Appendix 10). In another case requiring further research, it is clear from the Roman archives of the Custodian of the Cardinal Vicar that in July 1778, Count Manuzschi (or Manuzzi), starost of Opsa (then in the diocese of Vilnius, now in Belarus, Vitebsk region), was granted the *corposanto* of St. Coronatus after a papal audience ^145^ (see extended data, Appendix 11). While the details of the fate of Coronatus after being quarried from the Catacombs of San Ciriaco in Via Ostiense remain to be determined, it should be noted that the supplicant in this instance was husband to a possible mistress of the last King of the Polish-Lithuanian Commonwealth, Stanisław August Poniatowski ^146^. A more extensive and comprehensive bidirectional approach to source materials such as that modeled here would prove instrumental in mapping the phenomenon in the Baltic. This would include emplotting the results of a comprehensive review of the archives of both the Cardinal Vicar and Papal Sacristan in Rome—both records of individual supplications and copies of Authentic certificates—against church inventories for dioceses from the former Grand Duchy.

By way of conclusion, we offer reflection on some overarching themes that emerge from the study of ‘Baltic catacombs,’ whereby the translation of *corpisanti* exposes the reliance of a self-styled center (papal Rome) on its so-called artistic periphery (granducal Lithuania and Livonia) ^147^. Delimited by common boundaries of relic exchange, those involved mapped a virtual territory beyond themselves, their promulgation of complimentary models of religiostity embodied by their built environment delimiting the notional survival of a transregional Roman *imperium* as ideological relic consecrated in discursive practices that synchronically reached back through history to reaffirm retroactively both the Baltic region’s Catholic crusader origins and common dynastic interests. Attending to *corpisanti* in the far north also productively problematizes enduring ideas related to cultural transfer where the Baltic is persistently construed as recipient rather than source ^148^. We might view these various ritual movements through the discursive lens of boreal frontier-making, whereby the translated presence of the earliest Christian martyrs’ relics recast the territories of Polish Livonia and the Grand Duchy as essentially untamed heathen lands ripe for Occidentalizing by means of colonizing missionary efforts of the long Counter-Reformation, which in turn promised new martyrdoms. From this perspective, the translation to the north of *corpisanti* might be framed in terms of colonial occupation by an alien force—the ranks of ancient ‘soldiers of the faith’ sent out into the farthest reaches to conquer and civilize to the glory of Catholicism, the papacy, and Rome. We might, on the other hand, view these translations in terms of self-fashioning on the part of Commonwealth elites by means of symbolic and actual *translatio imperii*, whereby Rome’s glory and divine aura were displaced to the north. From this point of view, the infiltration of the north with catacomb relics might instead be reframed in terms of the tapping into and draining Rome of its symbolic political and actual sacral power. By tapping into the Roman catacomb relic ‘circuit’ coursing with supernatural charge, to effectively reroute and drain that same circuit, relic recipients in the Baltic and elsewhere potentially subverted both conventional center-periphery and colonial relationships.

The very physical and discursive mutability of relics as religious, political and cultural indexical signs evinces their continuing and evolving role as not only symbols of the region’s shared past (for Catholics and non-Catholics alike) but also diplomatic agents in shaping the region’s culturally distinct representation within larger everchanging European nation-states, suggesting a relationship between the figure of saints and that of states, wherein a story of mutation is echoed in both sites, whereby death of a saint is used to revitalize life of a state through repeated display. Here the architecture of sacred ritual-as-diplomacy gets folded into many forms of governmentality, religiosity, spirituality, and affect at different historical moments to suit different agendas, collective and individual. Beginning from St. Victor’s translation from Rome, we can retrace the role played by his relic-sculpture as a necropoliticised site inflecting the vicissitudes of numerous entities and their inter-relations across and between various courtly, regional, and international settings, from which emerges a mode of relationality between saint and state, demonstrating that the changing conditions of the bodies of the Baltic *corpisanti* maps onto the changing condition of Poland-Lithuania, and its relations with the Holy See, throughout the endurance and disaggregation of the territories of both at the turn of the nineteenth century ^149^.

## Data availability

### Underlying data

All data underlying the results are available as part of the article and no additional source data are required.

### Extended data

Zenodo: EC Open Research Europe Appendices 'Baltic catacombs.' Translating corpisanti catacomb relic-sculptures between Rome, Polish Livonia, and the Lithuanian Grand Duchy circa 1750–1800.

https://doi.org/10.5281/zenodo.4562735 ^150^

This project contains the following underlying data:

- Appendices-Ruth Sargent NOYES et al-Baltic Catacombs-Open Research Europe.pdf (This file comprises a clearinghouse of relevant archival manuscript and published primary source texts transcribed in their original languages and in English translation.)

Data are available under the terms of the Creative Commons Attribution 4.0 International license (CC-BY 4.0).

## Footnotes

^1^ Aspects of this research have been presented at the Yale University Program in Baltic Studies, Kunsthistorisches Institut Florenz, and Ernst-Moritz-Arndt-Universität Greifswald IRTG “Baltic Borderlands: Shifting Boundaries of Mind and Culture in the Borderlands of the Baltic Sea Region.” Special thanks to Dr. Bradley Woodworth and Dr. Harvey Goldblatt, Yale University; Dr. Gerhard Wolf and Dr. Alessandro Nova, Kunsthistorisches Institut Florenz; and Dr. Michael North and Dr. Alexander Drost, Ernst-Moritz-Arndt-Universität Greifswald. Preliminary research has also been presented at international conferences, including *The Migration of Artists and Architects in Central and Northern Europe 1560–1900* (Art Academy of Latvia, 2019), *Global Religious Translation in the Early Modern Period* (Gotha Research Centre of the University of Erfurt, 2019), *VI Seminario Internacional de Arte y Cultura en la Corte: Redes artísticas, circulación y exposición de reliquias en el Mundo Hispánico* (IULCE—Departamento de Historia y Teoría del Arte (UAM)—Casa de México, Madrid, 2019), *Splendid Encounters VII: Conflict and Peacemaking in Diplomacy, 1300–1800* (Vilnius, 2018), *European Architectural History Network International Meeting* (National Library of Estonia, Tallinn, 2018), *Visual and Material Culture Exchange across the Baltic Sea Region, 1772–1918* (Ernst-Moritz-Arndt-Universität Greifswald, 2017).

^2^ Rome, Archivio Storico della Diocesi di Roma, Archivio Storico del Vicariato di Roma, Archivio del Custode delle SS. Reliquie, Volume 77 (1737–83), “Custodia delle S.S.Reliquie dell’Imo Sigr Card. Vicario di N.S.|Corpi, e Reliquie de’ SS. Martiri Donati|Tomo I. Dall’anno 1737-al 1783|Giacinto Ponzetti Custode,” 30 April 1778, 2 fols, r-v. Hereafter citations from this archive will be given as ACSR, volume number, and date, as the folios are unnumbered.

^3^ A note regarding the rendering of proper names of persons in this article: in most cases the forms used in the period under study have been adopted, inlcuding predominantly Polish and Italian forms found in period sources, with the exception of cases where there is strong tradition of use in English, e.g. the names of pontiffs Clement, Pius, etc. On Borch see Tadeusz Turkowski, “Borch Michał Jan,” *Polski Słownik Biograficzny* (hereafter: *PSB*). Krakow: Polska Akademia Umiejętności – Skład Główny w Księgarniach Gebethnera i Wolffa, 1936, 2:313–314. For citations from the *PSB*, when available the online version is indicated. See also Janis Stradins, “O Janie Michale Borchu,” *Kwartalnik Historii Nauki i Techniki* XV (1980): 481–499. On Borch’s Grand Tour see Paweł Jeziorski, “Podróże edukacyjne zamożnej szlachty z województwa inflanckiego w XVIII wieku. Przykład Borchów i Hylzenów,” *Miscellanea Historico-Archivistica* 24 (2017): 11–24. For Borch’s account of his tour in correspondence to his father see Lviv, Vasyl Stefanyk National Academic Library of Ukraine (hereafter VSNALU), Fond 13, “Archiwum Borchów z Warklan,” file 90; for his meeting with Voltaire see Ibid., file 90, fols. 104r-111v (letter of 27 January 1775 from Lyon). This collection has been digitized and is available online through the Ossolineum (Wrocław), Poland at http://bazy.oss.wroc.pl/kzc/view_fond.php (accessed 13 February 2021). On Borch’s intellectual pursuits in the context of his Grand Tour see Marek Zgórniak, "Francophile et Patriote. Michel Borch à L'Académie de Lyon en 1775," *Zeszyty Naukowe Uniwersytetu Jagiellońskiego. Prace Historyczne* 98 (1992): 137–148; Idem., "Il Conte Borch dalle Diciannove Accademie e le sue ‘Lettres sur la Sicile’ (1782)," *Zeszyty Naukowe Uniwersytetu Jagiellońskiego MCXXVIII, Prace Historyczne* 110 (1994): 183–196. For Appendix 1 see *EC Open Research Europe Appendices 'Baltic catacombs.' Translating corpisanti catacomb relic-sculptures between Rome, Polish Livonia, and the Lithuanian Grand Duchy circa 1750–1800*, https://doi.org/10.5281/zenodo.4562735.

^4^ Regarding toponymics, in most cases state or international toponymics, rather than historical or traditional, are adopted here, by and large using contemporary titles for place names in their English version, e.g. “Vilnius,” “Rome,” and “Warsaw,” and in instances originally written in Cyrillic, the official Latin transcription. In cases of small townships, e.g. “Frosinone,” “Varakļāni” and “Krāslava,” usage appears according to the present-day national language, also Latinized when appropriate. In the case of place names in the Baltics, the first usage includes the historical title indicated in parentheses, usually in Polish, e.g. “Vilnius (Pol. Wilno).” If the text is an exact quote in Polish, such as in the Appendices, then the historical Polish form is preserved, e.g. “Olkeniki.”

^5^ On Polish Livonia see studies in Valentina Liepa, ed *. Comparative Studies: Latgale as a Cultural Border Zone*. Daugavpils University: Academic Press ‘Saule’, 2009; Krzysztof Zajas, *Absent Culture: The Case of Polish Livonia*. Frankfurt am Main; New York: Peter Lang, 2013; Melchior Jakubowski, “Krajobraz religijny i etniczny Suwalszczyzny, Bukowiny i Łatgalii na przełomie XVIII i XIX wieku. Lokalne społeczności a struktury państwowe i wyznaniowe,” PhD thesis, University of Warsaw, 2019. On the linguistic and other diversity in this region see Catherine Gibson, “The Polish Livonian Legacy in Latgalia: Slavic Ethnolects at the Confluence of the Baltic and Slavic Dialectal Continua,” in *The Palgrave Handbook of Slavic Languages, Identities and Borders*, Tomasz Kamusella, Motoki Nomachi and Catherine Gibson, eds. New York: Palgrave Macmillan, 2016, 57–80, including further bibliography.

^6^ A helpful introduction to the Grand Duchy is Norman Davies, *Litva: The Rise and Fall of the Grand Duchy of Lithuania: A Selection from Vanished Kingdoms*. New York: Penguin, 2013.

^7^ See Jerzy Lukowski, *The Partitions of Poland 1772, 1793, 1795*. New York: Taylor and Francis, 2014.

^8^ For distinctions between the terminology of *corpi santi* to refer to catacomb relics generally, and *corpisanti* to designate relic-sculptures specifically, see below.

^9^ The terminology “relic-sculpture” has been formulated by the primary author of the present study. For further discussion regarding this designation versus “ceroplastic relics” commonly used in the scholarship see below.

^10^ On this phenomenon see the many multilingual studies by leading scholar Massimiliano Ghilardi, especially the recent monograph, *Il Santo Con Due Piedi Sinistri: Appunti Sulla Genesi dei Corpisanti in Ceroplastica*. Città Di Castello: LuoghInteriori, 2019. His additional studies on this topic include: "Le Simulacre Du Martyre: Fabrication, diffusion et dévotion des corps saints en céroplastie," *Archives De Sciences Sociales Des Religions* 183 (2018): 167–87; “La fabbrica dei martiri nella Roma di fine Settecento,” *Studi Romani* I, 2, 2019, 307–342. See also studies in Philippe Boutry, Pierre-Antoine Fabre, and Dominique Julia, eds. *Reliques Modernes: Cultes et Usages Chrétiens des Corps Saints des Réformes aux Révolutions*. Paris: Editions De L'Ecole Des Hautes études En Sciences Sociales, 2009 and Stéphane Baciocchi and Christophe Duhamelle, eds. *Reliques Romaines: Invention et Circulation des Corps Saints des Catacombes à L'époque Moderne*. Rome: École Française De Rome, 2016. See also Frans Ciappara, “Translating ‘Holy Bodies’ (Corpi Santi) in Malta, 1667–1795,” *Historical Reflections / Réflexions Historiques*, 43, no. 3 (2017):1–17. For the phenomenon in the colonial Americas see Montserrat A. Báez Hernández, “The Corpi Santi under the Government of Pius VI, Materiality as a Sign of Identity: First Approaches to Novohispanic Cases,” in Strydonck, Mark Van, Jeroen Reyniers, and Fanny Van Cleven, eds. *Relics @ the Lab: An Analytical Approach to the Study of Relics*. Leuven: Peeters, 2018, 21–42 and Michel Dahan, "From Rome to Montreal: Importing Relics of Catacomb Saints Through Ultramontane Networks, 1820–1914," *Histoire sociale/Social history* 51, no. 104 (2018): 255–277. See further citations below.

^11^ For a multifaceted exploration of this art form see Montserrat A. Báez Hernández, "El cuerpo relicario: mártir, reliquia y simulacro como experiencia visual," in Rafael Mahiques and Sergi Domènech, eds. *Valor discursivo del cuerpo en el barroco hispánico*. València: Universitat de València, Anejos de Imago, 2015, 323–334. See also below.

^12^ For Poland-Lithuania in the eighteenth century see Jerzy Lukowski, *Liberty's Folly: The Polish-Lithuanian Commonwealth in the Eighteenth Century*. London: Routledge, 1991; Idem., *Disorderly Liberty: The Political Culture of the Polish-Lithuanian Commonwealth in the Eighteenth Century*. London: Bloomsbury, 2010. For the early modern Baltic region see David Kirby, *Northern Europe in the Early Modern Period. The Baltic World 1492–1772*, London and New York, Longman, 1990.

^13^ Included as Extended data in the repository: *EC Open Research Europe Appendices 'Baltic catacombs.' Translating corpisanti catacomb relic-sculptures between Rome, Polish Livonia, and the Lithuanian Grand Duchy circa 1750–1800*, https://doi.org/10.5281/zenodo.4562735.

^14^ Władysław Konopczyński, “Borch Jan Jędrzej Józef,” *PSB*. Warsaw: Narodowy Instytut Audiowizualny, http://www.ipsb.nina.gov.pl/a/biografia/jan-jedrzej-jozef-borch (accessed 13 February 2021).

^15^ For an introduction to the culture of Polish latifundia see Paul McLean, “Patrimonialism, Elite Networks, and Reform in Late-Eighteenth-Century Poland.” *The Annals of the American Academy of Political and Social Science*, 636, 2011, 88–110.

^16^ For an exemplary case study in patronage dynamics in the Grand Duchy in this period in the case of the noble R Radziwiłł clan, see Aistė Paliušytė, "Dailininkų Mobilumas XVIII A. Lietuvos Didžiojoje Kunigaikštystėje: Jeronimo Florijono Radvilos Dvaro Pavyzdys," *Menotyra* 22, no. 4 (2015): 273–89.

^17^ On the Catholicization of the Plater, Borch and Hylzen clans see Paweł Artur Jeziorski, “Z dziejów luteranizmu w Łatgalii w tzw. czasach polskich (1561–1772),” *Odrodzenie i Reformacja w Polsce*, 63, 2019, 119–154. See also Richard Butterwick, “How Catholic Was the Grand Duchy of Lithuania in the Later Eighteenth Century?,” Central Europe, 8, 2, 2010, 123–45.

^18^ On the Borch’s and Plater’s literary pursuits and broader cultural milieu see Teresa Rączka-Jeziorska, *Romantyzm polsko-inflancki. Sylwetki, teksty, archiwa*. Warsaw: The Publishing House of the Institute of Literary Research, 2016. On the material culture of Inflanty nobility see Eadem., *Inflanckie pitoreski. Kultura dworu ziemiańskiego dawnych Inflant Polskich w XIX wieku*. Warsaw: The Publishing House of the Institute of Literary Research, 2018. On the educational background of Livonian elites see also Jeziorski, “Podróże edukacyjne zamożnej szlachty z województwa inflanckiego w XVIII wieku” and Joanna Orzeł, “Tura kawalerska Józefa Jerzego Hylzena (1752–1754),” *Silva Rerum*, 2015, https://www.wilanow-palac.pl/tura_kawalerska_jozefa_jerzego_hylzena_1752_1754.html (accessed 13 February 2021). On the phenomenon of the Polish-Lithuanian Grand Tour in this period see essays in Agata Roćko, ed. *Polski Grand Tour w XVIII i początkach XIX wieku.* Warsaw: Regionalna Muzeum Pałacu Króla Jana III w Wilanowie, 2014.

^19^ On architecture in Polish Livonia in this period see above all the work of Rūta Kaminska, e.g. “Austrumlatvijas rekatolizacija un tas ietekmetais baznicu arhitekturas un makslas mantojums,” *Materiali Latvijas makslas vesturei* 7 (2008): 31–46; “The Re-Catholisation of Eastern Latvia and its Influence upon the Churches in Polish Livonia,” in *Art and the church: religious art and architecture in the Baltic region in the 13th – 18th centuries*, ed. Krista Kodres. Tallinn: Eesti Kunstiakadeemia, 2008, 280–300; “The Late Baroque Church Interiors of Livonia within pre-Partition Poland,” *Biuletyn Historii Sztuki* 73, no. 3–4 (2011): 453–478.

^20^ On the nobility in Polish Livonia during the crucial transitional period of the First Partition see recent material in Bogusław Dybaś, Paweł Artur Jeziorski, and Tomasz Wiśniewski, eds. *Szlachta polsko-inflancka wobec przełomu: materiały z dyneburskich akt grodzkich i ziemskich z lat 1764–1775*. Towarzystwo Naukowe w Toruniu. Torun: Towarzystwo Naukowe and Polska Akademia Nauk. Instytut Historii im. Tadeusza Manteuffla, 2018 and Melchior Jakubowski, “When Description means Control. The example of the Russian General Land Survey in eastern Latvia in 1784–1785,” *Landscape History* 42 (2021): in press.

^21^ On this concept see Paul Srodecki, *Antemurale Christianitatis: Zur Genese der Bollwerksrhetorik im östlichen Mitteleuropa an der Schwelle vom Mittelalter zur Frühen Neuzeit*. Husum: Matthiesen Verlag, 2015.

^22^ Simon Ditchfield, “Martyrs on the Move: Relics as Vindicators of Local Diversity in the Tridentine Church,” in *Martyrs and Martyrology*, ed. Diana Wood. Woodbridge: Boydell Press, 1993, 283–294.

^23^ Guy Lazare, “Possessing the Sacred: Monarch and Identity in Philip II’s Relic Collection at the Escorial,” *Renaissance Quarterly* 60 (2007): 58–93.

^24^ Ruth Sargent Noyes, *Peter Paul Rubens and the Counter-Reformation crisis of the Beati moderni.* New York: Routledge, 2018.

^25^ Riccardo Gennaioli and Maria Sframeli, eds. *Sacri Splendori: Il Tesoro Della 'Cappella Delle Reliquie' in Palazzo Pitti*. Livorno: Sillabe, 2014.

^26^ Anna Sylwia Czyż, “Pamięć o poprzednikach i kłótnie z kapitułą, czyli o działalności biskupa Mikołaja Stefana Paca na rzecz skarbca katedry wileńskiej,” *Humanities and Social Sciences* 23 (2018): 9–30. On this saint’s cult see also Sigita Maslauskaitė-Mažylienė, *Šventojo Kazimiero atvaizdo istorija XVI–XVIII a.* Vilnius: Lietuvos nacionalinis muziejus, 2010.

^27^ Françoise Le Hénand, “Les translations de reliques en France au xviie siècle,” in *Reliques modernes*, 313–369, at 338–339.

^28^ Ruth Sargent Noyes, “‘ *a favoririmi nel desiderio d’una Reliquia…’* (Re)moving relics and performing gift exchange between early modern Tuscany and Lithuania.” In *Gifts and Materiality: Gifts as Objects in Medieval and Early Modern Europe*, ed. Gustavs Strenga and Lars Kjar. London: Bloomsbury, forthcoming. For aspects of this exchange see also Sigita Maslauskaitė-Mažylienė, ed. *Masterpieces of the history of the veneration of St. Casimir: Lithuania – Italy*. Vilnius: Bažnytinio paveldo muziejus, 2018.

^29^ For an excellent recent study of this cultural aspect of relics see Helen Hills, *The Matter of Miracles: Architecture and Holiness in Baroque Naples*. Manchester: Manchester University Press, 2015.

^30^ See Cynthia J. Hahn, *Strange Beauty: Issues in the Making and Meaning of Reliquaries, 400-circa 1204.* University Park: Pennsylvania State University Press, 2012, 8–9.

^31^ A recent helpful theoretical overview of relics research with bibliography can be found in Georges Kazan and Tom Higham, “Researching relics: new interdisciplinary approaches to the study of historic and religious objects,” in *Life and cult of Cnut the Holy The first royal saint of Denmark*, ed. Steffen Hope *et al*. Odense: Syddansk Universitetsforlag, 2019, 142–67.

^32^ Bradford A. Bouley, *Pious Postmortems: Anatomy, Sanctity, and the Catholic Church in Early Modern Europe*. Philadelphia: University of Pennsylvania Press, 2017, 70–90.

^33^ Piero Bugiani, “From Innocent III to Today—Italian Interest in the Baltic,” *Journal of Baltic Studies* 38, no. 2 (2007): 255–62.

^34^ Stéphane Baciocchi, Philippe Boutry, Christophe Duhamelle, Pierre-Antoine Fabre, Dominique Julia, “La distribution des corps saints des catacombes à l’époque moderne, de Rome aux Nations,” in Jean-Paul Zuniga, ed. *Les pratiques du transnational, terrain, preuves, limites*. Paris: Bibliothèque de Recherches Historiques, 2011, 101–120. See also studies in Boutry, Fabre, and Julia, *Reliques Modernes*.

^35^ Massimiliano Ghilardi, “Miniere di santità. La riscoperta delle catacombe romane: oratoriani o gesuiti?,” in *La Mémoire Des Saints Originels Entre XVIe Et XVIIIe Siècle*, ed. Bernard Dompnier and Stefania Nanni. Rome: École Française De Rome, 2019, 377–397. See also Idem., *Saeculum Sanctorum: Catacombe, Reliquie E Devozione Nella Roma Del Seicento*. Città Di Castello: LuoghInteriori, 2020.

^36^ Philippe Boutry, “Les saints des catacombes: Itinéraires français d’une piété ultramontaine (1880–1881),” *Mélanges de l’école française de Rome, Moyen-Âge, Temps Modernes* 91, no. 2 (1979): 875–930. For studies on catacomb relics in other northern European borderland and frontier zones see Trevor Johnson, “Holy Fabrications: The Catacomb Saints and the Counter-Reformation in Bavaria,” *The Journal of Ecclesiastical History* 47, no. 2 (1996): 274–97 and studies in *Reliques Romaines*, including Baciocchi and Duhamelle, “Les reliques romaines hors la ville, en quel lieu que ce soit du monde,” 1–100; Marie-Elizabeth Ducreux, “«Propager la gloire des saints dans des provinces si fort éloignées de Rome». L’expansion des reliques des catacombes en Europe centrale et orientale,” 287–370; and Christophe Duhamelle and Stéphane Baciocchi, “Des gardes suisses à la frontière confessionnelle: apothéose et banalisation des corps saints des catacombes (Suisse, XVIIe-XVIIIe siècles),” 371–411.

^37^ Tojana Račiūnaitė, “Šv. Bonifaco Kankinio Relikvijos Valkininkų Bažnyčioje,” *Menotyra*, 35, no. 2 (2004): 36–45. The busts are currently in the collection of the Church Heritage Museum, Vilnius.

^38^ Sincere thanks to Father Rinalds Stankēvičs and Rūta Kaminska for assistance in accessing this material.

^39^ Rūta Janonienė, “Šventųjų relikvijų kultas Vilniaus Bernardinų bažnyčioje,” in *Šventųjų relikvijos Lietuvos kultūroje*, ed. Tojana Račiūnaitė. Acta Academiae Artium Vilnensis 41. Vilnius: Vilniaus dailės akademijos leidykla, 2006, 21–33.

^40^ Likewise in Church Heritage Museum, Vilnius. On *pasta di reliquie* see studies in Eve Borsook, ed. *Fantasia in Convento: Tesori Di Carta e Stucco Dal Seicento All'Ottocento*. Florence: Polistampa, 2008, esp. 101–178.

^41^ Ryszard Mączyński, “Włoscy męczennicy pod niebem Północy: Rozważania nad problematyką importu i kultu relikwii świętych,” in *Artyści włoscy w Polsce XV-XVIII wiek*, ed. Juliusz Chrościcki. Warsaw: Inst. Historii Sztuki Uniw. Warszawskiego, 2004, 87–142. Some instances of surviving relics in present-day Latgale (Latvia) can be found in the important series on regional church architectural momnuments by Rūta Kaminska and Anita Bistere: *Sakrālās arhitektūras un mākslas mantojums Daugavpils rajonā*. Rīga: Neputns, 2006; Eaedem., *Sakrālās arhitektūras un mākslas mantojums Rēzeknes pilsētā un rajonā*, Rīga: Neputns, 2011; Eaedem., *Sakrālās arhitektūras un mākslas mantojums vēsturiskajā Preiļu rajonā*, Rīga: Neputns, 2013; Eaedem., *Sakrālās arhitektūras un mākslas mantojums vēsturiskajā Krāslavas rajonā*. Rīga: Neputns, 2015. Also relevant are Stanisław Litak, ed., *Akta wizytacji generalnej Diecezji Inflanckiej i Kurlandzkiej czyli Piltyńskiej z 1761*. Torun: Towarzystwo Naukowe w Toruniu, 1998; Marian Radwan, *Repertorium wizytacji kościołów i klasztorów w archiwach Petersburskiego Kolegium Duchownego: (1797–1914).* Lublin: Instytut Europy Środkowo-Wschodniej, 1998.

^42^ For all these cases see Liudas Jovaiša, “Šventųjų relikvijos ir jų gerbimas Lietuvoje (1387–1655),” in *Šventieji vyrai, Šventosios moterys: šventųjų gerbimas LDK XV–XVII a*., ed. Mindaugas Paknys. Vilnius: Aidai, 2005, 185–228.

^43^ See Vilnius, Wroblewskis Library of the Lithuanian Academy of Sciences Archives, F273–388.

^44^ Ghilardi, “Miniere di santità.” See also Irina Oryshkevich. "Roma Sotterranea and the Biogenesis of New Jerusalem," *Res* 55–56, no. 55/56 (2009): 174–81.

^45^ Antonio Ferrua, “Il decreto dell’anno 1668 sull’estrazione dei corpi santi dalle catacombe,” *Rendiconti. Pontificia Accademia Romana di Archeologia* 22 (1946/47): 319–324. On market prices for catacomb relics see the c. 1662–1667 manuscript by Ambrogio Landucci, “Pratica per estrarre li Corpi de’ Santi Martiri da Sagri Cimiteri di Roma,” Rome, Biblioteca Apostolica Vaticana (hereafter BAV), Vat. Chig. G. III 82, fols. 1–55; in Ghilardi, *Saeculum Sanctorum*, 177–191, esp. 183.

^46^ Rome, BAV, ms. Vat. Lat. 9498, “De coemeteriis, cap. II: Martyres Romae coronatos non solum ad plura centena millia, sed ad incredibilem numerum pervenisse,” fol. 1–23; in Antonio Ferrua, *Sulla questione del vaso di sangue. Memoria inedita di Giovanni Battista de Rossi*. Vatican City: Pontificio Instituto Di Archeologia Cristiana, 1944, 63–98, at 72 and Ghilardi, “Paolino e gli altri martiri,” 110n29. See also Ghilardi, “La fabbrica dei martiri nella Roma di fine Settecento,” 309.

^47^ Baciocchi and Duhamelle, “Les reliques romaines hors la ville,” 12 and 98, fig. 7.

^48^ On the Sacristan see Massimiliano Ghilardi, “Il Sacrista, il pittore e le reliquie. Una lettera inedita di Angelo Rocca, Praefectus Sacrarii Apostolici,” *Analecta Augustiniana* 76 (2013): 131–150 and Jean-Marc Ticchi, “Mgr sacriste et la distribution des reliques des catacombes dans l'espace italien,” in *Reliques romaines*, 175–223. On the Custodian see Boutry, “Les saints des catacombes,” 879–881 and Nicolò Antonio Cuggiò, “Du custode des saintes reliques: traduit de l'italien et présenté par Pierre-Antoine Fabre,” in *Reliques romaines*, 119–130. For a helpful table with names and dates of those holding these respective offices from the mid-seventeenth through mid-eighteenth century see Ticchi, “Mgr sacriste et la distribution des reliques des catacombes dans l'espace italien,” 195; for a breakdown of how the Sacristan distribued relics through channels at the papal court see *Ibid.*, 198–199; for statistics for distribution in the seventeenth century according to pontificates see *Ibid*., 203. Massimiliano Ghilardi, “Le custode des reliques et des cimetières, in *Reliques politiques*, ed. Albrecht Burkardt and Jérôme Grévy. Rennes: Presses universitaires de Rennes, 2020, 145–157; Idem., “Il Custode delle Reliquie e dei Cimiteri,” *Studi Romani* I, 1, 2019, 175–210.

^49^ See Boutry, “Les saints des catacombes,” 884–885. On the rate of success of preserved supplications see *Ibid*. That Borch addressed himself to representatives of the Cardinal Vicar and not the pope’s own Sacristan suggests that the latter either could not deliver the coveted relics given extraordinary demand, or could not deliver them in time before the count’s departure.

^50^ For more on Pius’s role in the cult of catacomb relics see below.

^51^ See William H. O'Neill, *Papal Rescripts of Favor*. Washington: Catholic University of America Press, 1930, 65–67.

^52^ On authenticating catacomb relics see Massimiliano Ghilardi, "Quae Signa Erant Illa, Quibus Putabant Esse Significativa Martyrii ?," *Mélanges De L'Ecole Française De Rome. Italie Et Méditerranée*, no. 122–1 (2010): 81–106.

^53^ On this discursive convention see Boutry, “Les saints des catacombes,” 884–885.

^54^ Today the Catacomb of Priscilla is best known as a repository of early Christian art. See Sandro Carletti, *Guida delle catacombe di Priscilla*, ed. Carlo Carletti. 2nd Ed. Vatican City: Pontificia Commissione di Archeologia Sacra, 1981; Fabrizio Bisconti, Barbara Mazzei and Raffaella Giuliani, *La catacomba di Priscilla. Il complesso, i restauri, il museo*. Todi: Tau Editrice, 2013.

^55^ See e.g. Cesare Baronio, *Martirologio romano publicato per ordine della fel. mem. di papa Gregorio 13. revisto, e corretto d'ordine di papa Clemente decimo*.... Venice: Paolo Baglioni, 1702, 29.

^56^ On this practice see Boutry, “Les saints des catacombes,” 881–884.

^57^ Other successful relic petitions in the archives suggest a rate of success for petitioners who presented themselves as guardians of Catholicism in similar borderland areas. See further discussion of this factor below.

^58^ See e.g. letters to his father of 10 January 1778 in Lviv, VSNALU, Fond 13, file 90, fols. 311r-314v, where Borch complains to his father of being called “a nobody” by a papal attendant in 1778.

^59^ See letters in Ibid., fols. 309r-v and 311r-314v. On Albani see Gianni Sofri, “ALBANI, Giovan Francesco,” *Dizionario Biografico degli Italiani* (hereafter: *DBI*), 1960, https://www.treccani.it/enciclopedia/giovan-francesco-albani_(Dizionario-Biografico) (accessed 13 February 2021). On Visconti see Anton Ellemunter, *Antonio Eugenio Visconti und die Anfänge des Josephinismus: Eine Untersuchung über das theresianische Staatskirchentum unter besonderer Berücksichtigung der Nuntiaturberichte, 1767–1774*. Graz: Böhlau, 1963; Maria Gemma Paviolo, ed. *I testamenti dei cardinali: Antonio Eugenio Visconti (1713–1788)*. Morrisville, NC: Lullu Press, 2017.

^60^ Jerzy Skalski, *Rzym a sprawa polska w okresie porozbiorowym*. Warsaw: Instytut Wydawniczy Odrodzenie, 1968. For Russo-Roman Catholic relations over the longue durée see Dennis J. Dunn, *The Catholic Church and Russia: Popes, Patriarchs, Tsars, and Commissars*. Aldershot: Ashgate, 2004.

^61^ On Siestrzeńcewicz and these circumstances see André Arvuldis Brumanis, *Aux Origines de la Hiérarchie Latine en Russie: Mgr Stanislas Siestrzencewicz-Bohusz, Premier Archevêque-MétroPolitain de Mohilev (1731–1826)*. Louvain: Bureaux du Recueil, 1968.

^62^ On Archetti see Lajos Pàsztor, “ARCHETTI, Giovanni Andrea,” *DBI* 3 (1961), https://www.treccani.it/enciclopedia/giovanni-andrea-archetti_(Dizionario-Biografico) (accessed 13 February 2021). See also Paweł Zając, "Le impressioni sulla Polonia nella corrispondenza del Nuncio Apostolico Giovanni Andrea Archetti," *Archivum Historiae Pontificiae* 49 (2011): 91–121.

^63^ Joseph Lins, "Mohileff," *The Catholic Encyclopedia*. Vol. 10. New York: Robert Appleton Company, 1911, http://www.newadvent.org/cathen/10428a.htm (accessed 9 February 2021).

^64^ M. J. Rouët de Journel, *Nonciatures de Russie d'après les documents authentiques*, I, *Nonciature d'Archetti, 1783–1784*. Vatican City: Biblioteca Apostolic Vaticana, 1952.

^65^ On postmortems performed on catacomb relics see Massimiliano Ghilardi, “Ossa omnia integra, sanguinis plurimum. Alle origini della paleopatologia martiriale nell’età moderna,” in *Le Reliquie Di Sant'Ambrogio E Dei Martiri Gervaso E Protaso Tra Storia, Scienza E Fede: Apparuit Thesaurus Ambrosius*, ed. Carlo Faccendini and Carlo Capponi. Cinisello Balsamo, Milan: Silvana Editoriale, 2019, 226–229.

^66^ On the Authentic see Landucci, “Pratica per estrarre li Corpi de’ Santi Martiri da Sagri Cimiteri di Roma,” in Ghilardi, *Saeculum Sanctorum*, 188–89. For examples of period Authentics see Rome, Archivio Storico del Vicariato di Roma, Fondo reliquie, ff. Sciolti. See also Ghilardi, “Quae signa erant illa” and Idem., *Saeculum Sanctorum*, figs. 25–29, 33–34.

^67^ Vilnius, Wroblewskis Library of the Lithuanian Academy of Sciences Archives, LMAVB_RS F273–388 1r-2v.

^68^ Luigi Francesco Leonardo Desanctis, *Roma Papale descritta in una serie di lettere con note.* Florence: Tip. Claudiana, 1865, 172–73. See also Idem., *Popery and Jesuitism at Rome in the nineteenth century, with remarks on their influence in England. In twenty letters*. London: Wertheim and Macintosh, 1852, 87–90; Ghilardi, “Il Custode delle Reliquie e dei Cimiteri,” 208–9.

^69^ For examples of the relic *theca* see Francesca Sbardella, “Abilità manuale e resti 'sacri'. Pratica, creatività e produzione di reliquie,” in *I Saperi del fare: tecniche, abilità, culture*, ed. Gianluca Ligi. Milan: Cuem, 2007, 31 - 55. For a *baule* of this period see Ghilardi, *Saeculum Sanctorum*, fig. 13.

^70^ Cynthia J. Hahn, *The Reliquary Effect: Enshrining the Sacred Object*. London: Reaktion Books, 2017.

^71^ While not an academic work, the following study includes excellent photographs of the phenomenon: Paul Koudounaris, *Heavenly Bodies: Cult Treasures & Spectacular Saints from the Catacombs*. London: Thames & Hudson, 2013. For further bibliography see also Urs Amacher, "’Wir haben dem Pater Elektus den heiligen Leib des römischen Märtyrers Felix zum Geschenk gemacht’: die als Authentik bezeichnete Echtheitsurkunde für Katakombenheilige,” *Traverse: Zeitschrift für Geschichte = Revue d'histoire* 24 (2017): 170–178.

^72^ On the end of catacomb relic distribution in the mid-nineteenth century see Massimiliano Ghilardi, "The Roman Catacombs in the Nineteenth Century," in *Nineteenth-Century European Pilgrimages*, ed. Antón Pazos. Milton: Taylor and Francis, 2020, 46–61.

^73^ On these effects see P. Gerson, “Body-Part Reliquaries and Body Parts in the Middle Ages,” *Gesta* 36, no. 1 (1997): 1–7. See also Helen Hills, *The matter of miracles*, 410–445.

^74^ See Mario Rosa, “CLEMENTE XIV, papa,” *DBI*, 1982, https://www.treccani.it/enciclopedia/papa-clemente-xiv_(Dizionario-Biografico) (accessed 13 February 2021) and Marina Caffiero, “PIO VI, papa,” *DBI*, 2015, https://www.treccani.it/enciclopedia/papa-pio-vi_(Dizionario-Biografico) (accessed 13 February 2021), respectively.

^75^ Studies on the various aspects of these efforts include Hanns Gross, *Rome in the Age of Enlightenment. The Post-Tridentine Syndrome and the Ancien Regime*. Cambridge: Cambridge University Press, 1990; Jeffrey Collins, *Papacy and Politics in Eighteenth-Century Rome: Pius VI and the Arts*. New York: Cambridge University Press, 2004; Rebecca Messbarger, Christopher M.S. Johns, and Philip Gavitt, eds. *Benedict XIV and the Enlightenment: Art, Science, and Spirituality*. Toronto: Toronto University Press, 2016.

^76^ See Collins, *Papacy and Politics in Eighteenth-Century Rome*; Messbarger, Johns, and Gavitt, *Benedict XIV and the Enlightenment*; Renato Mammucari, *Settecento Romano: Storia, Muse, Viaggiatori, Artisti*. Perugia: Edimond, 2005; Carole Paul, *The Borghese Collections and the Display of Art in the Age of the Grand Tour*. Aldershot: Ashgate, 2008; Heather Hyde Minor, *The Culture of Architecture in Enlightenment Rome*. State College: Pennsylvania State University Press, 2010; Jeffrey Collins, “Museo Pio-Clementino, Vatican City: Ideology and Aesthetics in the Age of the Grand Tour.” In *The First Modern Museums of Art: The Birth of an Institution in Eighteenth- and Early Nineteenth-Century Europe*, ed. Carole Paul. Los Angeles: J. Paul Getty Museum, 2012, 112–143; Alvar González-Palacios, *Luigi Valadier*. New York: The Frick Collection, 2018.

^77^ Daiva Mitrulevičiūtė, ed. *Spalvų alchemija: skaljolos meno kūriniai iš Bianco Bianchi kolekcijos Florencijoje*. Vilnius: Išleido Nacionalinis muziejus Lietuvos Didžiosios Kunigaikštystės valdovų rūmai, 2015.

^78^ Michał Jan Borch, *Lythologie sicilienne ou connaissance de la nature des pierres de la Sicile suivie d'un discours sur la Calcara de Palerme par Monsieur le comte de Borch*. Rome: ches Benoit Francesi, 1778, v-x.

^79^ For many of these initiatives see See Mario Casaburi, *Fabrizio Ruffo: l'uomo, il cardinale, il condottiero, l'economista, il politico*. Soveria Mannelli (Catanzaro): Rubbettino, 2003.

^80^ Gross, *Rome in the Age of Enlightenment*, 152–74. On development of the Pontine Marshes over the longue durée see Anatolio Linoli, "Twenty-six Centuries of Reclamation & Agricultural Improvement on the Pontine Marshes," in *Integrated Land and Water Resources Management in History*, ed. Christof Ohleg. Schriften der Deutschen Wasserhistorischen Gesellschaft (DWhG) Sonderband 2, 2005: 27–56. On cultural aspects of the revitalization of this project under Mussolini see Federico Caprotti and Maria Kaïka, "Producing the Ideal Fascist Landscape: Nature, Materiality and the Cinematic Representation of Land Reclamation in the Pontine Marshes," *Social & Cultural Geography* 9, no. 6 (2008): 613–34; Suzanne Stewart-Steinberg, “Grounds for Reclamation: Fascism and Postfascism in the Pontine Marshes” *differences* 27, no. 1 (2016): 94–142.

^81^ Guiseppe de Novaes, *Elementi della storia de' sommi pontefici: da San Pietro sino al felicemente regnante Pio Papa VII*. 17 vols. Rome: Presso Francesco Bourlie, 1821, 17: 203–4.

^82^ See Fabiana Console e Marco Pantaloni, “Un prelato prestato alla geologia: Giuseppe Morozzo della Rocca e l’alabastro di Civitavecchia,” *Geoitaliani* 6 January 2014, https://www.geoitaliani.it/2014/01/un-prelato-prestato-alla-geologia.html (accessed 13 February 2021). See also Novaes, *Elementi della storia de' sommi pontefici*, 17: 203.

^83^ Collins, *Papacy and Politics in Eighteenth-Century Rome*, 21. See also Gross, *Rome in the Age of Enlightenment*, 172–73.

^84^ See Casaburi, *Fabrizio Ruffo*, 39–40. See also Stefania Piersanti, "‘Esiste nella città di Roma (...) una grandiosa estenzione di fabbriche’: piante ottocentesche, vicende proprietarie e storia produttiva di un complesso di opifici al Gianicolo,” in *In presentia mei notarii: piante e disegni nei protocolli dei Notai capitolini (1605–1875)*, ed. Orietta Verdi. Rome: Ministero per i beni e le attività culturali, Direzione generale per gli archivi, 2009, 227–261. For paper production in Rome see Augusto Ciuffetti, “Il commercio degli stracci da carta nello Stato pontificio nei secoli XVIII e XIX tra politiche economiche e pratiche mercantili,” *Mélanges de l’École française de Rome - Italie et Méditerranée modernes et contemporaines* 127–1 (2015): https://doi.org/10.4000/mefrim.2161 (accessed 13 Febrary 2021). On tobacco production see Andrea Metra, *Il Mentore perfetto de' negozianti, ovvero Guida sicura de' medesimi, ed istruzione, per rendere ad essi piu agevoli, e meno incerte le loro speculazioni, trattato utilissimo: diviso in cinque tomi, e compilato da Andrea Metra*. 5 vols. Trieste: presso Wage, Fleis e Comp., 1793–1797, 5:72–3.

^85^ Gross, *Rome in the Age of Enlightenment*, 152–74. See also Antonio Saltini, “L'atto di morte di un cimelio millenario: il ‘Moto proprio’ pontificio che soppresse l'annona romana,” *Rivista di storia dell'agricoltura* 42, no. 2 (2002): 117–142.

^86^ See Mario Casaburi, *Fabrizio Ruffo*.

^87^ On Magnani see Massimiliano Ghilardi, “Antonio Magnani and the invention of corpisanti in ceroplastic,” in Owen Burke, Francesco Maria Galassi, eds. *Ceroplastics: The art of wax*. Rome, L'erma di Bretschneider, 2019, 59–66.

^88^ For the sake of clarification, it should be noted that the common period terminology *corpi santi* (“holy bodies”) also adopted by scholars today was (and is) broadly used to designate catacomb relics more broadly, generally in reference to those consisting of more complete skeletal ensembles (a majority of major bones). However, in the present study the term *corpisanti* (one word) refers to a discrete sub-category of catacomb relic-sculptures that emerged only later in the history of the cult of Roman catacomb relics around the mid-eighteenth century, in the decades just preceding Michał Jan Borch’s Roman sojourn. This emergence is detailed further in the following section below.

^89^ Pietro Piraino Papoff, *Ceroplastica : Percorso Storico E Fotografico Di Un'arte Antica*. Marsala: Navarra, 2011; Andrea Daninos, *Waxing Eloquent: Italian Portraits in Wax*. Milan: Officina Libraria, 2012; Roberta Ballestriero, Owen Burke, Francesco Maria Galassi, eds. *Ceroplastics: The art of wax*. Rome, L'erma di Bretschneider, 2019.

^90^ Báez Hernández, "El cuerpo relicario.”

^91^ Joana do Carmo Palmeirão, “Imagem-relicário de Santo Aurélio mártir pertencente à Sé Catedral do Porto. Estudo e conservação integrada das relíquias,” MA thesis, Universidade Católica Portuguesa, 2015, 157–165.

^92^ For this typology in the context of Counter-Reformation Rome see Tobias Kämpf, *Archäologie Offenbart: Cäciliens Römisches Kultbild Im Blick Einer Epoche*. Leiden: Brill, 2015.

^93^ See Ferrua, *Sulla questione del vaso di sangue*.

^94^ On the Borch palace see Jolanta Polanowska, “Michal Jan Borch and his residence in Varklani: genesis and ideological programme,” *Makslas Vesture un Teorija*, 16, 2013, 18–32; and Ojārs Spārītis, “Three Sources of Michael Johann von der Borch’s Poem ‘The Sentimental Park of Varakļāni Palace’,” *Baltic Journal of Art History* 20 (2020): 109–144.

^95^ On the decorative mural paintings see Vija Strupule, “Reflections of antique art in the interior paintings of residences and manor houses in Latvia. The second half of the 18th century – the first quarter of the 19th century,” *Baltic Journal of Art History* 3 (2011): 253–280.

^96^ On Borch’s mineralogical activities see Kazimierz Maslankiewicz, "Michal Jan Borch (1751–1810) and his activity in mineralogy and geochemistry," *Actes du XIe Congès International d'Histoire des Sciences* 4 (1968):284–86. On the Pauline metaphor of “living stones” see Ruth Sargent Noyes, *Peter Paul Rubens and the Counter-Reformation crisis of the Beati moderni*, 55–57; Eadem., “Living stones: Reinterpreting cultic spaces and structures and their description in hagiographic literature c. 1600,” in *Ein Dialog der Künste. Neuinterpretation von Architektur und die Beschreibung in der Literatur von der Frühen Neuzeit bis zur Gegenwart*, ed. Barbara von Orelli-Messerli. Petersberg: Michael Imhof Verlag, 2020, 54–72.

^97^ On related techniques see Giovanna Gaeta, “BENINTENDI,” *DBI*, 1966, https://www.treccani.it/enciclopedia/benintendi_(Dizionario-Biografico)/ (accessed 13 February 2021).

^98^ Gabriela Sánchez Reyes, José Luis Velázquez and Ana Lucía Montes Marrero, “Sanctity Via the Light of Science: Radiographic Images of Ceroplastic Reliquaries,” in *Relics @ the Lab: An Analytical Approach to the Study of Relics*, ed. Mark Van Strydonck, Jeroen Reyniers, and Fanny Van Cleven. Leuven: Peeters, 2018, 133–154.

^99^ Jacques Albin Simon Collin de Plancy, *Dictionnaire critique des reliques et des images miraculeuses*. 2 vols. Paris: Guien, 1821, II: 367. On the fabrication of relic-sculptures and period perceptions regarding their in-authenticity see Massimiliano Ghilardi, “«Une infâme jonglerie», oppure «la chose du monde la plus incroyable»? Clemente IX e le reliquie del martire Fortunato,” *Studi Romani* 1 (2020): 87–112.

^100^ Borch’s testament of 1810 mentions the *Trumna Szo Wiktorego* (“Coffin of St. Victor”) in the Varakļāni parish church. See Riga, University of Latvia Academic Library, Department of Manuscripts and Rare Books, ms. 1157. See also Polanowska, “Michal Jan Borch and his residence in Varklani,” 28.

^101^ See an undated autograph document in Lviv, VSNALU, Fond 13, File 102, fol. 243: “Notice of the subjects to be represented in the 7 paintings that I wish to be painted in Dresden for my parish church in Warkland: on the high altar: 1. The Communion 13 Rhinish feet high, 9 ft wide. 2. Birth of Christ, 12 ft by 8 ft. 3. The Baptism 12 ft. x 8 ft. 4. The Resurrection 12 x 8ft. 5 The Ascension 12 x 8. 6. St. Michael the Archangel defeating the Demon 12 x 8 ft. 7. St. Victor the Martyr before Diocletian.”

^102^ Riga, Latvian State Historical Archive, 2598.f., 1.apr., 138, fol. 2. Sincere thanks to Reinis Norkārkls for assistance in accessing these materials.

^103^ Riga, Latvian State Historical Archive, 2598.f., 1.apr., 138, 5.

^104^ Sincere thanks to Father Česlavs Mikšto, Jānis Mickevičs, and the staff of Varakļāni Regional Museum (Varakļānu novada muzejs) for facilitating access to the relics in their current situation.

^105^ On Krāslava in this period see Gustaw Manteuffel, *Krasław w Inflantach Polskich*. Warsaw: Wydawnictwo "Kroniki Rodzinnej", 1901. See also Rūta Kaminska, “Construction History of Krāslava St. Louis Church in the Historical and Artistic Context of the Region,” in *Tridento visuotinio bažnyčios susirinkimo (1545–1563) įtaka Lietuvos kultūrai. Susirinkimo idėjų suvokimas ir sklaida Vidurio Europos rytuose*, ed. Aleksandra Aleksandravičiūtė. Vilnius: Publishing House of the Lithuanian Academy of Science, 2009, 90–112.

^106^ Sincere thanks to Father Eduards Voroneckis, Reinis Norkārkls, Jānis Mickevičs, and the staff of Krāslavas vēstures un mākslas muzejs and Krāslavas novada Tūrisma informācijas centrs un Starptautiskais kulinārā mantojuma centrs for facilitating access to relevant materials and to the relics in their current situation.

^107^ On the role of the Plater clan in period politics see Paweł Artur Jeziorski, “Wydarzenia z lat 1768–1772 w województwie (księstwie) inflanckim w świetle korespondencji rodziny Broel-Platerów,” in *Litwa i jej sąsiedzi w relacjach wzajemnych (XVII-XIX w.)*, ed. Iwona Janicka and Anna Kołodziejczyk. Olsztyn-Gdańsk: Instytut Historii i Stosunków Międzynarodowych Uniwersytetu Warmińsko-Mazurskiego w Olsztynie, 2014, 25–40.

^108^ On father Jan Ludwik Plater see Andrzej Link-Lenczowski, “Plater h. własnego Jan Ludwik,” *PSB*, 1981, http://www.ipsb.nina.gov.pl/a/biografia/jan-ludwik-plater-h-wlasnego-1 (accessed 13 February 2021).

^109^ On son Kazimierz Konstanty Plater see Zofia Zielińska, “Plater (Broel-Plater) Kazimierz Konstanty,” *PSB*, 1981, https://www.ipsb.nina.gov.pl/a/biografia/kazimierz-konstanty-plater (accessed 13 February 2021).

^110^ On the Paracca (or Paracco) family of architects see Mariusz Karpowicz, *Antonio Paracca. Architetto del Rococò estremo*, Valsolda: Comune di Valsolda, 2008. See also Rūstis Kamuntavičius, Aušra Vasiliauskienė, and Stefano M. Lanza, “Lugano ežero pakrančių menininkai – Lietuvos Didžiosios Kunigaikštystės baroko kūrėjai (XVI-XVIII a.),” *Darbai ir dienos*, 61. Kaunas: Vytauto Didžiojo universitetas 2013, 233–261; Ibidem., “Gli artisti del lago di Lugano e del Mendrisiotto nel Granducato di Lituania (dal XVI al XVIII sec.),” in *Gli artisti del lago di Lugano e del Mendrisiotto nel Granducato di Lituania (dal XVI al XVIII sec.)*, ed. Giorgio Mollisi. Arte&Storia, 13, 59, agosto-ottobre 2013. Lugano: Edizioni Ticino Management, 2013, 18–63, esp. 53–61. See also the forthcoming article by Rūstis Kamuntavičius and Ruth Sargent Noyes, “Lugano lake artists in the northernmost heart of eighteenth-century Catholic baroque art: Guido Antonio Longhi and members of the Paracca family,” *Annual of the Institute of the Grand Duchy of Lithuania* 1 (2021): forthcoming.

^111^ Rūta Kaminska, “Kraslavas katolu baznicas un klostera buvvesture novada vesturisko liktenu kopsakaribas,” *Materiali Latvijas makslas vesturei* 5 (2006): 9–23.

^112^ See the album of sanguine drawings, including multiple Plater family portraits, by Castaldi in Warsaw, National Museum, Department of prints and drawings: *Album Obywateli Inflant*, Rys.Pol.12141. On Castaldi and his relations with the Plater see Rūta Kaminska, “Filipo kastaldi un viņa mantojums,” *Mākslas Vēsture un Teorija* 2 (2004): 20–28; Eadem., “Filippo Castaldi (1734–1814) and his heritage in Polish Livonia (Latgale),” in *Stan badań nad wielokulturowym dziedzictwem dawnej Rzeczypospolitej*, vol. V, ed. Wojciech Walczak and Karol Łopatecki. Białystok: Instytut Badań nad Dziedzictwem Kulturowym Europy 2013, 225–248.

^113^ Vilnius, Lithuanian State Historical Archives (hereafter LSHA), coll. 1276, reg. 2, 123, fols. 64r-65v.

^114^ The fate and whereabouts of these relics are today unknown.

^115^ That Castaldi’s petition does not survive in the archives of the Cardinal Vicar suggests his request for the Plater was made through the Papal Sacristan, whose archives have not been accessed for the present study.

^116^ See Kaminska, “Filippo Castaldi (1734–1814) and his heritage in Polish Livonia (Latgale),” and Strupule, “Reflections of antique art in the interior paintings of residences and manor houses in Latvia.”

^117^ Kaminska, “Construction History of Krāslava St. Louis Church.” On female patronage in Polish Livonia see also Radosław Budzyński‬, “Maria z Ryków Manteufflowa i jej nieznana korespondencja do syna–Gustawa Manteuffla,” *Ruch Literacki* No. 4 (349) (2018):457–477, esp. 473–-475.‬‬‬‬‬ ‬‬‬‬‬‬

^118^ Michał Nowodworski, ed. *Encyklopedja Kościelna podług teologicznej encyklopedji Wetzera i Weltego z licznemi jej dopełnieniami*, 33 vols. Warsaw: Drukarnia Czerwińskiego, 1873–1933, 5:581.

^119^ *Słownik geograficzny Królestwa Polskiego i innych krajów słowiańskich, vol. X: Rukszenice – Sochaczew*. Warsaw: nakł. Władysława Walewskiego, 1889, 566.

^120^ Zofia Zielińska, “Ogiński Tadeusz Franciszek,” *PSB*, 1978, http://www.ipsb.nina.gov.pl/a/biografia/tadeusz-franciszek-oginski-1712–1783-wojewoda-trocki (accessed 13 February 2021).

^121^ Kazimierz Bartoszewicz, “Tadeusz Ogiński, wojewoda trocki i jego pamiętnik. (1712–1783),” *Przegląd Historyczny* 18, no. 1 (1914): 51–83, at 60. See also Idem., *Tadeusz Ogiński, wojewoda trocki i jego pamiętnik*. Warsaw: W. Łazarski, 1913.

^122^ Similar to Donatus, no record has been found of Bonifacus in the archives of the Cardinal Vicar’s office, suggesting this exemplar was also obtained through the Papal Sacristan.

^123^ The first is partially transcribed and translated in Appendix 5: *Nabożeństwo do św. Fortunata za dozwoleniem kościelnej zwierzchności do druku podane i przy uroczystym wprowadzeniu ciała tegoż męczennika Jaśnie Wielmożnym Jadwidze z Załuskich i Tadeuszowi z Kozielska Ogińskim Wojewodom Trockim na Hanucie, Łuczaju, Mołodecznej etc. Hrabiom Retowskim, Babiliskim etc. Starostom Ofiarowane. Imieniem konwentu siennen-skiego od XX. Franciszkanów prowincji litewskiej roku 1772*. Vilnius: w Drukarni JKM XX. Franciszkanów, 1772. See Vilnius, Vilnius University Library, Department of Rare Books, IV-1324. The second unfortunately could not be consulted for the present study: Wiktor Stepurewicz, *Kazanie podczas uroczystey introdukcyi ciała świętego Fortunata Męczennika od Stolicy Apostolskiey przez teraźnieyszego O. S. Klemensa XIV za usilnym staraniem Jaśnie Wielmożnych Ichmćw PP. Tadeusza z Xiążąt na Kozielsku tudzież Jadwigi z Załuskich Ogińskich Wojewodów Trockich, na Mołodeczney, Hanucie, Łuczaju etc. Hrabiow, Retowskich, Babiliskich etc. Starostów, Siennenskiemu, Parafialnemu XX. Franciszkanow Kościołowi z Rzymu łaskawie pozwolonego miane w pomienionym kościele w Niedzielę trzecią po Świątkach to iest dnia 28 miesiąca Junij oraz tymże JWW. PP. Woiewodom Trockim iako fundatorom, y nayszczodrobliwszym Dobrodzieiom na znak wdzięczności imieniem całey Litewskiey y Biało-Ruskiey Prowincyi Franciszkańskiey na zwykle kapitalne obrady zebraney ofiarowane Przez X. Wiktorego Stepurewicza S. T. D. Definitora Kustodii Połockiey, Kaznodzieię Wileńskiego*. Vilnius: w Drukarni J. K. M. XX. Franciszkanow, 1772.

^124^ Bartoszewicz, “Tadeusz Ogiński, wojewoda trocki i jego pamiętnik,” 65–66.

^125^ Joanna Orzeł, “From imagination to political reality? The Grand Duchy of Lithuania as a successor of Rome in the early modern historiography (15th–18th centuries),” *Open Political Science* 1 (2019): 170–181.

^126^ Račiūnaitė, “Šv. Bonifaco Kankinio Relikvijos Valkininkų Bažnyčioje.”

^127^ Andrzej Link-Lenczowski, “Potocki h. Pilawa Józef,” *PSB*, 1984–1985, http://www.ipsb.nina.gov.pl/a/biografia/jozef-potocki-h-pilawa-hetman-wielki-koronny (accessed 13 February 2021).

^128^ *O Sprowadzeniu I złożeniu ciała Świętego Bonifacego Męczennika w Kościele Olkinickim Franciszkańskim krótka Wiadomość.* Vilnius: W Drukarni J K M. XX. Franćiszkanow, 1770. See Vilnius University Library, Rare books collection, IV-22490. Sincere thanks to Liudas Jovaiša, Tojana Račiūnaitė, Aleksandra Aleksandravičiūtė, and Arvydas Grišinas for assistance accessing relevant materials.

^129^ Sincere thanks to Sigita Maslauskaitė-Mažylienė for overseeing the transfer of Bonifacus to the Museum and subsequent study and analysis, as well as for assistance in accessing relevant materials.

^130^ This is more likely than the account given in the above-cited church inventory crediting Mychal Granovsky. On the order see Marta Męclewska *et al*., eds., *Za ojczyznę i naród: 300 lat Orderu Orła Białego*. Warsaw: Arx Regia Ośrodek Wydawniczy Zamku Królewskiego, 2005.

^131^ See Ghilardi, *Saeculum Sanctorum*, 200–201.

^132^ Katarzyna Kolendo, “Woskowe figury z relikwiami Św. Klemensa biskupa męczennika z kościoła w Kiemliszkach i Justyna męczennika z kościoła w Mosarzu,” *Sztuka kresów wschodnich* 5 (2003):163–176. Documents related to Old Miadzel and Mosar can be found in Vilnius, Cultural Heritage Center Archives of the Cultural Heritage Department of the Ministry of Culture of Lithuania (Lietuvos Kultūros Paveldo Mosklinio Centro Archyvas), F. 22, “Vilniaus vaivadijos valdyba,” Ap. 1, B. 86. On this archive see Д.В. Морозов, “Материалы по охране памятников в Западной Беларуси (1921–1939 гг.) в архивах Литвы и Польши,” *Весн. Брэсцкага ўн-та. Сер. 2, Гісторыя. Эканоміка. Права* № 2. (2017): 5–15 [Dzmitry Marozau, “Materials about Cultural Objects Protection in Western Belarus (1921–1939) in the Collections of Archives of Lithuania and Poland,” J *ournal of Brest University. Ser. 2, History. Economy. Law* 2 (2017): 5–15 (in Russian)].

^133^ On the tradition of Catholic pilgrimages in the territory of current-day Belarus, with an emphasis on the north-western region, see studies by Dzianis Filipchyk, e.g. “Пешыя пілігрымкі ў Будслаў як феномен рэлігійнага турызму Беларусі,” *Сборник тезисов докладов Республиканской научной конференции студентов и аспирантов Республики Беларусь "НИРС-2011" 18 октября 2011 г.*, *Минск*, редкол.: С. В. Абламейко [и др.]. Минск: Изд. центр БГУ, 2011, 592 [“Walking pilgrimages to Budslau as a phenomenon of religious tourism in Belarus,” in *Collection of abstracts of reports of the Republican scientific conference of students and graduate students of the Republic of Belarus "NIRS-2011" October 18, 2011, Minsk*, ed. S.V. Ablameyko *et al*. Minsk: BSU Publishing Center, 2011, 592 (in Belarusian)] and “Асноўныя сацыялагічныя характарыстыкі ўдзельнікаў каталіцкіх пешых пілігрымак на Беларусі на прыкладзе пілігрымкі Барысаў-Росіца 2011,” *Романовские чтения-9: сб. статей Международной науч. конференции.* Могилёв: УО «МГУ им. А.А. Кулешова», 2013, 180–181 [“The main sociological characteristics of the participants of Catholic walking pilgrimages in Belarus on the example of the pilgrimage Borisov-Rositsa 2011,” *Romanov readings-9: Sat. articles of the International scientific. conference*. Mogilev: UO “MSU. A.A. Kuleshova”, 2013, 180–181 (in Belarusian)].

^134^ The precise location of this starostwo remains to be identified.

^135^ For the legal basis of monument protection in Western Belarus in the period from 1921–1939 see Д.В. Морозов, “Правовая основа и организационная структура охраны памятников в Западной Беларуси (1921–1939 гг.),” *Журнал Белорусского государственного университета. История* 4 (2017): 50–56 [Dzmitry Marozau, “Legal basis and organizational structure of the monuments protection in Western Belarus (1921–1939),” Journal of the Belarusian State University. Historical sciences 4 (2017): 50–56 (in Russian)].

^136^ Kolendo, “Woskowe figury z relikwiami,” 167.

^137^ *Nabozenstwo Do S. Justyna Męczennika Chrystusowego Codziennemi Łaskami Y Cudami Słynacego Od W. Jmć Pana Antoniego De Kosein Koszczyca Starosty Zarzyckiego Do Koscioła Staro-Miadziolskiego Ww. Oo. Karmelitow Bosych Dla Rady Y Pomocy Wiernych Chrystusowych Panską Hoynoscią Wprowadzonego Ku Większey Czci Y Chwale S. Cudotworcy Y Naboznego Ludu Wygody Wydane*. Vilnius: w Typografii J. K. M. przy Akademij, 1780. See Vilnius, Vilnius University Library, Department of Rare Books, IV 23001. Also consultable online at https://kolekcijos.biblioteka.vu.lt/islandora/object/kolekcijos:VUB01_000501479#00001 (accessed 13 February 2021).

^138^ Franz Wieland, *Mensa und Confessio: Studien über den Altar der Altchristlichen Liturgie*, 2 vols. Munich: J.J. Lentner, 1906. On the *confessio* altar type in Poland in this period, see especially studies by Ryszard Mączyński, e.g. *Nowożytne konfesje polskie. Artystyczne fory gloryfikacji grobów świętych i błogosławionych w dawnej Rzeczypospolitej*. Toruń: Wydawnictwo UMK, 2003; Idem., “Konfesje – ziemskie groby niebiańskich orędowników,” *Spotkania z Zabytkami* XXIX, no. 10 (2005):8–11. For examples of *corpisanti* in the Crown territories, a topic beyond the purview of the present study, see Marek Machowski, “Bardzo krótka historia relikwiarza św. Kandyda z kościoła w Choroszczy” and Helena Hryszko, „Analiza techniki wykonania figury św. Kandyda z kościoła w Choroszczy,” both in *Biuletyn Konserwatorski Województwa Podlaskiego 10*. Białystok: Wojewódzki Urząd Ochrony Zabytków w Białymstoku, 2004, 45–94 and 95–110, respectively.

^139^ Steven Ostrow, “The ‘Confessio’ in Post-Tridentine Rome,” in *Arte e committenza nel Lazio nell’età di Cesare Baronio*, ed. P. Tosini. Rome: Gangemi, 2009, 19–32.

^140^ Noyes, *Peter-Paul Rubens*, 180–181. See also Alexandra Herz, “Cardinal Cesare Baronio’s Restoration of SS. Nereo ed Achilleo and S. Cesareo de’Appia,” *The Art Bulletin* 70, no. 4 (1988):590–620.

^141^ On these cases see Kolendo, “Woskowe figury z relikwiami,” 163–165. On Chreptowicz see Władysław Konopczyński, “Chreptowicz Karol Litawor,” *PSB*. Krakow: Polska Akademia Umiejętności, Skład Główny w Księgarniach Gebethnera i Wolffa, 1937, 3:443–444. Sincere thanks to Father Antoni Gremza, Church of the Annunciation of the Blessed Virgin Mary in Hrodna, for information on the Hrodna relics.

^142^ This record remains to be verified in the Roman archives of the Custodian.

^143^ Sincere thanks to Father Antoni Gremza, Bernardine Church of the Annunciation of the Blessed Virgin Mary in Hrodna, for furnishing a photograph of this document. Although what remains of Clemens underwent significant modifications and was moved over the course of the twentieth century, the language of *Solenna introdukcya* (which referes to the saint’s personified “body”) and early photographs of the *confessio* installation in an ornate coffin atop the altar *mensa* suggests this was originally in the form of a *corposanto* relic-sculpture.

^144^ Michał Kadłubowski, *Solenna introdukcya ciała S. Klemensa Męczennika do kościoła WW. PP. Brygittek Grodzień. gorliwością y nieporównanym kosztem nayprzewielebnieyszey Jeymć. panny Konstancyi Micucianki Xieni tegoż klasztoru, z rzadką wspaniałością, ku większey chwale y uwielbieniu w świętych pańskich Boga, ku czci męczennika Chrystusowego pomnożona trzydniowym nabożeństwem iak nayuroczyściey obchodzona y do wiadomości ludowi wiernemu z opisaniem całego aktu podana przez W. J. X. Michała Kadłubowskiego Scholastyka Prałata Infl. Wizytatora gener. Dyec. Wileńsk. Białost. Brzost. Olit. Proboszcza y na tęż uroczystość introduktora y solenizanta r. 1781. d. 3, 4 i 5 Junij*. Hrodna: w drukarni J. K. Mści, 1781.

^145^ Rome, ACSR, 77, 6 July 1778r-v.

^146^ “Opsa,” *Słownik Geograficzny Królestwa Polskiego I Innych Krajów Słowiańskich*. Warsaw: Druk "Wieku", 1890, 7: 569.

^147^ On this issue see also Tomasz Grusiecki, “Going Global? An Attempt to Challenge the Peripheral Position of Early Modern Polish-Lithuanian Painting in the Historiography of Art,” *The Polish Review* 57, 4 (2012): 3–26.

^148^ See discussion in Thomas DaCosta Kauffmann, “Baltic Reflections,” *Baltic Journal of Art History* 9 (2015):11–22.

^149^ Echoing Pamila Gupta, *The Relic State: St Francis Xavier and the Politics of Ritual in Portuguese India*. Manchester, Manchester University Press, 2014, 13–14.

^150^ Ruth Sargent Noyes. (2021, February 25). EC Open Research Europe Appendices 'Baltic catacombs.' Translating corpisanti catacomb relic-sculptures between Rome, Polish Livonia, and the Lithuanian Grand Duchy circa 1750–1800. Zenodo. http://doi.org/10.5281/zenodo.4562735

