# Supplementary material for: ‘Baltic catacombs.’ Translating corpisanti catacomb relic-sculptures between Rome, Polish Livonia, and the Lithuanian Grand Duchy circa 1750-1800
Source: Open Res Eur. 2021 Mar 24;1:18. [Version 1] doi: 10.12688/openreseurope.13259.1 (PMC10446102; doi:10.12688/openreseurope.13259.1)
Supplement: Peer review history

# Peer review: Reviewer response for version 1

**DOI:** 10.21956/openreseurope.14329.r26613

**Published:** 2021-06-02

**Authors:** Rachel Miller

**Affiliations:** 1 California State University, Sacramento, CA, USA

The authors of “Baltic catacombs: Translating *corpisanti *catacomb relic-sculptures between Rome, Polish Livonia, and the Lithuanian Grand Duchy circa 1750-1800” have presented an extremely valuable interdisciplinary and international study that serves as a model for how collaborative research can facilitate scholarship during difficult global circumstances that have limited travel and access to materials. This article is also greatly enriched by the different disciplinary approaches and linguistic abilities of each author, demonstrating the advantages of collaborative research when dealing with transnational and cross-cultural topics in particular.

 Traditionally, when writing about the Grand Tour, art historians have focused on how elite travel facilitated the circulation of antiquities that were appreciated for their aesthetic values and prompted the development of the Neoclassical movement. This article greatly nuances this narrative by presenting the example of Count Michał Jan Borch, a Grand Tourist who prioritized the acquisition of saintly relics taken from the ancient catacombs of Rome which were then fashioned into a relic-sculpture before being transported back to Borch’s estate in Polish Livonia. This study is a valuable addition to the literature on the global circulation of relics throughout the early modern world, laying bare the elite networks through which acquisition of these holy bones were facilitated and disrupting problematic ideas of center-periphery in the history of art and material culture. While this article is more than worthy of indexing, I offer a few small suggestions for improvement:

**A few areas for further explanation:**

 Pg. 8 I agree with Dr. Živković’s review that the concept of *Romanitas *requires expansion here.

 Pg. 16 “archeological authenticity.” This statement would benefit from more explanation of what it meant to be “archeologically authentic” in the late 18 ^th^ century. It seems that this sculpture, rather than accurately recreating the archeological context of the catacombs of the eighteenth century, helped maintain a fiction that the Roman catacombs were pristine sources of incorrupt bodies, untouched by the ravages of time and decay, linking the contemporary Roman Catholic Church back to its ancient, purer form. On this same page, it would also be helpful to more fully describe the *all’antica* military costumes worn by these figures.

 Pg. 20 In footnote 101, the authors describe the iconography proposed for paintings in Borch’s parish church. The inclusion of a painting of St. Michael seems significant here and I believe warrants moving this description into the main body of the article. The importance of the iconography of St. Michael as a militant vanquisher of heresy creates a very nice parallel to St. Victor’s relic-sculpture. The way that the Church and Catholic elite used images of St. Michael during the Counter-Reformation broadly is analogous in many ways to St. Victor’s *corposanto *in Polish Livonia. In addition, this page briefly mentions a heavily edited draft of the dedicatory inscription of St. Victor’s chapel, a text that seems to be strong evidence for the authors’ arguments. If possible, it would be helpful to include this text or expand upon its contents more.

**A few minor notes:**

 Pg. 1 – Footnote 3 – small typo, “inlcuding”

 Pg. 2 – Repeated phrase “of the Polish-Lithuanian Commonwealth”

 Pg. 7 – Michael Carroll’s book *Veiled Threats *proposes an additional reason for the increased importance placed on incorruptibility Post-Trent, specifically, higher standards of heroic virtue required from candidates for sainthood. This idea of “heroic virtue” might connect nicely to the types of saints, especially the male soldiers, who were exported to the Baltics.

If any, are all the source data and materials underlying the results available?

Yes

Is the work original in terms of material and argument?

Yes

Is the work clearly and cogently presented?

Yes

Is the argument persuasive and supported by evidence?

Yes

Does the research article contribute to the cultural, historical, social understanding of the field?

Yes

Does it sufficiently engage with relevant methodologies and secondary literature on the topic?

Yes

Reviewer Expertise:

The art and visual culture of Jesuit saint's cults with an emphasis on the global dissemination of images and relics in the early modern Iberian empires (particularly Portuguese Asia).

I confirm that I have read this submission and believe that I have an appropriate level of expertise to confirm that it is of an acceptable scientific standard.

# Peer review: Reviewer response for version 1

**DOI:** 10.21956/openreseurope.14329.r26619

**Published:** 2021-05-10

**Authors:** Teresa Rączka-Jeziorska

**Affiliations:** 1 Institute of Literary Research, Polish Academy of Sciences, Warsaw, Poland

The reviewed article entitled *‘Baltic catacombs.’ Translating corpisanti catacomb relic-sculptures between Rome, Polish Livonia, and the Lithuanian Grand Duchy circa 1750-1800*, **is the result of an international interdisciplinary project, directed by a Danish art historian Ruth Sargent Noyes (National Museum of Denmark, Copenhagen) and created in cooperation between its author and academics representing related disciplines (history, literary studies, culture studies) from Belarus: Dzianis Filipchyk, Dzmitry Marozau, Lithuania: Vika Veličkaitė and Poland: Radosław Budzyński, Melchior Jakubowski. It makes an original contribution to the existing research on the preserved sacral heritage at the turn of the 18th and 19th centuries in the areas once belonging to the Polish-Lithuanian Commonwealth, with particular emphasis on its northernmost borders, i.e. the former Polish Livonia (today's Latgale in Latvia).

 The Danish researcher undertook an attempt at a multilateral examination of the problem of the occurrence of the so-called corpisanti, i.e. preserved relics as a basis of a work of art with a religious and spiritual dimension, combining relics themselves and a reliquary in one sculptural form, paying attention to their historical transformations and the phenomenon of "long duration" in the culture of local and most influential Catholic elites, including representatives of the leading families of the former Polish-Lithuanian Commonwealth: such as Sapieha, Borch, Plater, Ogiński and Potocki. Thanks to the reconstruction of this phenomenon, supported by a rich illustrative and archival commentary, the author has successfully managed to show the close cultural connection of the peripheral East-Baltic North with the dynamically operating Holy See, visible, amongst other aspects, thanks to the "sacred" objects of religious worship, and owing its flourish over the centuries, in part, to the Jesuit Order.

 The modern act of pilgrimage, culminating in the witnessing of the corpisanti housed within sacral buildings in Latvia, Lithuania and Belarus cannot be detached from their noble provenance, therefore what is extremely valuable in this article is its explanation of the context of the wide variety of unknown or poorly recognized sources, pointing to their historical beginnings and wide political and ecclesiastical transference **. **It would certainly be worthwhile, however, to deepen these observations, bearing in mind the particular multicultural, multinational and multireligious specificity of the former Livonian voivodeship, as well as the strong attachment of its prominent inhabitants to the Catholic tradition (especially in the 18th and 19th centuries).

 On this occasion, it is necessary to recall the strong involvement of both the Polish-Livonian and Lithuanian nobility in establishing numerous church foundations in this area, as well as close ties, especially in the 18th and 19th centuries, (also of a social nature) with the local clergy. Perhaps the use of the term 'aristocratic clans' in the case of some families, e.g. Borch, Plater, and Hylzen, should be reflected upon as it is not adequate to the scale of their influence in the state (compared to e.g. the Sapieha, Radziwiłł, Potocki or Czartoryski families, a much wealthier, more influential part of the power elite for a much longer time).

 The context of bringing the relics of St. Victor to Livonia, with which R. Sargent Noyes starts her contemplations, seems very interesting. It is inextricably linked with the youthful journey of Count Michał Jan Borch to the south of Europe and his stay in Rome in 1778, which could also be read as an important turning-point in his biography, considering that after returning from his journeys and finally settling in Warklany, according to some researchers, he turned from a cosmopolitan and a Freemason into a zealous and devoted Catholic. However, one of the author's conclusions, namely the interpretation of the phenomenon of corpisanti: "in terms of colonial occupation by an alien force [...] to conquer and civilize to the glory of Catholicism" (p. 34) should be considered with great caution. The fact of the circulation of relics between Livonia, the Grand Duchy of Lithuania and the Eternal City, which was gaining momentum in the 18th/19th centuries, certainly no longer fulfils the missionary function in the period studied here, i.e. it does not "recast [...] untamed heathen lands" (p. 34), but makes them even more so the "lands of Mary".

 Other comments:

- the youthful journey of Michał Jan Borch started in 1774, and not in 1772, as the author states (pp. 3-4);
- the conversion of Polish-Livonian Borch family members to Catholicism had already started by the end of the 17th century, so in the case of this family the whole phenomenon should be dated for from the turn of the 17th/18th century (p. 5);
- what is meant by the term "magistrate" requires clarification, which specific office/dignity is meant in this case? During his journey Michał Jan Borch happened to use various titles illegally, presenting himself as a "major-general" (amongst other titles), although he never achieved this rank during his military "career". Maybe in this case he actually assigned himself the office illegally, or the mistake was made on the part of the Vatican officials occurred? (pp. 10-11).

If any, are all the source data and materials underlying the results available?

Yes

Is the work original in terms of material and argument?

Yes

Is the work clearly and cogently presented?

Yes

Is the argument persuasive and supported by evidence?

Yes

Does the research article contribute to the cultural, historical, social understanding of the field?

Yes

Does it sufficiently engage with relevant methodologies and secondary literature on the topic?

Yes

Reviewer Expertise:

Romantic literature (with special consideration of Polish-Livonian literature), culture and history of Old Polish Livonia, Polish-Baltic borderland, etc.

I confirm that I have read this submission and believe that I have an appropriate level of expertise to confirm that it is of an acceptable scientific standard.

# Peer review: Reviewer response for version 1

**DOI:** 10.21956/openreseurope.14329.r26610

**Published:** 2021-04-08

**Authors:** Anna Sylwia Czyż

**Affiliations:** 1 Warsaw Institute of History of Art, Cardinal Stefan Wyszyński University, Warsaw, Poland

The article in a very interesting way describing the corpisanti catacomb relic-sculptures in the Grand Duchy of Lithuania and Polish Livonia in the second half of 18th c. The topic is present not only in the religion aspect but also in the social and political context. In addition I would like to pay Authors' attention on one inconsistency worth double checking. At page 10 and fig. 7 – I’m not sure that in that reliquaries we have pasta di relique there also may be Agnus Dei (Agnuses) which are an oval or round wax medallions with a representation of a saint and were displayed in reliquaries.

If any, are all the source data and materials underlying the results available?

Yes

Is the work original in terms of material and argument?

Yes

Is the work clearly and cogently presented?

Yes

Is the argument persuasive and supported by evidence?

Yes

Does the research article contribute to the cultural, historical, social understanding of the field?

Yes

Does it sufficiently engage with relevant methodologies and secondary literature on the topic?

Yes

Reviewer Expertise:

History and art in the Grand Duchy of Lithuania.

I confirm that I have read this submission and believe that I have an appropriate level of expertise to confirm that it is of an acceptable scientific standard.

# Peer review: Reviewer response for version 1

**DOI:** 10.21956/openreseurope.14329.r26618

**Published:** 2021-04-06

**Authors:** Livia Stoenescu

**Affiliations:** 1 Department of Visualization, College of Architecture, Texas A&M University, College Station, TX, USA

Ruth Sargent Noyes’s 'Baltic catacombs.' Translating *corpisanti *catacomb relic-sculptures between Rome, Polish Livonia, and the Lithuanian Grand Duchy circa 1750-1800 is a remarkable piece of scholarship on the understudied, albeit seminal, question of the dissemination of relics from the place commonly understood to have generated the cult of relics in the Catholic World: Rome. The methodology weaves history and historiography into a coherent narrative about the endeavours of Count de Borch to obtain the Holy Body of St. Victor for the purpose of translating the catacomb relic from Rome to Baltic Europe. The author shrewdly identifies this undertaking as a characteristic of the “catacombization” of the Baltic, and estimates Borch himself as a “relic of the Polish aristocracy and defender of Catholicism after the partition”. 

 Indeed, the Counter-Reformation revived a new interest in the ancient roots of the Roman church and early Christian catacombs, resulting in a series of late sixteenth-century studies meant to compile from various documentary sources the history and final abandonment of the Roman catacombs at the end of the Middle Ages. John Osborne’s “The Roman Catacombs in the Middle Ages,” *Papers of the British School at Rome* 53 (1985): 278-328 remains an influential work in elucidating the origins of the catacombs in a time of enormous blossoming of the cult of the saints and orchestrated interest in their earthly remains. Noyes’s article begs the question: how would the Baltic territories fit into the culture of relics as the ease of Mediterranean travel declined and many pilgrims from Europe began to look to the city of Rome as an alternative to Jerusalem?

 The adaptation of relics to polychromed sculptures, relics busts, and miniatures represents a fascinating aspect of Noyes’s article, prompting a few commentaries on the new significance they attained:

 p. 9 The catacomb reliquary busts of St. Aurelius and St. Victoria contained relics in ways that seem to describe a local practice. As the author notes, the relics were “installed into polychromed sculptures or relics busts.” Would it be possible to talk about an interest in polychromatic sculptural portraiture in Baltic Europe that predated the interest in installing relics into half-length busts?

 p. 10 The reliquary bust was a mainstay of relic preservation in Western Christendom. But why would the same format appeal to the local devotions of Baltic Europe? Was the choice of miniature reliquary busts a retention of Latin Christianity? Was the Baltic interest in sacred miniature sculpture directly correlated with the objects manufactured by mixing pulverized bones into a pasta and molding this substance into miniatures? Were miniatures used as objects of devotion in Polish Livonia and the Lithuanian Grand Duchy?

If any, are all the source data and materials underlying the results available?

Yes

Is the work original in terms of material and argument?

Yes

Is the work clearly and cogently presented?

Yes

Is the argument persuasive and supported by evidence?

Yes

Does the research article contribute to the cultural, historical, social understanding of the field?

Yes

Does it sufficiently engage with relevant methodologies and secondary literature on the topic?

Yes

Reviewer Expertise:

Prof. Livia Stoenescu’s research is focused on Early Modern Art and Architecture in the Italian and Spanish peninsulas, with an emphasis on prototypal images, architectural drawing, relics, and polychromatic sculptural portraits.

I confirm that I have read this submission and believe that I have an appropriate level of expertise to confirm that it is of an acceptable scientific standard.

# Peer review: Reviewer response for version 1

**DOI:** 10.21956/openreseurope.14329.r26598

**Published:** 2021-03-29

**Authors:** Valentina Živković

**Affiliations:** 1 Institute for Balkan Studies SASA, Belgrade, Serbia

The article ' *Baltic catacombs.' Translating corpisanti catacomb relic-sculptures between Rome, Polish Livonia, and the Lithuania Grand Duchy circa 1750-1800'* by Ruth Sargen Noyes sets up to explore the invention, production and distribution of *corpisanti* catacomb relic-sculptures from papal Rome and Polish Livonia, as well as the Lithuania Grand Duchy. The article provides an excellent interdisciplinary research of the topic. The approach is highly relevant for contemporary understanding of the Catholic cult of relics and relations between the Italy and the Baltic littoral. 

 The article is original in terms of material and argument, sufficiently engages with relevant methodologies; previous research results are referred with extensive bibliography. The article is clearly presented and highly significant for the understanding of the topic.

**There are, however, several comments and minor changes I propose:**

 p. 7 - it should be noted ( is repeated several times)

- Which are often referred to not as things or relics per se, but rather as persons, bodies, or protagonists…the illusion of embodied corporality and indeed personhood – I would advise a more precise explanation of the essential differences and meaning of the relics and corpisanti.

p. 8 - The Borch ranked among the only in Polish Livonia to hold the titles of Counts of the Holy Roman Empire, and vigorously cultivated both their crusader origins and ties to the Roman Church, overseeing after the Northern Wars (c. 1650–1720) a web of monuments largely built or re-built that articulated Inflanty’s sacred topography on Europe’s north-eastern frontier - It would be significant to provide some important literature on the topic of sacred topography.

- Their cultural campaigns of self-fashioning staged a renovation of Romanitas – note on the concept and meaning of a renovation of Romanitas.
- Relics could embody power and instantiate identity on both a local and national level in this period – exactly the same concept was usual for the medieval period.
- Their heavenly avatars – it would be better to use another terminology in order to avoid confusion with Hinduism (as well as p. 22).

p. 15 Desanctis’s colorful description gives a sense of the sordid banality of the catacomb relic boom, a banality that unfolded from the dark and dank *loculi *of the catacombs mined by professional quarrymen ….and a banality camouflaged not only by the intricate courtly rituals… – rather avoid assessment *banality*, and examine the phenomenon from a neutral point of view.

 p. 17 *Corpisanti *bring together the holy relic and the reliquary container into a single sculptural form, incorporating bodily remains in the form of scant bone fragments within a three-dimensional sculpture made to approximate a human figure, outfitted and staged to appeal both to late baroque-rococo courtly tastes and neoclassical antiquarian sensibilities. – maybe is more suitable for p 10?

If any, are all the source data and materials underlying the results available?

Yes

Is the work original in terms of material and argument?

Yes

Is the work clearly and cogently presented?

Yes

Is the argument persuasive and supported by evidence?

Yes

Does the research article contribute to the cultural, historical, social understanding of the field?

Yes

Does it sufficiently engage with relevant methodologies and secondary literature on the topic?

Yes

Reviewer Expertise:

Art and religiosity in Kotor (14th – 17th centuries) with particular focus on the links between two Adriatic coasts, the cult of the saints and veneration of relics, the testamentary legacies ad pias causas and the prommotion of Dominican observant reform.

I confirm that I have read this submission and believe that I have an appropriate level of expertise to confirm that it is of an acceptable scientific standard.
